# Dynamic Reconfiguration of Pt(II) Supramolecular Assemblies via Ligand Exchange

**DOI:** 10.1021/acsami.5c10381

**Published:** 2025-09-11

**Authors:** Elisa Pelorosso, Mirco Scaccaglia, Dario Alessi, Claudia Graiff, Alessandro Aliprandi

**Affiliations:** 1 Dipartimento di Scienze Chimiche, 165501 Università degli Studi di Padova, Via Marzolo 1, Padova 35131, Italy; 2 Department of Chemistry, Life Sciences and Environmental Sustainability, 9370 Università degli Studi di Parma, Parco Area delle Scienze 17/A, Parma 43124, Italy

**Keywords:** dynamic ligand exchange, platinum(II) complexes, self-assembly, aggregates, luminescent supramolecular structures

## Abstract

Supramolecular chemistry enables molecules to dynamically adapt and reorganize in response to their environment, providing a key route to achieving high levels of structural and functional complexity. This work explores a particular strategy for the dynamic and programmable self-assembly of luminescent platinum­(II) complexes via sequential coordination-driven and hierarchical processes. The aggregation behavior and optical properties of square-planar Pt­(II) complexes bearing a chromophoric terdentate N̂N̂N ligand and exchangeable monodentate ligands are highly dependent on the nature of the ancillary ligand, resulting in morphologically and photophysically distinct supramolecular structures. We demonstrate that these preassembled aggregates undergo dynamic ligand exchange reactions in solution, leading to metastable supramolecular states, including emissive gels, that are accessible exclusively through in situ exchange. Real-time fluorescence microscopy and NMR spectroscopy reveal both homogeneous and heterogeneous exchange pathways, governed by the identity of the initial complex and the incoming ligand. Remarkably, the system exhibits a degree of reversibility and structural memory. These findings establish a framework for stepwise self-assembly that bridges coordination chemistry with noncovalent interactions, offering a versatile platform for designing responsive nanostructures with tailored properties and a step toward adaptive, life-like materials with potential applications in sensing and optoelectronics.

## Graphical abstract

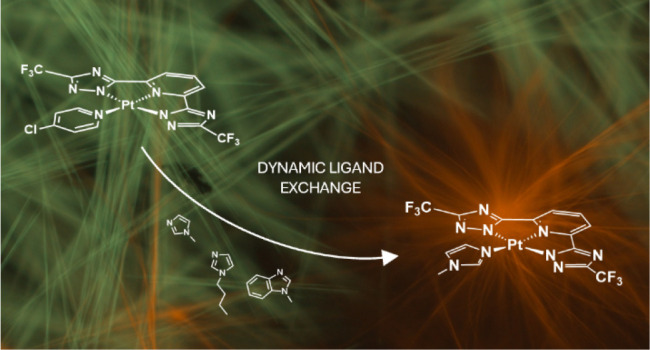

## 1 Introduction

Advances in metallo-supramolecular chemistry have unlocked new opportunities for the synthesis of functional nanosystems and intelligent materials with increasing complexity.^1^ Metal-mediated self-assembly has led to the formation of diverse structural motifs, including helicates,^2,3^ grids,^4^ mechanically interlocked structures,^5^ cycles^6,7^ and cages.^8–10^ Among these, cages have drawn particular attention due to their nanoscale cavities, which enable applications in molecular encapsulation,^11^ catalysis,^12–15^ and sensing.^16–18^ Over the last four decades, coordination-driven self-assembly (CDSA) has emerged as a powerful and versatile strategy for constructing discrete nanoscale architectures.^19–21^ This approach, rooted in the principles of coordination chemistry, involves the spontaneous organization of metal ions and carefully designed organic ligands into well-defined, three-dimensional structures. The resulting assemblies are governed primarily by the inherent coordination preferences of the metal centers and the geometric constraints of the ligands. Owing to the reversible and dynamic nature of coordination bonds,^22^ these systems can undergo self-correction during assembly, facilitating the formation of thermodynamically stable and structurally uniform products. However, it is important to distinguish between traditional CDSA, which typically focuses on the formation of discrete entities (such as metal–organic frameworks or supramolecular coordination cages), and systems where the dynamic nature of coordination bonds is exploited to generate changes within supramolecular polymers or dynamic combinatorial libraries. In fact, the latter approach, exemplified by the pioneering work of S. Otto and co-workers, emphasizes the use of reversible covalent and noncovalent interactions to create adaptive and evolvable dynamic libraries of interconverting species.^23–26^ These dynamic libraries enable the selection and amplification of specific architectures in response to environmental stimuli, thus providing a powerful platform for the discovery of functional supramolecular assemblies beyond the scope of classical CDSA.

A complementary and broader approach involves hierarchical self-assembly through noncovalent interactions, in which individual components, either purely organic molecules or preformed coordination complexes, spontaneously organize into higher-ordered structures.^27–30^ This process is driven by weak noncovalent forces such as hydrogen bonding,^31^ π–π stacking,^32^ van der Waals interactions,^33^ and electrostatic attractions,^34,35^ leading to the formation of macroscopic assemblies^36^ including gels,^37–42^ and crystalline frameworks.^43,44^ Notably, the formation of such assemblies is often accompanied by the emergence of unique properties that are not exhibited by the individual components—properties that can be particularly valuable for applications in optoelectronics.^45,46^ However, both coordination-driven and hierarchical self-assembly are typically limited to a single mixing step, where most of the structural complexity originates from the covalent synthesis of reactants rather than from a stepwise assembly process that builds complexity in a reproducible manner, as noted by Vantomme and Meijer.^47,48^ We propose that these two approaches, hierarchical and coordination-driven self-assembly, can be combined into a sequential process for the noncovalent synthesis of supramolecular objects. Specifically, a preformed metal complex can self-assemble into a supramolecular structure that subsequently acts as a template for a ligand exchange reaction, yielding a new supramolecular structure with distinct properties. Square-planar Pt­(II) complexes are particularly well suited for studying self-assembly due to their flat geometry. Monomeric Pt­(II) complexes can interact face-to-face, establishing metallophilic interactions between the dz^2^ orbitals of the platinum centers. Since the orbitals involved in these interactions are filled, such interactions lead to a destabilization of the HOMO, increasing its energy and consequently reducing the HOMO–LUMO gap.^49^ As a result, new metal–metal-to-ligand charge transfer (MMLCT) transitions become allowed in the visible range, giving rise to new optical properties such as photoluminescence. The optical behavior of the aggregated species is highly sensitive to molecular packing: metallophilic interactions are observed when the Pt–Pt distance is less than 3.5 Å. When the distance significantly exceeds the sum of the van der Waals radii of platinum, electronic interactions do not occur, resulting in a blue-shifted emission.^50^ Additionally, platinum-based compounds are well-known for their ability to undergo ligand exchange reactions.^51,52^ A classic example is the chemotherapeutic agent cisplatin, which undergoes ligand exchange upon entering cells, replacing chloride ions with water molecules.^53^ This inherent reactivity offers exciting possibilities for the development of dynamic supramolecular systems. Luminescent Pt­(II) complexes bearing a chromophoric terdentate N̂N̂N dianionic ligand and an ancillary pyridine moiety are particularly promising for this study, as their solubility and aggregation behavior can be tuned by functionalizing the pyridine ligand.^54,55^ In this work, we investigate a series of luminescent platinum complexes, demonstrating that the ancillary pyridine ligand can be exchanged with imidazole and benzimidazole derivatives in solution at room temperature. Furthermore, this ligand exchange leads to the formation of a metastable kinetic state in the form of a gel, which is accessible exclusively through in situ ligand exchange. Real-time fluorescence microscopy further reveals that this dynamic ligand exchange can also occur from preformed supramolecular structures, which act as a reservoir for the complexes—either being consumed and replaced by new structures or templating the formation of new assemblies that retain the shape of the original aggregates. These findings are crucial for advancing dynamic supramolecular chemistry, offering new strategies for designing adaptive materials with applications in drug delivery, sensing, and molecular electronics. By demonstrating a stepwise approach to self-assembly, this work bridges the gap between coordination-driven and hierarchical self-assembly, paving the way for reconfigurable nanostructures and responsive materials.

## 2 Methods

The synthetic strategies used for all complexes, along with their full characterization, are provided in the Supporting Information. Details of the characterization techniques are described below.

### 2.1 Photophysical Measurements

Absorption spectra were recorded and baseline-corrected using a Varian Cary 100 Bio UV–Vis spectrophotometer with 10 mm quartz cuvettes containing nondegassed solutions of the investigated compounds (100 μM in DMF). Emission spectra and absolute photoluminescence quantum yields (PLQY) were measured using a Hamamatsu Absolute PL Quantum Yield Spectrometer C11347 (Quantaurus QY) equipped with an integrating sphere, under air-equilibrated conditions. An empty quartz Petri dish or quartz test tube was used as a reference. Gels were deposited directly into the quartz Petri dish for analysis, while compounds **A1**–**A4** (5 mg/mL in CHCl_3_) were placed into the quartz test tube.

### 2.2 Fluorescence Microscopy

The real-time ligand exchange reactions were observed via fluorescence microscopy (Axio Observer 7) using a solution of **A1**-**A4** at 5 mg/mL in CHCl_3_ deposited on a covered quartz Petri dish (Hamamatsu) to prevent solvent evaporation. The samples were irradiated at λ = 385 nm (Light Source Colibri 5 Type RGB-UV; Wavelength Range: UV 385 ± 15 nm, blue 469 ± 19 nm, green 555 ± 15 nm, 631 ± 16.5 nm).

### 2.3 SEM Analysis

SEM analysis of gels and aggregates was performed with a Zeiss Sigma HD microscope, equipped with a Schottky FEG source, one detector for backscattered electrons and two detectors for secondary electrons (InLens and Everhart Thornley). Samples were drop-casted onto a silicon wafer.

### 2.4 Single-Crystal X-ray Diffraction (XRD) Experiments

Suitable crystal for XRD analysis of complex **2** were obtained by slow evaporation of chloroform. The crystallographic data for compound **2** were obtained by mounting a well-formed single crystal on a 0.3 nylon cryoloop and transferring it to a Bruker D8 Venture Photon II Bruker diffractometer. The APEX 3 program package^56^ was used to obtain the unit-cell and the geometrical parameters and for the data collection. The raw frame data were processed using SAINT^56^ and SADABS^57^ to obtain the data file of the reflections. The structure was solved using SHELXT^58^ (Intrinsic Phasing method in the APEX 3 program). The refinement of the structure (based on F2 by full-matrix least-squares techniques) was carried out using the SHELXTL-2018/3 program.^58^ The calculated molar mass, density and absorption coefficient include four disordered chloroform molecules per cell which do not appear in the final files because of the refinements carried out with data subjected to SQUEEZE. Crystallographic details are reported in Table S1.

### 2.5 Powder XRD Analysis

Powder X-ray diffraction (XRD) patterns were recorded at CEASC (Analysis Center and Certification Services of Padua University) using a PANanalytical X’Pert^3^ Powder diffractometer equipped with a Cu X-ray tube (setting 40 kV e 40 mA), mirrow BBHD, sample spinner and PIXcel 1D solid state detector. Data were collected in continuous scan mode over a 2θ range of 2.0° to 70.0°, with a step size of 0.013° and a virtual time per step of 200 s (1 h and 10 min per sample). All measurements were performed at room temperature (298 K). Samples were measured as fine powders spread on a flat Si zero-background sample holder.

### 2.6 Thermodynamic Measurements: Binding Affinity Constants

A solution of **A1** (500 μM) in CHCl_3_ was titrated with a 50 mM solution of each ligand (**L2**–**L4**). UV–Vis spectra were recorded after each addition of 0.5 equiv of free ligand. Binding isotherms were obtained by plotting the magnitude of the absorbance change (at 424, 404, 386, 344, and 332 nm) as a function of the concentration of **A1**. Data were fit to a 1:1 binding model with the “bindfit” tool www.supramolecular.org.^59,60^

## 3 Results and Discussion

A series of luminescent Pt­(II) complexes incorporating the well-known 2,6-bis­(3-(trifluoromethyl)-1H-1,2,4-triazol-5-yl)­pyridine as a N̂N̂N terdentate ligand,^61,62^ and various ancillary ligands were synthesized as monomeric units for supramolecular assemblies. Specifically, we employed 4-chloropyridine (**L1**), 1-methylbenzimidazole (**L2**), 1-butylimidazole (**L3**), and 1-methylimidazole (**L4**) to synthesize complexes **1**-**4**, respectively (see Figure 1a). The investigation of different ligand exchange reactions was enabled by selecting ancillary ligands that offer a proven range of thermodynamic stability and kinetic behavior across the complexes. Specifically, pyridine derivatives were combined with the more stable imidazole and benzimidazole derivatives. This diversity facilitates a more comprehensive analysis by NMR, and optical spectroscopy. Our study begins with the characterization of the optical and self-assembly properties of each individual component. We then investigate the ligand exchange reactions, which may proceed via two distinct mechanisms: under homogeneous conditions, through the molecularly dissolved state that exists in dynamic equilibrium with the assembled structures (Figure 1b-i), or under heterogeneous conditions, where the exchange occurs directly within the assembled state of the Pt­(II) complexes (Figure 1b-iii).

**Figure 1:**
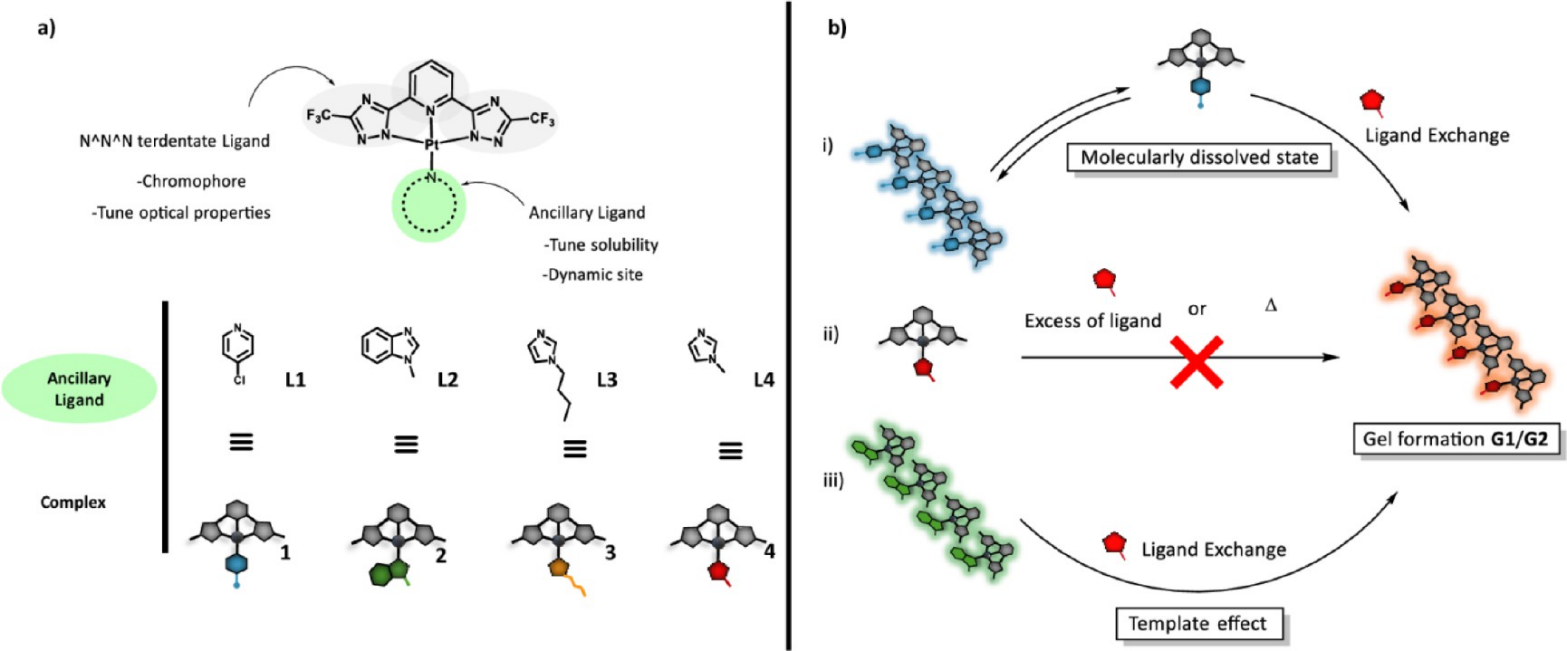
(a) Complexes under investigation with the same skeletal structure but with different ancillary ligand; (b) proposed dynamic ligand exchange pathways enabling access to specific structural motifs.

### 3.1 Photophysical and Morphological Characterization

All complexes were synthesized following established literature procedures,^63^ with slight modifications (Scheme S1), and characterized using standard techniques, including ^1^H and ^13^C NMR spectroscopy, ESI-MS, X-ray powder diffraction and single-crystal when possible. (Figures S1–S20). Notably, the four complexes, in their aggregated forms, exhibit distinct PXRD patterns. These differences highlight the role of the coordinating ligands in directing the aggregation process and provide strong evidence for the structural integrity and phase identity of each discrete assembly. Single-crystal analysis of complex **2** revealed its molecular packing in the aggregated state. The monomers exhibit face-to-face interactions with aligned Pt centers distanced 3.37–3.62 Å one to each other as usual for metallophilic interactions. The 1-methylbenzimidazole moieties alternate within the structure every two units, as more clearly illustrated in Figure S20. In the molecularly dissolved state, the absorption spectra of complexes **1**-**4** display bands in the UV region (310–350 nm), which are attributed to intraligand (^1^IL) transitions and metal-perturbed interligand charge transfer (^1^ILCT) states (Figure S21). At lower energies, a broad absorption feature between 350 and 450 nm is observed, which is assigned to a combination of spin-allowed metal-to-ligand charge transfer (^1^MLCT) and intraligand (^1^IL) transitions: typical behavior for this class of Pt­(II) complexes.^64,65^ The compounds exhibit negligible luminescence in their monomeric form in solution. However, upon increasing the concentration in pure CHCl_3_ to 5 mg/mL (7 mM), all complexes **1**-**4** form their respective emissive aggregates **A1**-**A4**. Complexes were just dispersed into the solvent allowing the formation of aggregates. Repeated cycles of heating (up to 60 °C) followed by cooling to room temperature did not significantly affect the morphology of the resulting aggregates in most cases. An exception was observed for **A1**, which, upon mild thermal treatment, formed longer and more ordered fibers within a few seconds compared to those observed in the initial dispersion. Interestingly, each complex displays a distinct photoluminescence emission spectrum and photoluminescence quantum yield (φ_PL_) upon aggregation (Supporting Information, Table S2). As shown in Figure 2a, aggregate **A1** exhibits a structured blue emission (λ_max_ = 462, 490, and 523 nm, φ_PL_ ≈ 12%), typically associated with aggregates lacking significant metallophilic interactions.^65,66^ In contrast, aggregates **A2**-**A4** show progressively red-shifted and unstructured emissions: green (λ_max_ = 520 nm, φ_PL_ ≈ 7%), yellow (λ_max_ = 580 nm, φ_PL_ ≈ 12%), and red (λ_max_ = 598 nm, φ_PL_ ≈ 4%), respectively, indicating an increasing degree of Pt···Pt interactions (Figure 2b–d). All aggregates are sufficiently macroscopic to be clearly visualized via fluorescence microscopy (Figure 2e–h). **A1** is constituted by elongated fibers exceeding 50 μm in length, while **2** assembles into tubular structures of similar length (aggregate **A2**). Notably, **A3** is constituted by two coexisting morphologies: green-emissive fibers (referred to as **A3a**), which are thinner but comparable in length to those of complex **2**, and orange-emissive heterogeneous aggregates (designated as **A3b**). However, after approximately 2 h, the system evolves toward the exclusive formation of **A3a**, identifying it as the thermodynamically favored aggregated state (λ_max_= 534 nm, φ_PL_ = 34%). Complex **4**, on the other hand, forms small, granular aggregates with distinct morphology (**A4**). Further morphological and structural insights were obtained through scanning electron microscopy (SEM) analysis (Figure 2i–l). The tubular structures observed for compound **2** are, in fact, well-defined assemblies with an astonishing hollow rectangular geometry (Figure 2j). In contrast, the granular aggregates of complex **4** are composed of submicron-sized fibers (<1 μm in diameter), while the needle-like morphologies previously observed for complexes **1** and **3** were confirmed (see Figures S22–S25 for more morphological details).

**Figure 2:**
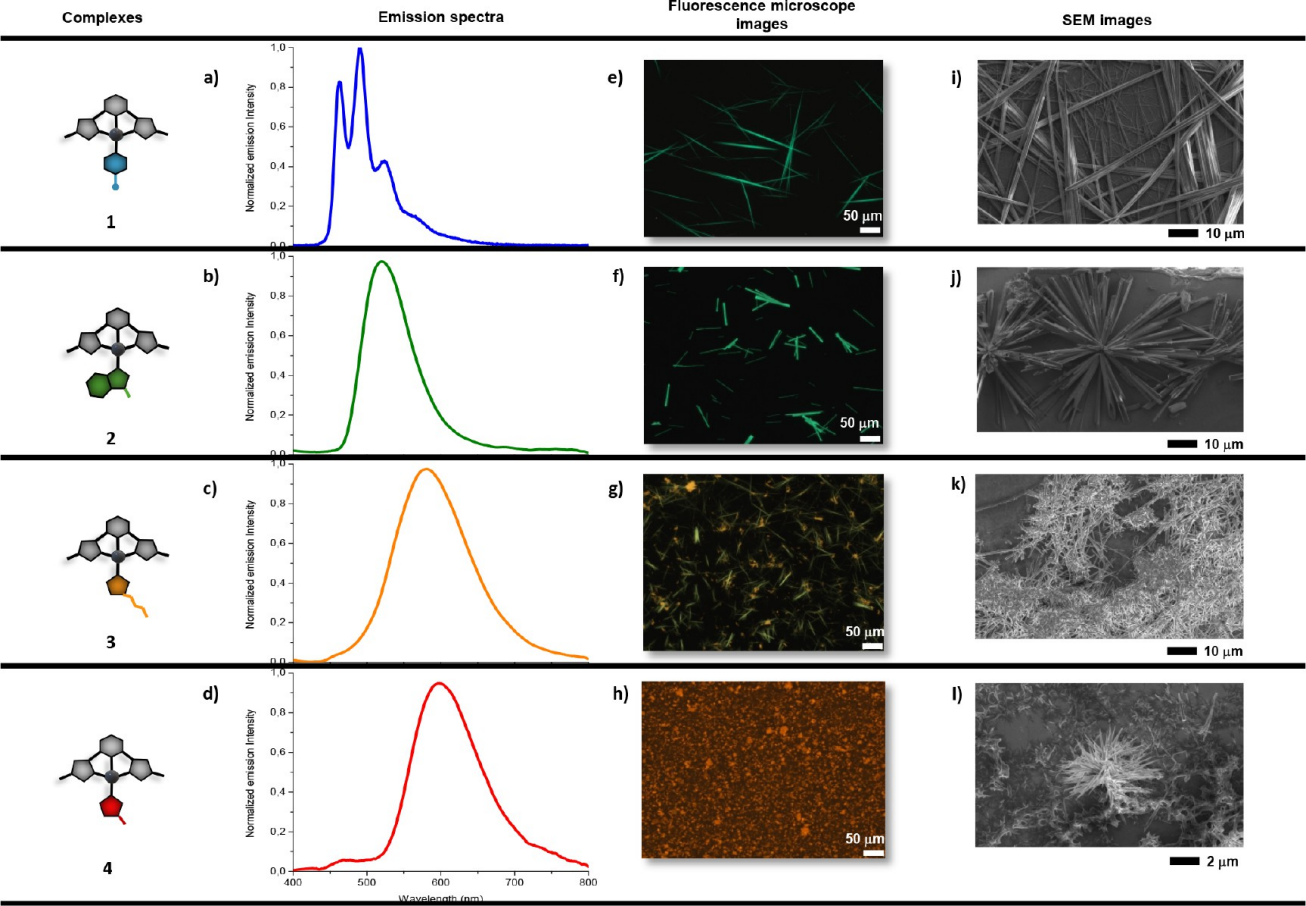
Photophysical and morphological characterization of complexes **1**-**4**. (a–d) Emission spectra of the aggregates [complex] = 5 mg/mL in CHCl_3_. (e–h) Fluorescence microscopy images of the aggregates [complex] = 5 mg/mL in CHCl_3_. (i–l) SEM images of dried aggregates obtained by solution deposition.

### 3.2 Dynamic Exchange Reactions

The designed complexes **1**–**4** feature a strongly bound tridentate ligand alongside a labile monodentate ligand, enabling substitution reactions and dynamic ligand exchange. This adaptive behavior offers valuable potential for the development of supramolecular systems capable of modulating their properties in response to external stimuli or environmental changes. Aggregates **A1**–**A4** were exposed to an environment containing an excess of coordinating species (**L1**-**L4**, 5 equiv). The exchange reactions were performed in CDCl_3_, where the compounds exhibit partial solubility, establishing an equilibrium between the monomeric and aggregated forms. The presence of the monomeric species in solution enables the reaction to be monitored by NMR spectroscopy. (see Figures S26–S33). Notably, we observed that the benzimidazole- and imidazole-based ligands (**L2**-**L4**) readily replace **L1**, as confirmed by the disappearance of peculiar peaks of **1** after the addition of the free ligand **L2**-**L4**. (see Figures S26–S29). Interestingly, **L1** is unable to displace the imidazole-derived ligands (**L2**-**L4**) even when present in large excess (5 equiv). To obtain quantitative information on ligand affinity, binding constants for **L2**–**L4** were derived from UV–Vis spectra recorded by titrating **A1** with an excess of each ligand (Figure S34–S36). Data reported in Table S3 shows the highest binding constant for **L4** of 536 M^–1^, followed by **L3** with 141 M^–1^ and by **L2** with 4 M^–1^.

#### 3.2.1 Ligand Substitution Reaction between Complex 1–3 and L4

##### 3.2.1.1 Exchange: 1+L4 →G1

As previously discussed, pure complex **4** does not fully dissolve in chloroform at the concentration used for NMR analysis (5 mg/mL) and instead forms granular aggregates (Figure 2h). However, when complex **4** is attempted to be generated in situ via ligand exchange from complex **1** in the presence of 1-methylimidazole (**L4**), the resulting aggregate self-assembles into long, emissive fibers that form an intricate network capable of trapping the solvent, ultimately resulting in the macroscopic formation of a gel (**G1**). This gel exhibits the same emission profile as aggregate **A4**, with a slight red shift of the maximum to 605 nm (Figure S37), but it shows a remarkable increase in PLQY, reaching up to 89%. The reversibility of gel **G1** was investigated through heating and cooling cycles aimed at completely dissolving the solid structure and subsequently reforming it. Upon heating, the gel collapses and forms a film-like residue that retains the same photophysical properties as the original gel.

To further examine the conditions required for gelation, a series of synthetic pathways were tested. A dispersion of compound **4** at a concentration of 5 mg/mL in chloroform was heated until complete dissolution of the solid, then cooled to room temperature. This procedure produced a dispersion of aggregates like **A4**; however, gelation did not occur. Additionally, to determine whether the gelation might be influenced by an excess of uncoordinated ligand, complex **4** was treated with an excess of **L4** or **L1** (5 equiv each, 0.06 M) under heating, followed by cooling. Despite these modifications, gelation was not observed, and the properties of the resulting supramolecular aggregate remained consistent with those of **A4**. This set of experiments not only confirms that **G1** is obtainable only by exchange reaction but also shed light on the metastable nature of **G1**. Fluorescence microscopy analysis of **G1** revealed the formation of orange-emissive ribbon and needle-like supramolecular structures, exhibiting unique morphologies that cannot be obtained directly from complex **4** (Figure 1b-ii). The formation of this emissive gel via ligand exchange was successfully replicated and monitored in real time using fluorescence microscopy. A sample of **A1** in chloroform (5 mg/mL) was placed in a Petri dish under the microscope, where the initial blue/green-emissive fibers (**A1**) were clearly visible. Upon the addition of a 1-methylimidazole (**L4**) solution in CHCl_3_ (final concentration 0.06 M), we observed the gradual dissolution of **1**, followed by the emergence of **G1** as bright orange-emissive fibers (Movie S1 and Figure 3 for snapshots). This observation supports a homogeneous mechanism, in which ligand exchange occurs while complex **1** is in the molecularly dissolved state, forming complex **4**, which then undergoes self-assembly into **G1**. In this scenario, **1** acts as a reservoir, continuously supplying dissolved species that maintain a local concentration gradient. This gradient likely plays a key role in promoting the formation of the distinct elongated morphologies observed in **G1**. Once formed, **G1** was dried and washed with acetonitrile and chloroform to remove the excess of ligand. The obtained powder was completely dissolved in DFM-*d*_7_ and characterized by ^1^H NMR spectroscopy (Figure S38). The spectrum revealed the exclusive presence of complex **4**, with no detectable signals from the starting material.

**Figure 3:**
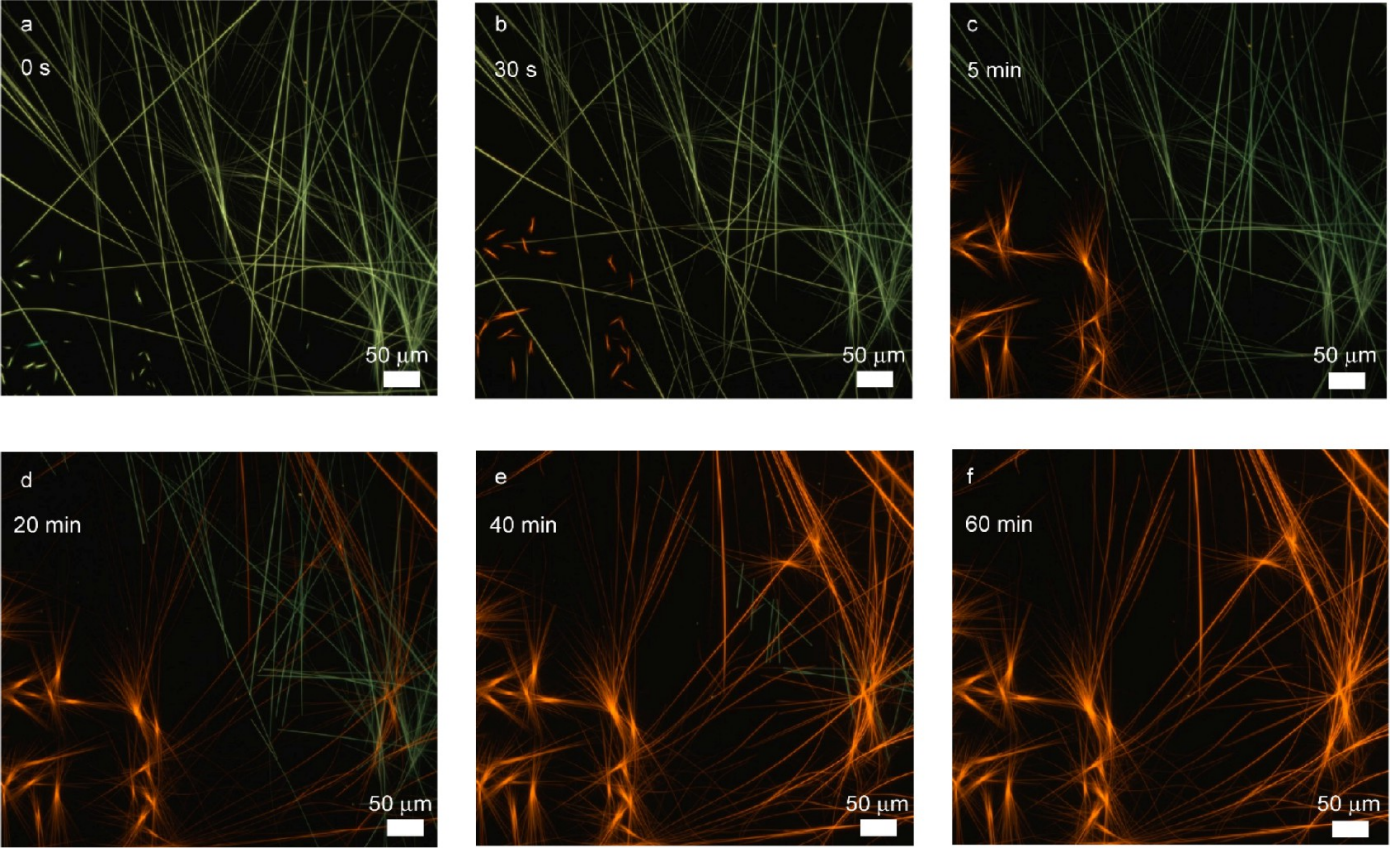
Snapshot of Movie S1. (a–f) Real-time ligand exchange of **A1** with 5 equiv of free **L4** monitored by fluorescence microscope for 60 min.

##### 3.2.1.2 Exchange: 2+L4 →G2

We investigated whether complexes **2** could also give rise to new supramolecular structures under ligand exchange conditions. For this purpose, complex **2**, dissolved in CHCl_3_ at the same concentration 5 mg/mL (forming **A2**), and was treated with an excess of 1-methylimidazole (**L4**). As in previous cases, **L4** rapidly replaced 1-methylbenzimidazole (**L2**) in the coordination sphere, as confirmed by the appearance of a signal at 7.8 ppm in the ^1^H NMR spectrum, corresponding to free **L2** (Figure S31). This ligand exchange also led to the formation of a new gel, **G2**, which exhibits the same emission profile as **G1** (Figure S39), albeit with a significantly lower photoluminescence quantum yield (φ_PL_ = 57%). Fluorescence microscopy analysis of **G2** (Figure S40) revealed structural features reminiscent of the starting material, **A2**. Real-time microscopy further confirmed that upon the addition of **L4** to **A2**, not only do new aggregates of complex **4** form under homogeneous mechanism, but **A2** also appears to undergo a direct transformation into **A4**. This is evidenced by the partial retention of **A2**’s original morphology during the exchange process (Movie S2, Figure 4 for snapshots) suggesting that the ligand substitution may occur within the preassembled state, preserving some supramolecular features of the starting material. However, the ligand exchange reaction also proceeds via a homogeneous pathway involving the monomeric species dissolved in solution. This dual mechanism is supported by the observation of new material forming around **A2** aggregates, as well as by the conversion of **A2** into **A4**, ultimately leading to the formation of **G2**. The coexistence of these pathways explains the presence of two types of structures observed by fluorescence microscopy, which differ notably from the more uniform morphology of **G1.**

**Figure 4:**
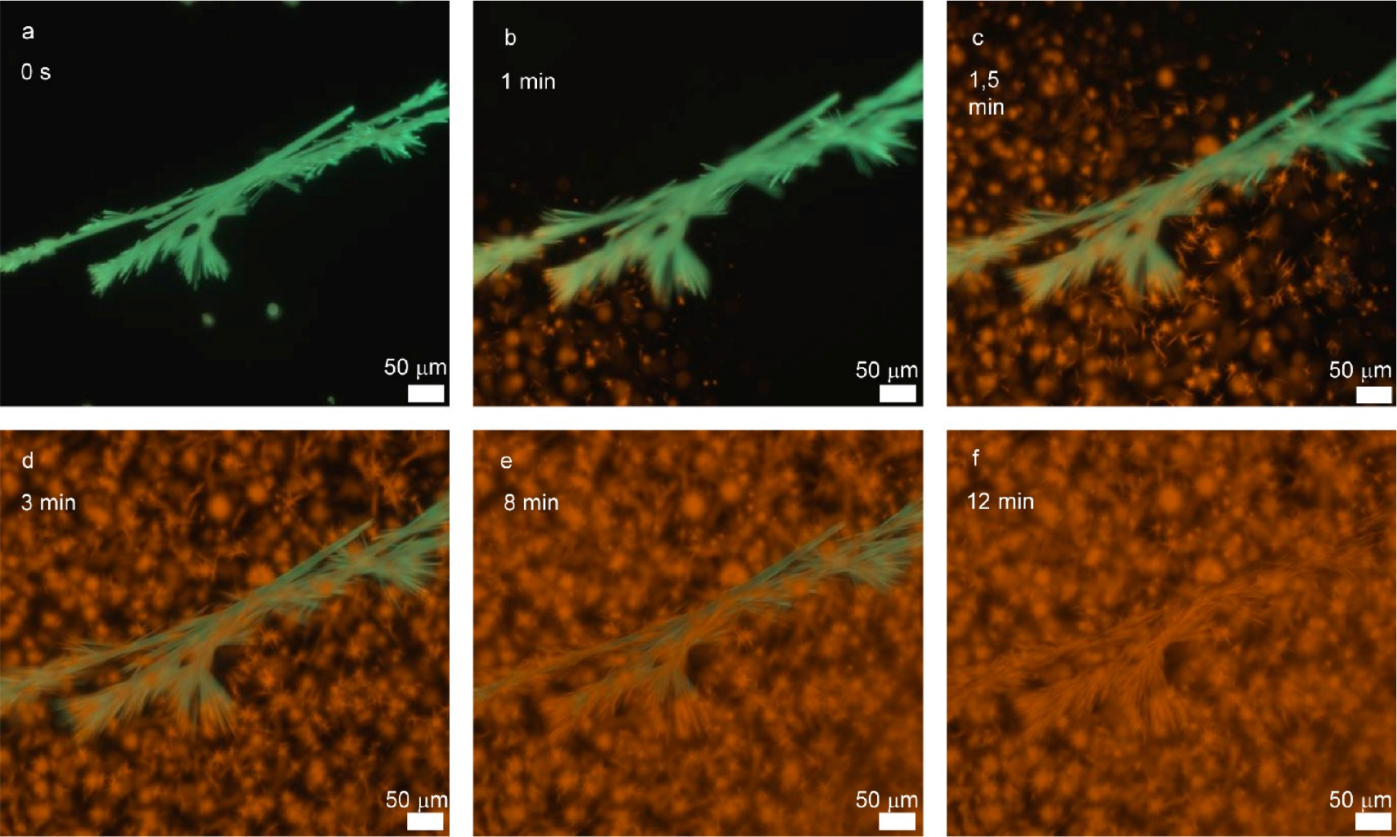
Snapshot of Movie S2. (a–f) Real time ligand exchange of **A2** with 5 equiv of free **L4** monitored by fluorescence microscope for 12 min.

While the fluorescence images reveal this heterogeneity, SEM analysis confirms that both **G1** and **G2** share a fibrous architecture at the nanoscale (Figures S41 and S42). Figure S42 shows that in **G2** the fibers are partially aligned in certain regions, likely due to a templating effect from the starting material. The uniform morphology of the fibers confirms that the original starting material did not persist after interaction with the incoming ligand. However, differences in the internal supramolecular organization between **G1** and **G2** are also evident from their PXRD patterns. **G1** and **G2** exhibit similar powder XRD patterns (Figure S43), with some peaks in **G2** slightly shifted toward lower 2θ values, indicating variations in internal packing or crystallinity. These shifts likely reflect differences in Pt···Pt distances, which may induce structural defects that influence the PLQY. Moreover, the two patterns differ markedly from that of **A4** (Figure S17), providing further evidence that structural transformations play a key role in determining the final PLQY.

We then investigated the dynamic ligand exchange between imidazole units. Figure 5 summarizes all the reactions conducted.

**Figure 5:**
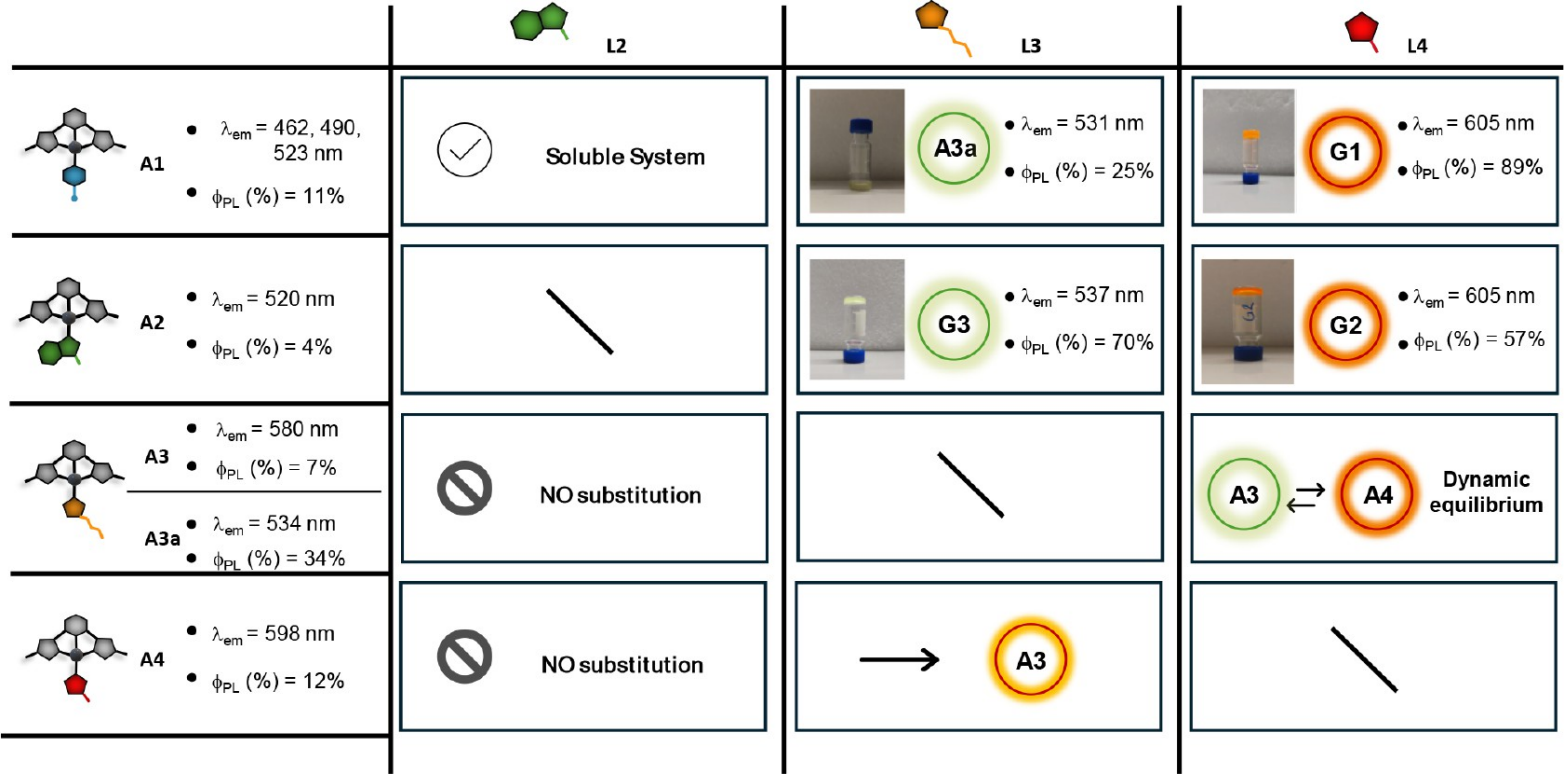
Summary of all ligand-exchange reaction conducted.

##### 3.2.1.3 Exchange: 3+L4 →A3 ⇌ A4

To a solution of complex **3**, which contains 1-butylimidazole (**L3**) as the ancillary ligand, 5 equiv of 1-methylimidazole (**L4**) were added in an NMR tube in CDCl_3_. Initial ^1^H NMR analysis (Figure S32) revealed the formation of complex **4**, as indicated by the appearance of a new singlet at 8.72 ppm after 2.5 h, corresponding to the imidazolic proton (N–CH–N) of the **L4**-bound species. Simultaneously, the singlet at 8.85 ppm, attributed to complex **3**, began to decrease. After 6 h, approximately 50% of complex **3** had been converted into **4**. However, over time, the system exhibited a notable reversal: the signal at 8.85 ppm (complex **3**) began to increase again, while the peak at 8.72 ppm decreased and gradually shifted to 8.78 ppm, suggesting a change in the coordination environment, likely due to supramolecular aggregation. After 24 h, an equilibrium was established between the two platinum complexes with approximatively 40% of conversion into complex **4**. These observations point to a reversible and dynamic ligand exchange process between **L3** and **L4**, governed by an interplay between intrinsic ligand affinity and their aggregation behavior. Initially, the substitution of **L3** with the more basic and smaller 1-methylimidazole (**L4**) appears to be kinetically favored, forming complex **4**. Yet, as the reaction progresses, thermodynamic factors, such as solubility and supramolecular stabilization, begin to dominate. The partial reversion back to complex **3** suggests that its aggregation state may stabilize the system, potentially acting as a reservoir for the **L4**-bound species shifting the equilibrium back. This is further supported by fluorescence microscopy, which revealed the coexistence of two distinct types of aggregates within the same sample (Figure S44). However, in the absence of thermodynamic data on the aggregation process, we are unable to fully rationalize how aggregation free energies compete with ligand affinities in determining the system’s reversibility. *Exchange: 4 + L3 → A3* Instead, when complex **4** was placed in an environment rich in 1-butylimidazole (**L3**), the exchange reaction proceeded to completion, as confirmed from time-resolved ^1^H NMR spectra (Figure S33) that shows the only presence of complex **3** after 24 h. Fluorescence microscope reveals the formation of two different aggregates related to complex **3** (Figure S45).

##### 3.2.1.4 Exchange: 1+L2→Soluble System

Interestingly, **L2** (1-methylbenzimidazole) was able to substitute only **L1** in the coordination sphere but was unable to displace either **L3** or **L4**, even after 24 h, highlighting a clear hierarchy in ligand binding affinity. When complex **1** was exposed to an excess of **L2**, the ligand exchange occurred efficiently (Figure S26); however, upon completion of the substitution, the resulting system became fully soluble in CHCl_3_, and no emissive aggregates were observed.

#### 3.2.2 Exchange 1+L3 →A3a

3.2.2.1

In contrast, when 1-butylimidazole (**L3**) was used under the same conditions, ligand exchange also proceeded successfully (Figure S27), this time leading to the formation of dense green-emissive fibers, although without any gelation. These fibers exhibited a PLQY of 25%, with a structured emission profile characterized by a main maximum at 531 nm and two additional relative maxima at 464 and 494 nm consistent with the formation of **A3a** (Figure S46).

##### 3.2.2.2 Exchange 2+L3→G3

A gelation process was instead observed upon substitution of 1-methylbenzimidazole (**L2**) in complex **2** with 1-butylimidazole (**L3**) (Figure S30), resulting in a network of green-emissive fibers (**G3**) with a PLQY of 70% and a maximum emission at 537 nm (Figure S47). In this case, the emission spectrum of **G3** was slightly blue-shifted relative to that of **A3a** and exhibited a slight shoulder at 494 nm. Fluorescence microscopy analysis of **G3** revealed a compact and homogeneous network of green fibers, with no apparent morphological heterogeneity that could account for the spectral features (Figure S48). SEM analysis confirmed the presence of long, well-defined fibers forming the structural skeleton of the gel (Figures S49 and S50). However, these fibers exhibited a square-like morphology, distinct from those formed in the previous ligand-exchange reaction (**1**+**L3** →**A3a**) that formed curved and flatter fibers (Figure S51). In both cases, the resulting structures resemble the morphology of their respective starting materials, **A1** and **A2** in the exclusive formation of **A3a** with different PLQYs.

#### 3.2.3 Competitive Multicomponent Substitution with L2–4 from Complex 1

The complexity observed in the self-assembly pathways under ligand exchange conditions prone us to investigate a competitive multicomponent reaction. Complex **1** was placed in a solution containing an excess of free **L2**-**L4** (5 eq. each), the initial aggregate dissociated and reassociated, forming different structures depending on the ancillary ligand that successfully coordinated the metal center. This competitive equilibrium was monitored using ^1^H NMR and fluorescence microscopy, which provided complementary information. The initial blue-emissive fibers of **A1** were rapidly replaced by long, densely packed green fibers, which were identified as complex **3**, also in agreement with NMR data. Notably, as previously hypothesized, **A1** appears to act as a template or nucleation center for the elongation of **A3** fibers, as the system in Figure 6b retains structural features of the starting materials Figure 6a. After 30 min, orange-emissive fibers began to appear, showing that **L4** replaced **L3**. Indeed, from the NMR spectra we can observe how the characteristic peak of complex **4** decreased for its progressive formation and precipitation. After 24 h, the green emissive network completely evolves into the orange-emissive fibers of complex **4** (Figure 6f). The entire process is also shown in Movie S3. This trend is consistent with the calculated binding constant. However, ^1^H NMR spectra (Figure S52) show that, while the orange-emissive fibers of **A4** appear, complex **3** remains dissolved in solution. The equilibrium previously observed between complex **3** and complex **4** is thus maintained even in these conditions after 24 h.

**Figure 6:**
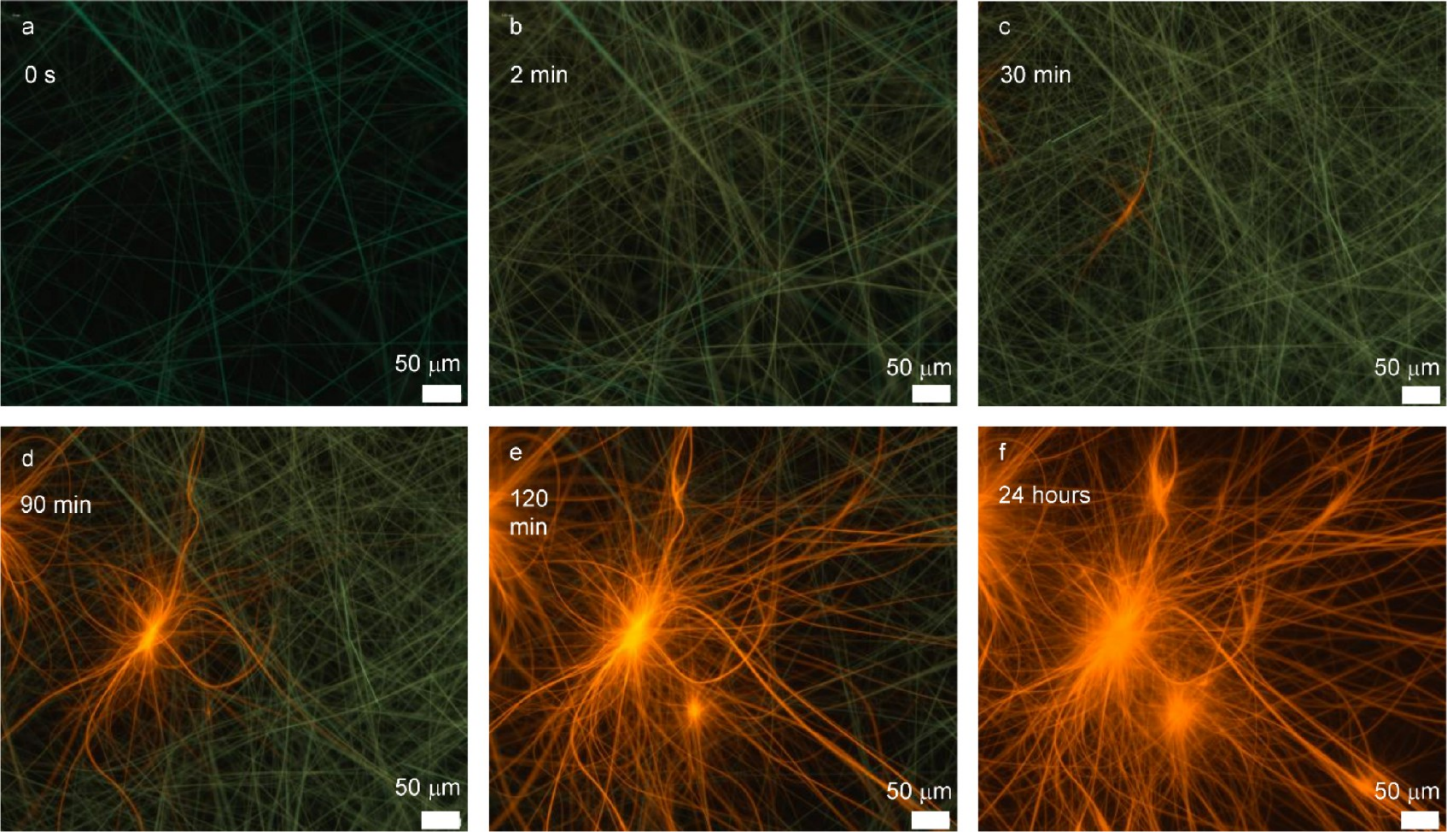
Snapshot of Movie S3. (a–f) Real time multiligand exchange of **A1** with an excess of **L2**-**4** monitored by fluorescence microscope for 24 h.

## 4 Conclusions and Outlook

This study demonstrates a novel, sequential approach to supramolecular design that bridges coordination-driven and hierarchical self-assembly. By leveraging the dynamic ligand exchange behavior of luminescent Pt­(II) complexes bearing a chromophoric N̂N̂N terdentate ligand and various ancillary donors, we show that the formation of distinct supramolecular architectures can be directed and modulated in solution through controlled substitution reactions. Notably, the in situ formation of emissive gels (**G1** and **G2**) via ligand exchange reveals how metastable supramolecular states can emerge under nonequilibrium conditions, inaccessible through direct synthesis. This transformation, monitored in real-time by fluorescence microscopy, further illustrates the dual role of initial aggregates as both precursors and templates for new assemblies. Moreover, the observed reversibility and competitive behavior of imidazole-based ligands underscore the intricate balance between kinetic and thermodynamic factors in dictating final aggregate morphology and composition.

Overall, these findings provide a compelling framework for the development of adaptive, reconfigurable materials via stepwise, noncovalent synthesis. By coupling self-assembly with metal-mediated ligand exchange, our approach opens up new possibilities for designing responsive nanostructures with tailored properties, representing a step toward adaptive, life-like materials with potential applications in sensing and optoelectronics.

## Supplementary Material









## Notes

Gradual dissolution of **1**, followed by the emergence of **G1** (Movie S1) (AVI)
Direct transformation of **A2** into **A4** with partial morphology retention (Movie S2) (AVI)
Multiligand exchange of **A1** (Movie S3) (AVI)
^1^H and ^13^C NMR spectra for all compounds and for dynamic ligand exchange reactions, crystallographic data, SEM images and fluorescence microscope images, and thermodynamic data and additional experimental details (PDF)

## Notes

Crystallographic data have been deposited with the Cambridge Crystallographic Data Centre as supplementary publication. CCDC Deposition Number 2449187. Copies of the data can be obtained free of charge on application to the CCDC, 12 Union Road, Cambridge CB2 1EZ, U.K. (fax, (+44) 1223 336033; e-mail, deposit@ccdc.cam.ac.uk).
