# Supplementary material for: Dynamic Reconfiguration of Pt(II) Supramolecular Assemblies via Ligand Exchange
Source: ACS Appl Mater Interfaces. 2025 Sep 11;17(40):56379–88. doi: 10.1021/acsami.5c10381 (PMC12516690; doi:10.1021/acsami.5c10381)
Supplement: Supplementary file 4 [file am5c10381_si_004.pdf]

Supporting Information

Dynamic Reconfiguration of Pt(II) Supramolecular Assemblies via Ligand Exchange

Elisa Pelorosso[1], Mirco Scaccaglia[1], Dario Alessi[1], Claudia Graiff[2], Alessandro Aliprandi[1*]

[1] Dipartimento di Scienze Chimiche, Università degli Studi di Padova; Via Marzolo 1, Padova, 35131, Italy.

[2] Department of Chemistry, Life Sciences and Environmental Sustainability, Università degli Studi di Parma, Parco Area delle Scienze 17/A, 43124 Parma, Italy.

* alessandro.aliprandi@unipd.it

Supplementary Methods 2

General 2

Experimental Procedure 2

Supplementary text 5

Compound Characterization 5

Properties of aggregates 20

Ligand exchange processes 24

Exchange Product analysis 29

References 43

Supplementary Methods

General

All the reactions were carried out under an inert atmosphere of argon. All the solvents and chemicals are used as received from Aldrich or Fluka without any further purification. ^{1}H, and $^{13}C\{^{1}H\}$ NMR spectra were recorded on a Bruker AVANCE III spectrometer equipped with a BBO probe. The ^{1}H and ^{13}C NMR chemical shifts (δ) are given in ppm and referred to residual protons on the corresponding deuterated solvent. All coupling constants (J) are given in Hertz (Hz). All deuterated solvents are used as received without any further purification. HR-MS spectra were recorded by Agilent 6550 iFunnel Q-TOF in positive mode.
All reactions were monitored by thin-layer chromatography using Merck silica gel plates 60 F254 (aluminum sheets) and spots were visualized with UV.
Flash chromatography was performed on a Combiflash Nextgen 300+ using Redisep Bronze column (4, 12, 24, 40 grams) filled with NP silica gel and using Redisep Rf cartridge (4 and 24 grams).
The precursor $[PtCl_2(DMSO)_2]$ and the N^N^N terdentate ligand were synthetized as reported elsewhere. [1]

Experimental Procedure

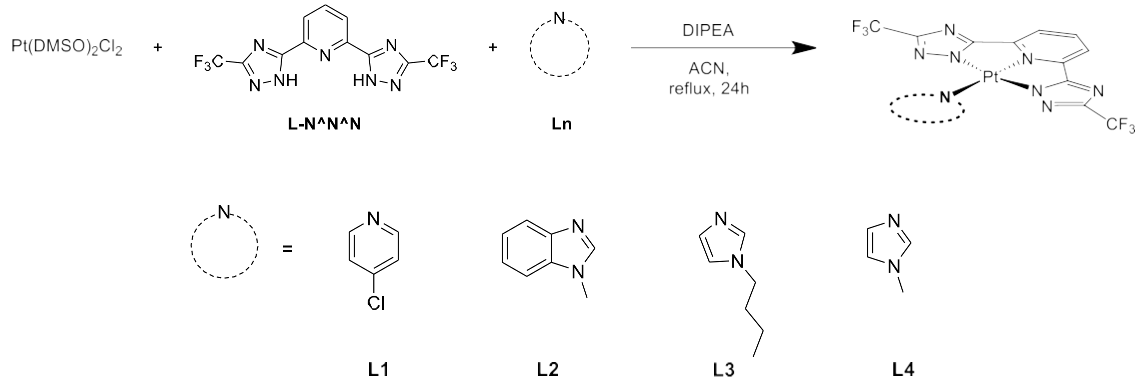

Scheme S1. Synthetic pathway of structures under investigation

All complexes were synthetized via a slightly modified procedure known in literature.[1]
$[PtCl_2(DMSO)_2]$ (100 mg, 0.24 mmol 1 eq.), L-N^N^N (82 mg, 0.24 mmol, 1 eq) and DIPEA (82 mL, 0,48 mmol, 2 eq.) are dissolved in 5 mL of dry acetonitrile under argon atmosphere at room temperature. The solution immediately turned orange is let to stir for almost 10 minutes, then 1 eq. of the ancillary ligand Ln is added to the solution equipped with refrigerator and let it stir overnight under reflux.
After 12 hours, a precipitate is obtained, it is filtrated and washed with acetonitrile, n-hexane and diethyl ether and subsequently structurally characterized by NMR spectroscopy.

Synthesis of complex 1

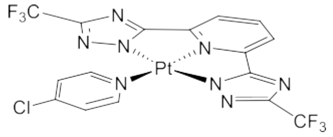

The synthesis of complex 1 was carried out using a general procedure with slight modifications. In this case, 3 equivalents of DIPEA (123 mL, 0.72 mmol) were used instead of 2 equivalents to favor the deprotonation of 4-chloropyridine, which served as the ancillary ligand. The resulting precipitate was purified by flash chromatography, using a gradient of 100% n-hexane to 50% n-hexane/50% ethyl acetate, yielding a yellow/green fluffy solid with a 38% yield (59 mg, 0.09 mmol)

^{1}H NMR (400 MHz, Chloroform-d) δ 9.69 (d, J = 7.0 Hz, 1H), 8.17 – 8.02 (m, 1H), 7.84 (d, J = 7.9 Hz, 2H), 7.66 (d, J = 7.0 Hz, 1H).

^{13}C{^{1}H} NMR (75 MHz, DMF-d_7) δ 164.71, 154.54, 148.57, 148.40, 145.16, 127.70, 121.76, 119.44, 34.77, 29.68.

HR-MS (m/z) Calculated: 656.0114 ([M+H]+); Found: 656.0127 ([M+H]+). Mass deviation: 1.98 ppm.

Synthesis of complex 2

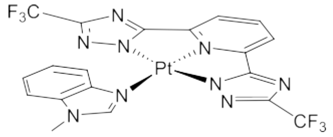

The synthesis of complex 2 was carried out using a general procedure, using 1-methylbenzimidazole as ancillary ligand. It was obtained a lime/green powder with an isolated yield of 41% (65 mg, 0.097 mmol).

^{1}H NMR (400 MHz, Chloroform-d) δ 8.67 (s, 1H), 8.13 – 7.97 (m, 3H), 7.80 (d, J = 7.9 Hz, 3H), 7.61 – 7.40 (m, 4H), 3.85 (s, 3H).

^{13}C {^{1}H} NMR (75 MHz, $CDCl_3$) δ 164.27, 149.18, 147.04, 142.73, 140.02, 132.86, 125.71, 124.71, 120.21, 118.55, 110.30, 32.04.

HR-MS (m/z) Calculated: 675.0768 ([M+H]+); Found: 675.0830 ([M+H]+). Mass deviation: 9.18 ppm.

Suitable single crystals were obtained by slow evaporation of chloroform.

Synthesis of complex 3

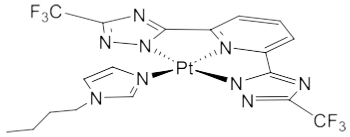

The synthesis of complex 3 was carried out using a general procedure, using 1-butylimidazole as ancillary ligand. It was obtained a yellow powder with an isolated yield of 58% (91 mg, 0.137 mmol).

^{1}H NMR (400 MHz, Chloroform-d) δ 8.85 (s, 1H), 8.13 (s, 1H), 7.95 (t, J = 7.8 Hz, 1H), 7.70 (d, J = 7.8 Hz, 2H), 6.96 (s, 1H), 4.12 (t, J = 7.3 Hz, 2H), 1.90 (p, J = 7.4 Hz, 2H), 1.45 (q, J = 7.5 Hz, 2H), 1.02 (t, J = 7.4 Hz, 3H).

^{13}C {^{1}H} NMR (75 MHz, $CDCl_3$) δ 164.11, 153.16, 148.96, 142.39, 140.28, 130.91, 120.03, 118.26.

HR-MS (m/z) Calculated: 667.1081 ([M+H]+); Found: 667.1109 ([M+H]+). Mass deviation: 4.20 ppm.

Synthesis of complex 4

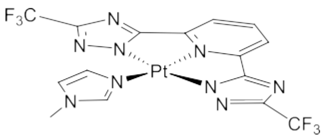

The synthesis of complex 4 was carried out using a general procedure, using 1-methyimidazole as an ancillary ligand. It was obtained a dark orange powder with an isolated yield of 35% (51.7 mg, 0.08 mmol).

^{1}H NMR (400 MHz, Chloroform-d) δ 8.86 (s, 1H), 8.21 (d, J = 1.7 Hz, 1H), 7.97 (t, J = 7.9 Hz, 1H), 7.75 (d, J = 7.9 Hz, 2H), 6.97 (s, 0H), 3.92 (s, 3H).

^{13}C {^{1}H} NMR (75 MHz, DMF-d7) δ 164.82, 148.32, 144.03, 141.17, 129.69, 122.89, 121.97, 119.30, 119.20, 34.89, 29.79.

HR-MS (m/z) Calculated: 625.0611 ([M+H]+); Found: 625.0650 ([M+H]+). Mass deviation: 6.24 ppm.

Supplementary text

Compound Characterization

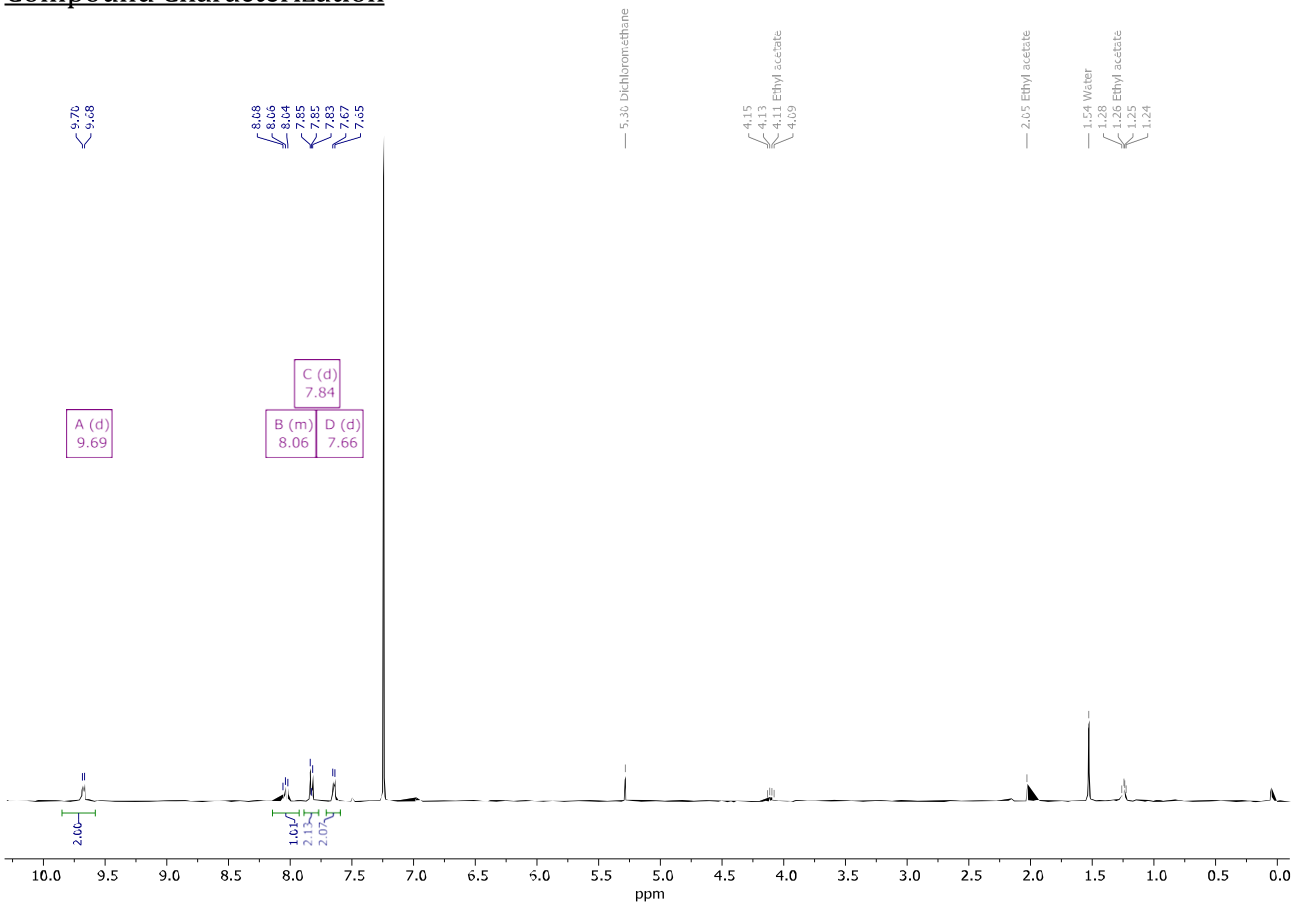

Figure S1. ^{1}H NMR spectrum (400 MHz) of complex **1** in $CDCl_3$ at 25°C.

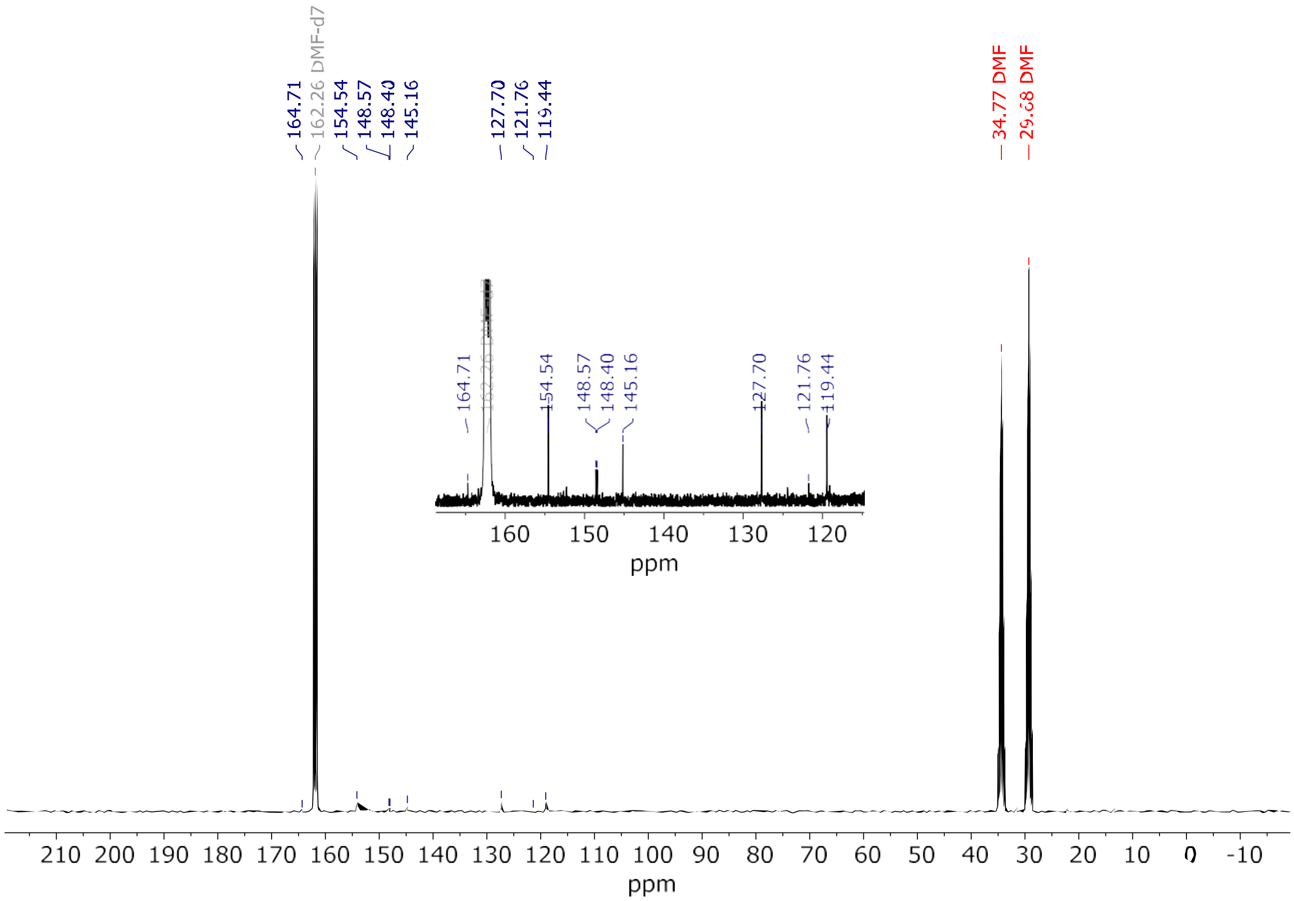

Figure S2. $^{13}C\{^{1}H\}$ NMR spectrum (75 MHz) of **1** in DMF-d$_7$ at 25 °C

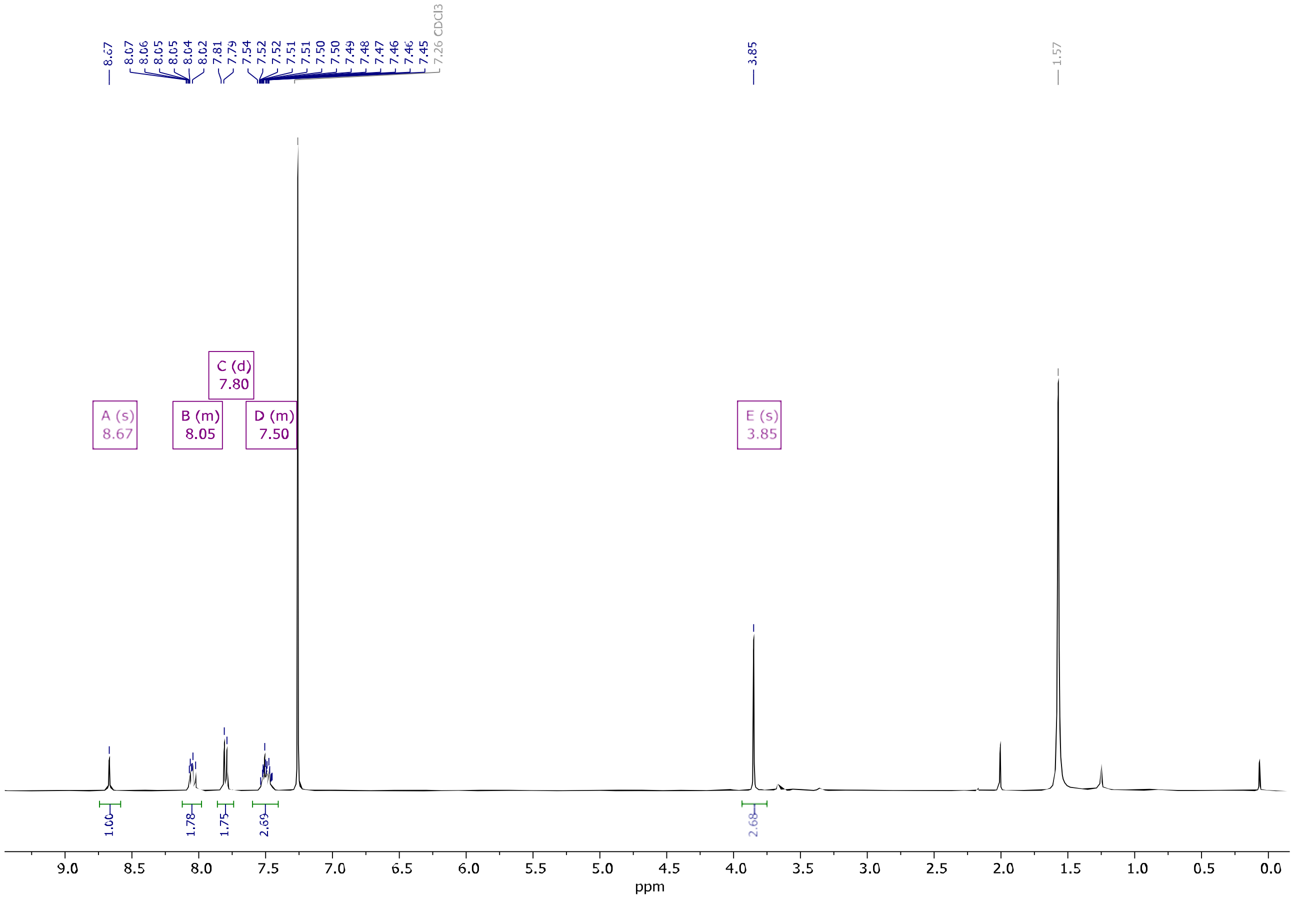

Figure S3. ^{1}H NMR spectrum (400 MHz) of complex **2** in $CDCl_3$ at 25°C.

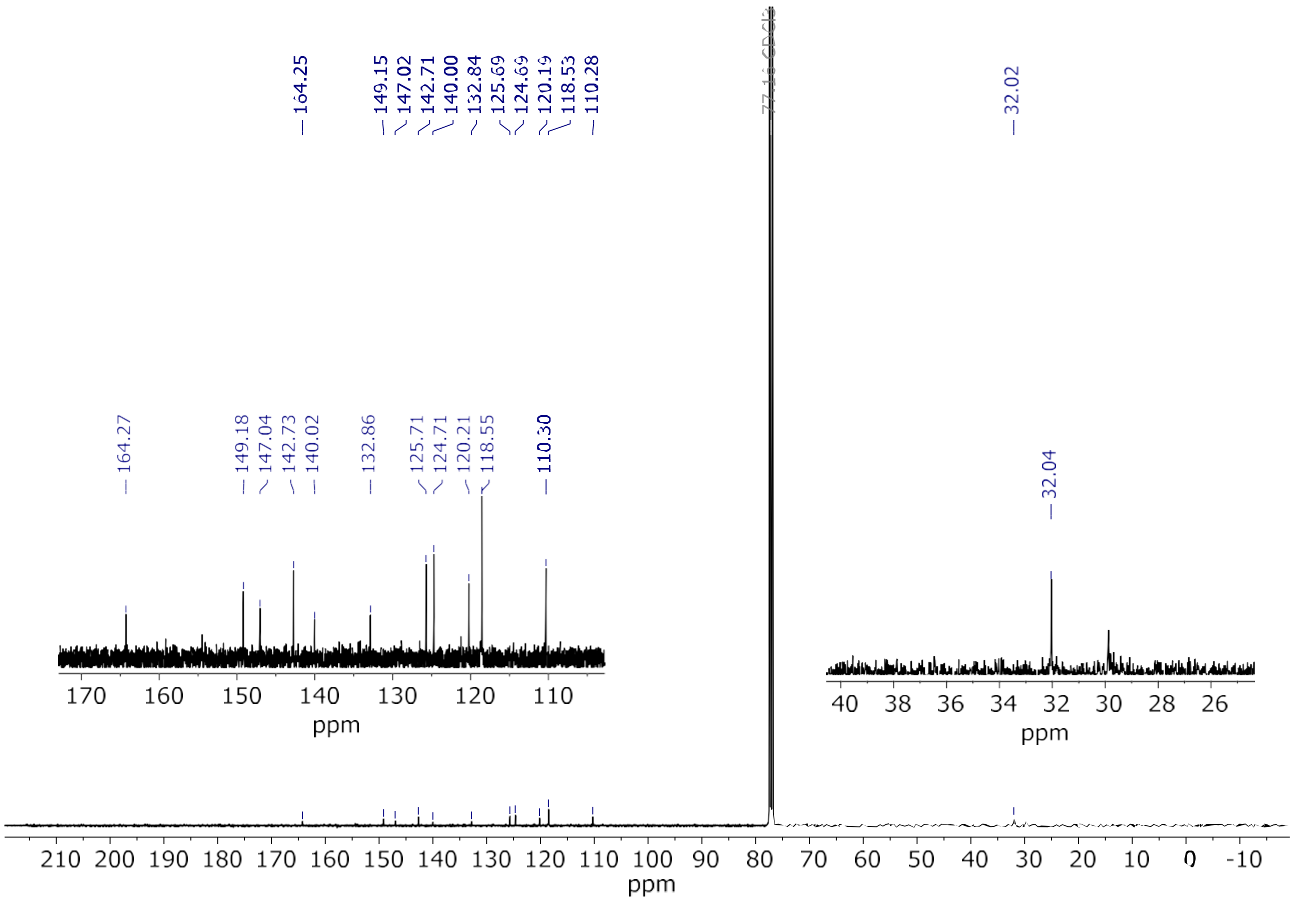

Figure S4. $^{13}C\{^{1}H\}$ NMR spectrum (75 MHz) of **2** in $CDCl_3$ at 25 °C

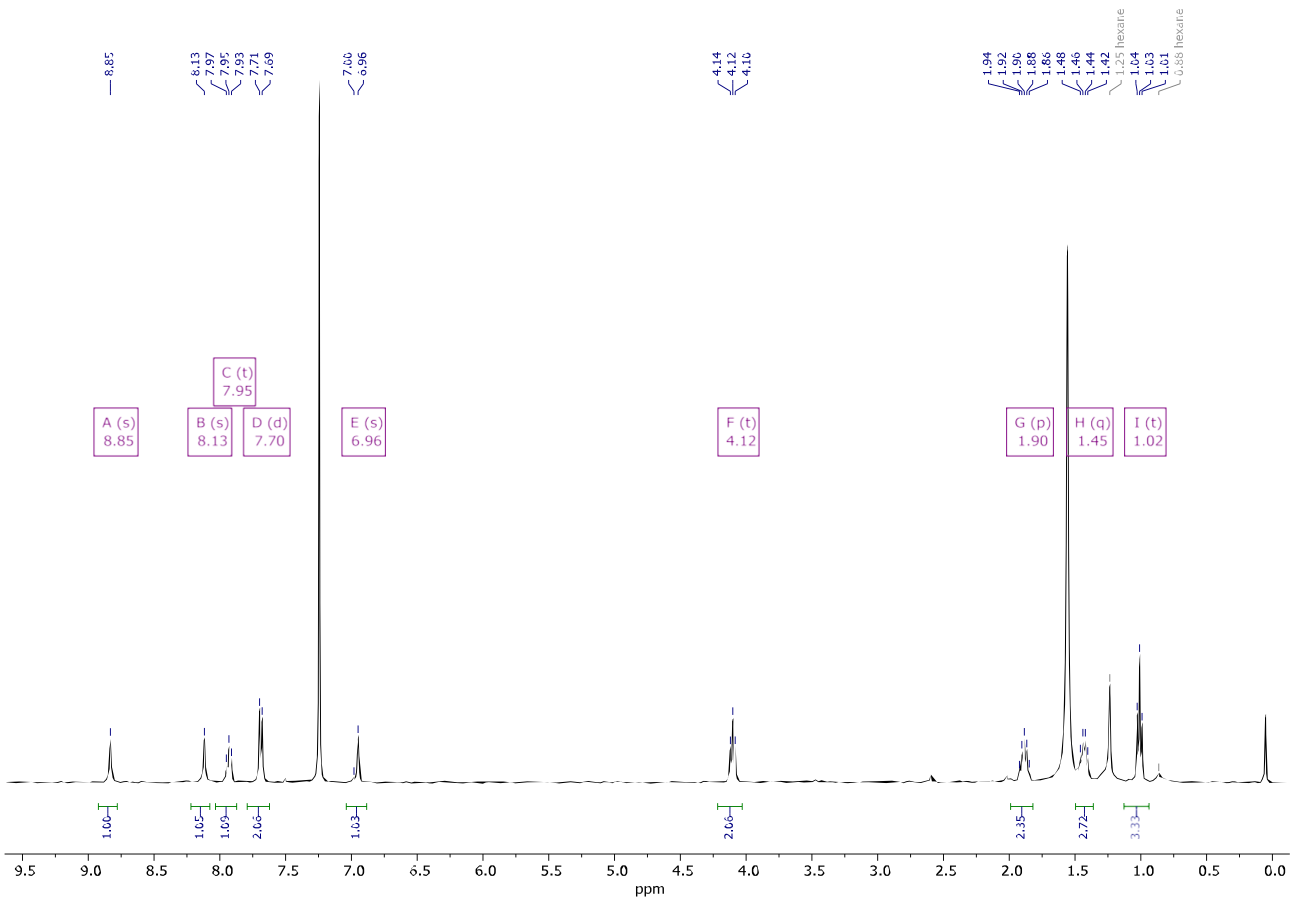

Figure S5. ^{1}H NMR spectrum (400 MHz) of complex **3** in $CDCl_3$ at 25°C.

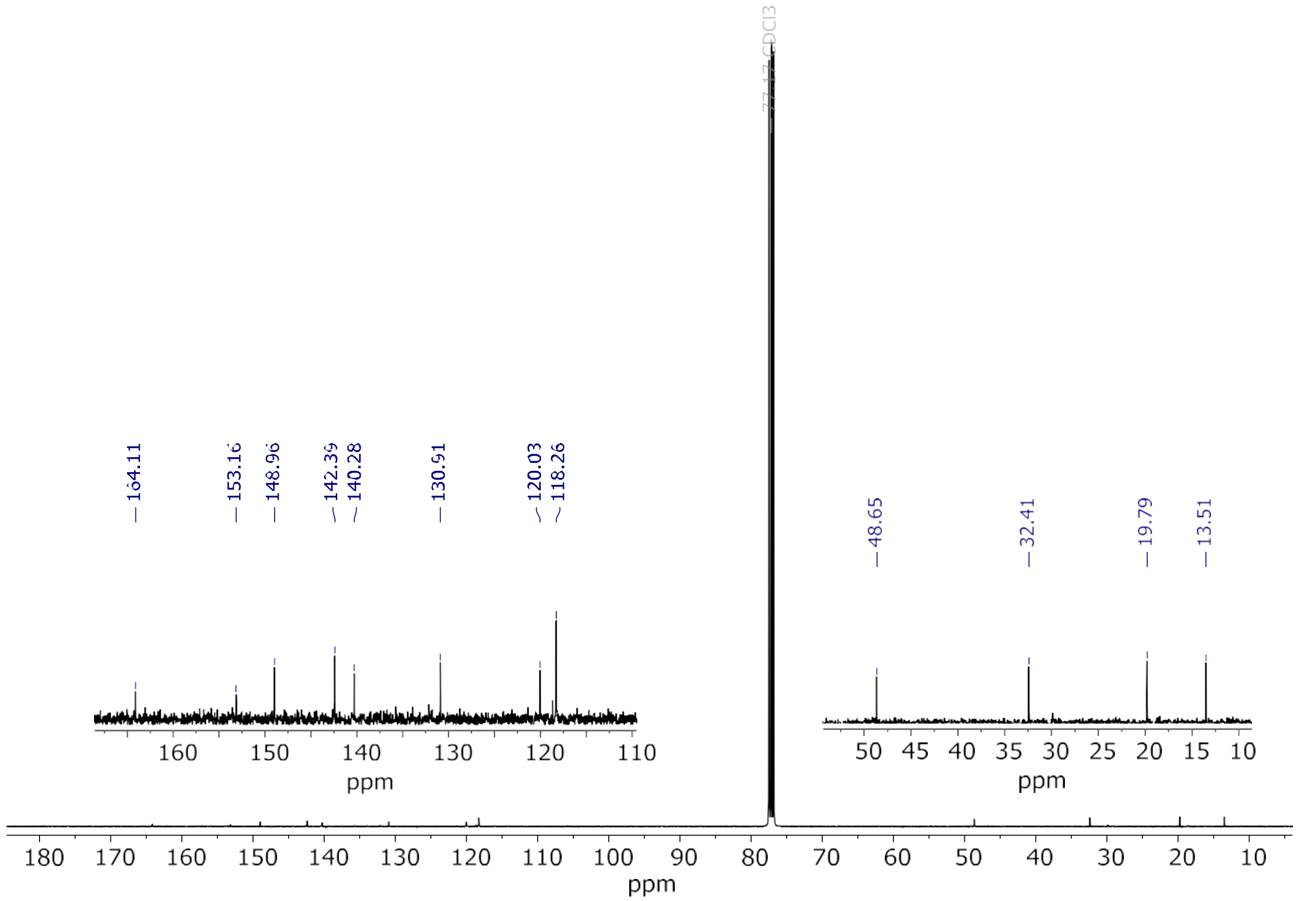

Figure S6. $^{13}C\{^{1}H\}$ NMR spectrum (75 MHz) of **3** in $CDCl_3$ at 25 °C

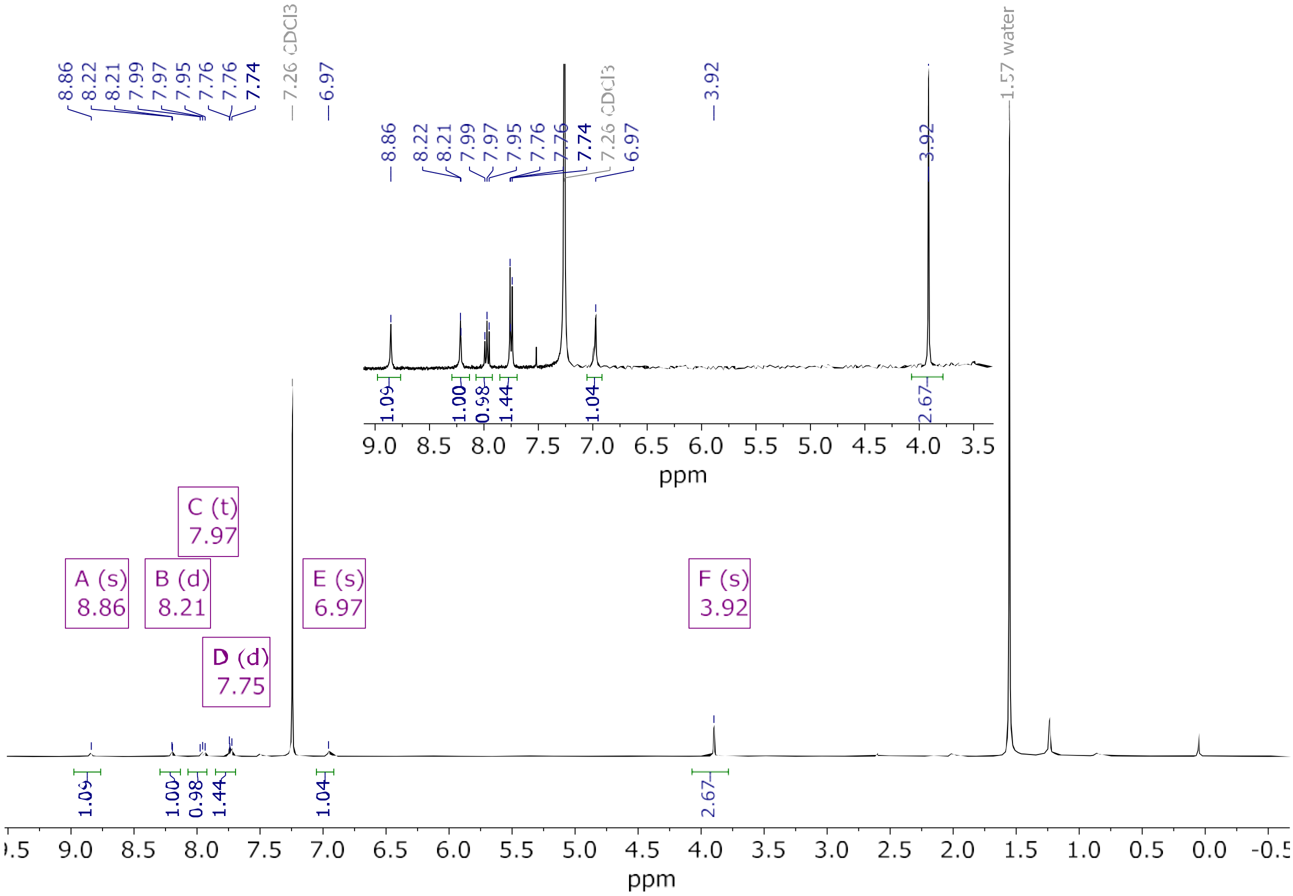

Figure S7. ^1H NMR spectrum (400 MHz) of complex **4** in $CDCl_3$ at 25°C.

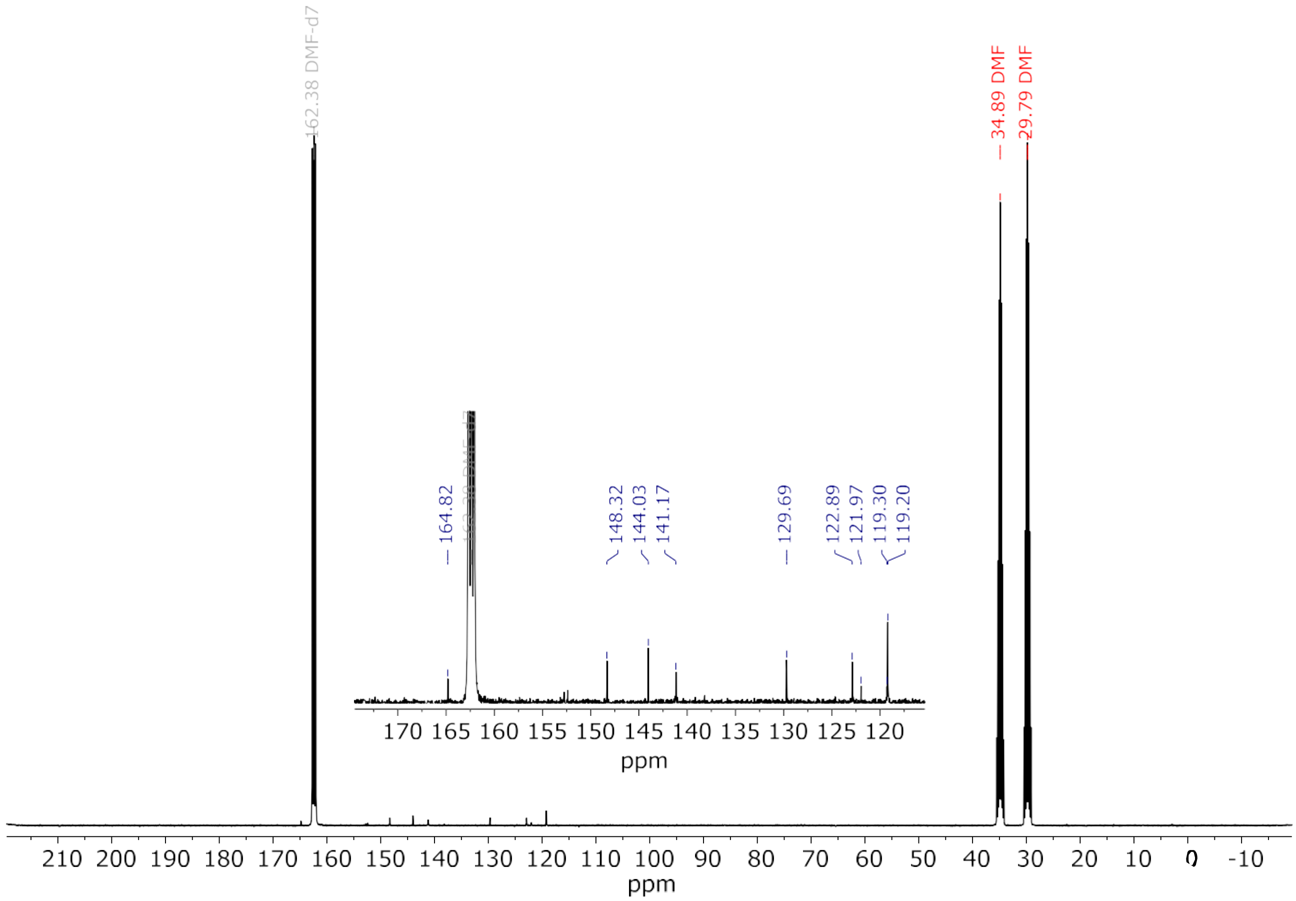

Figure S8. $^{13}C\{^1H\}$ NMR spectrum (75 MHz) of **4** in DMF-d_7 at 25 °C.

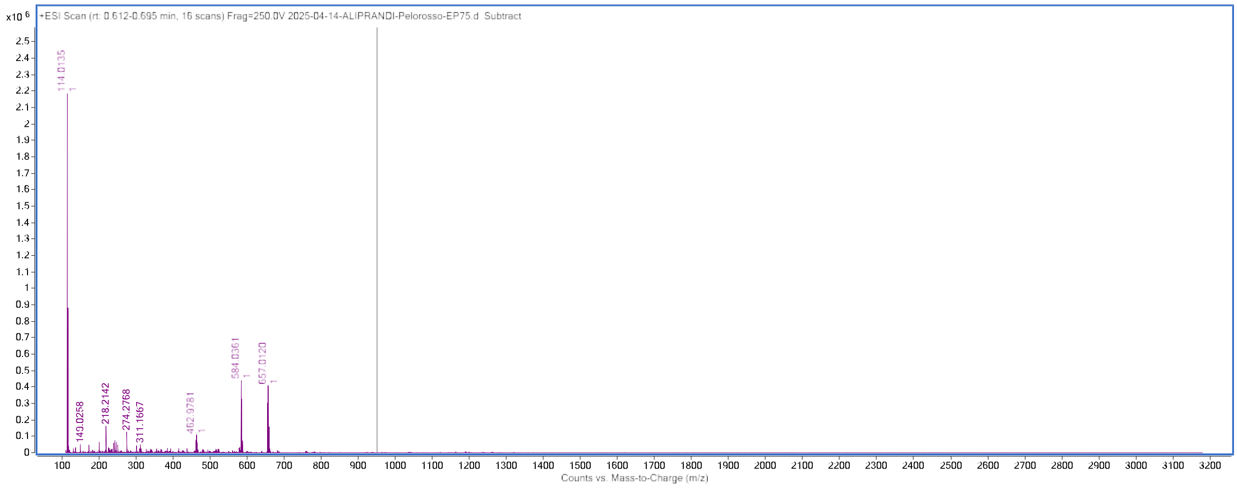

Figure S9. HR-MS ESI scan of complex **1** in positive mode.

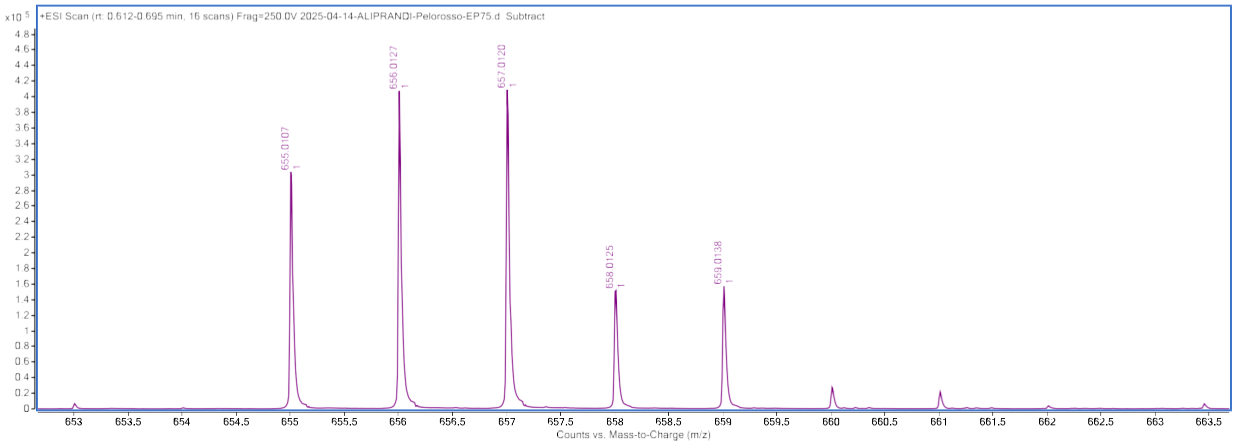

Figure S10. HR-MS ESI scan of complex **1** in positive mode.

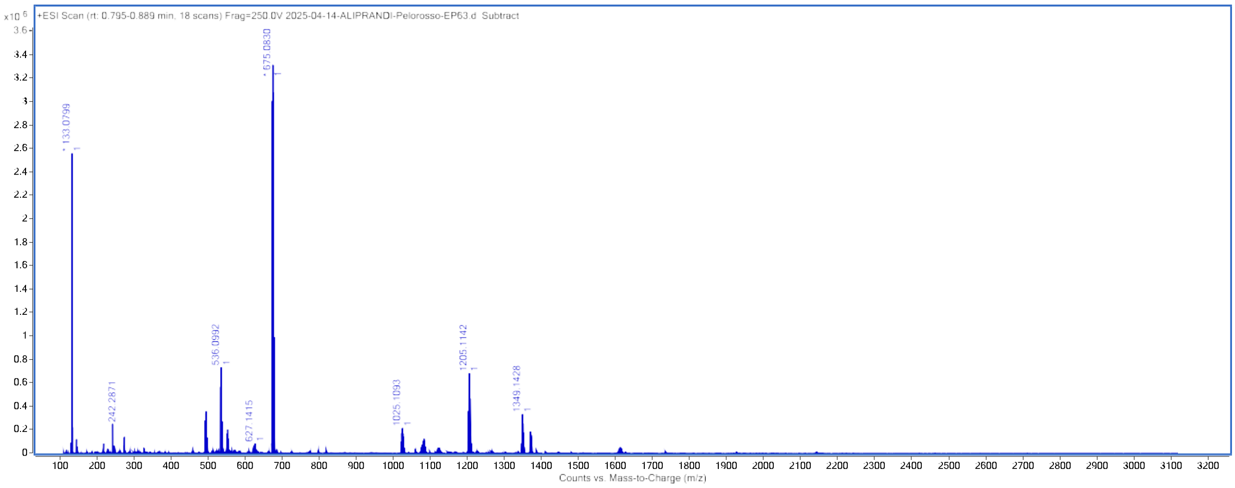

Figure S11. HR-MS ESI scan of complex **2** in positive mode.

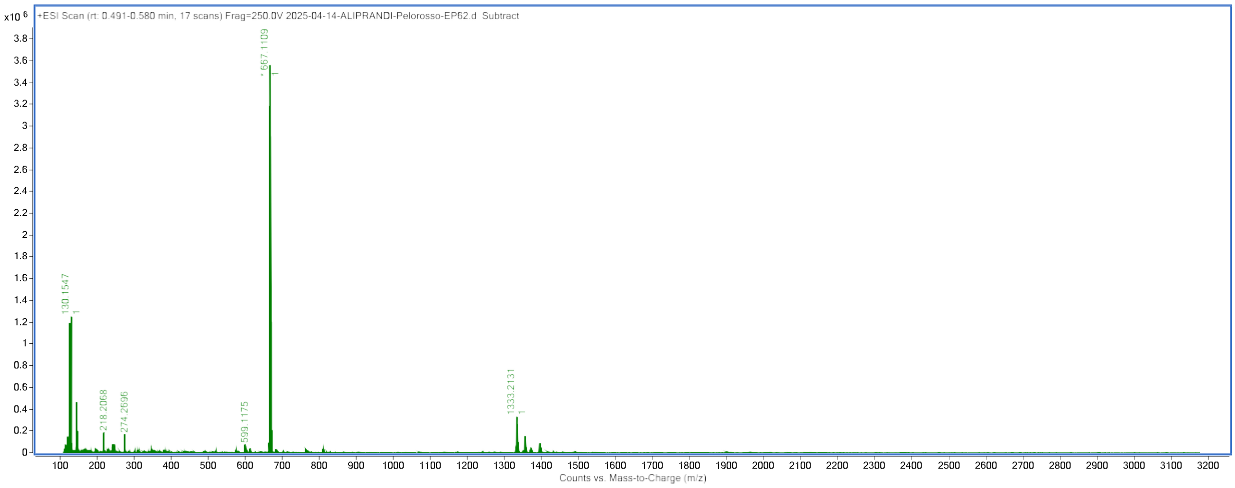

Figure S12. HR-MS ESI scan of complex **3** in positive mode.

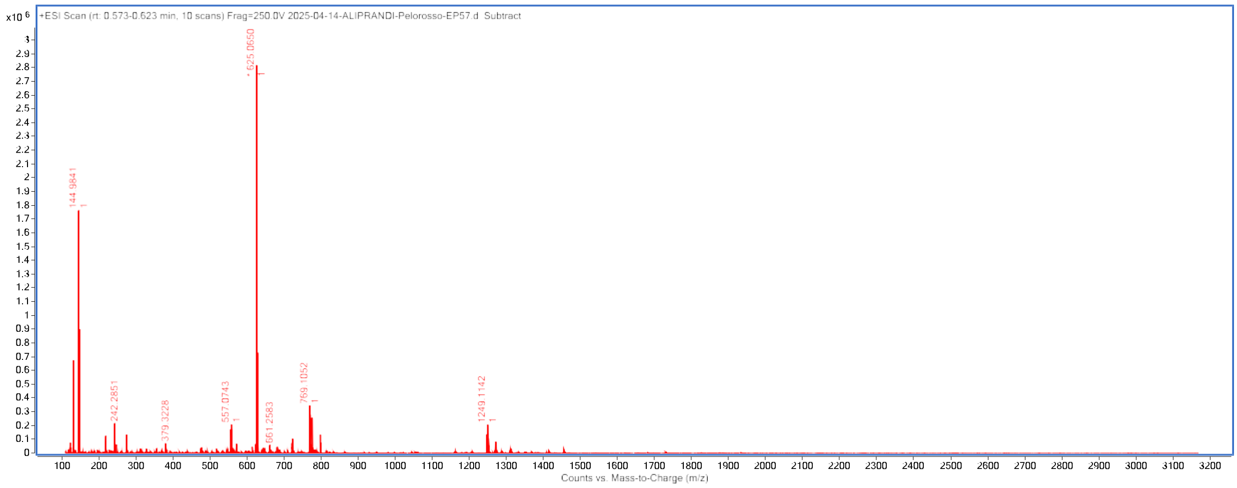

Figure S13. HR-MS ESI scan of complex **4** in positive mode.

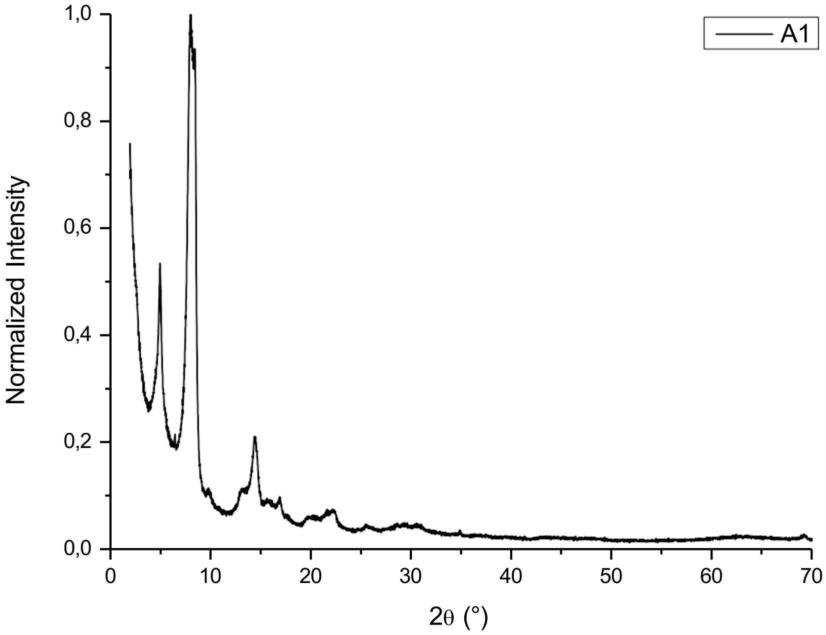

Figure S14. Powder XRD of a drop cast of a 5mg/mL solution of **1** (forming **A1**) in $CHCl_3$.

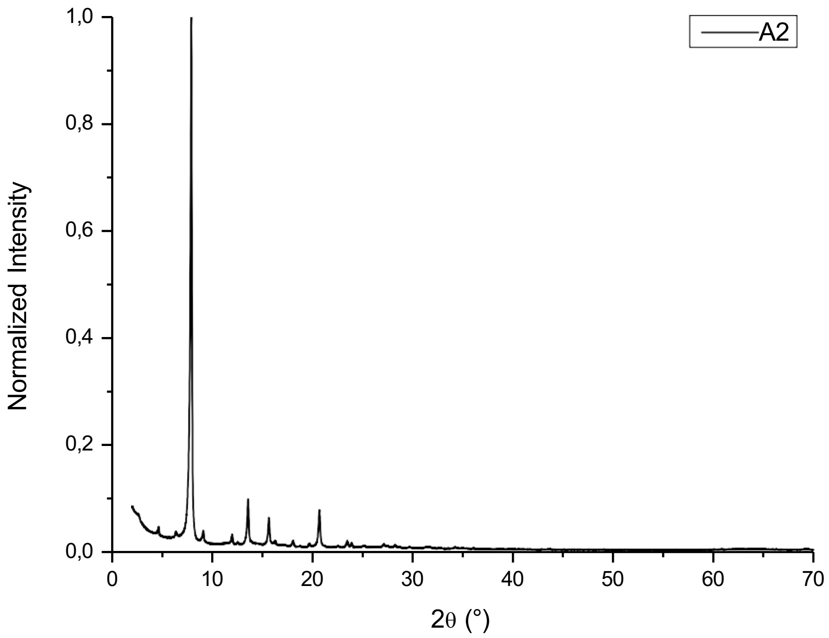

Figure S15. Powder XRD of a drop cast of a 5mg/mL solution of **2** (forming **A2**) in $CHCl_3$.

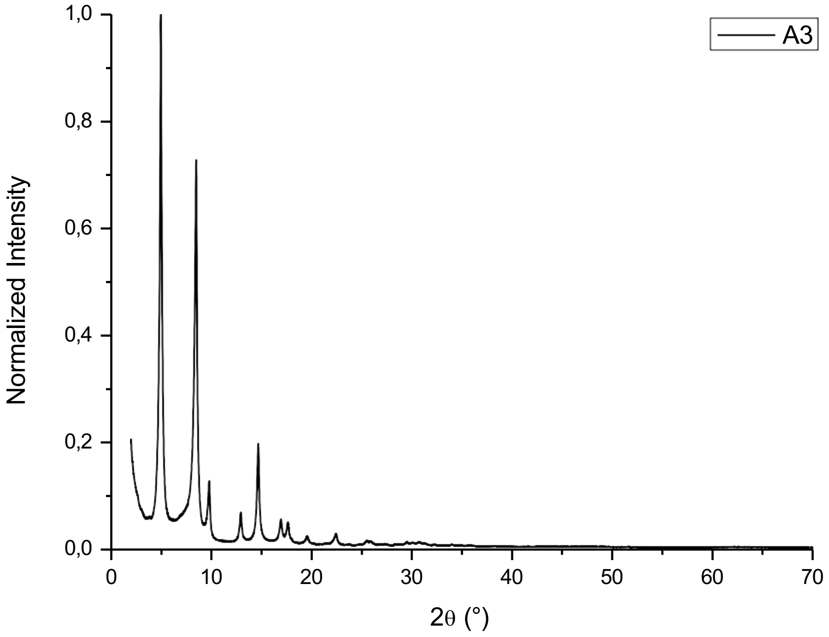

Figure S16. Powder XRD of a drop cast of a 5mg/mL solution of **3** (forming **A3**) in $CHCl_3$.

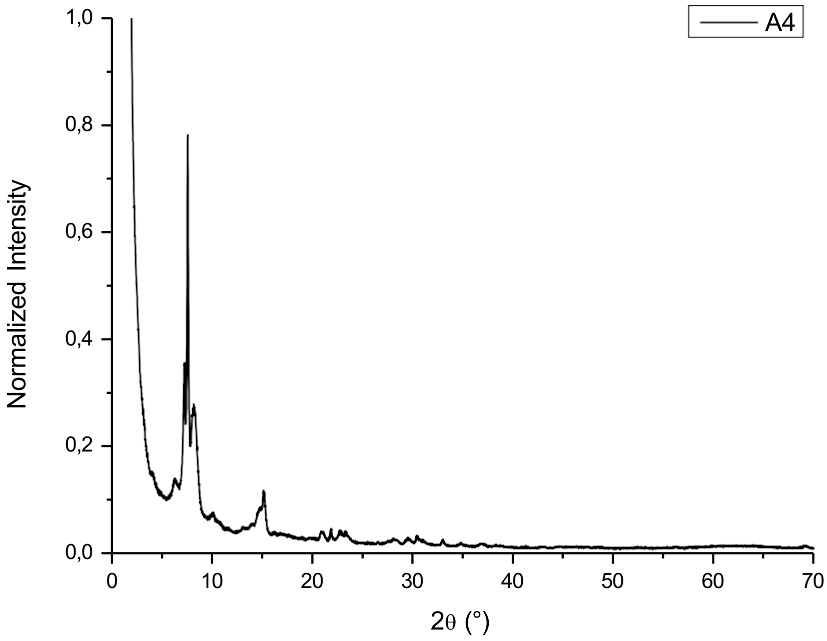

Figure S17. Powder XRD of a drop cast of a 5mg/mL solution of **4** (forming **A4**) in $CHCl_3$.

Table S1. Crystallographic data for 2.

Formula	C20.50 H12.50 Cl4.50 F6 N9 Pt
Molecular weight	853.51
Crystal system	Tetragonal
Space group	P 43 21 2
a/Å	20.334(4)
b/Å	20.334(4)
c/Å	13.825(3)
α/°	90
β/°	90
γ/°	90
Volume, Å^3	5716(3)
T (K)	295(2)
Z	8
D_{calc}/g cm^{-3}	1.984
F(000)	3256.0
μ(Mo–Kα)/mm^{-1}	5.399
Reflections collected	395921
Unique reflections	5857
Observed reflections [I>2σ(I)]	5195 [R_{int} = 0.0228]
R [I > 2σ(I)]	R_1 = 0.0238
	wR_2 = 0.0629
R [all data]	R_1 = 0.0305
	wR_2 = 0.0662

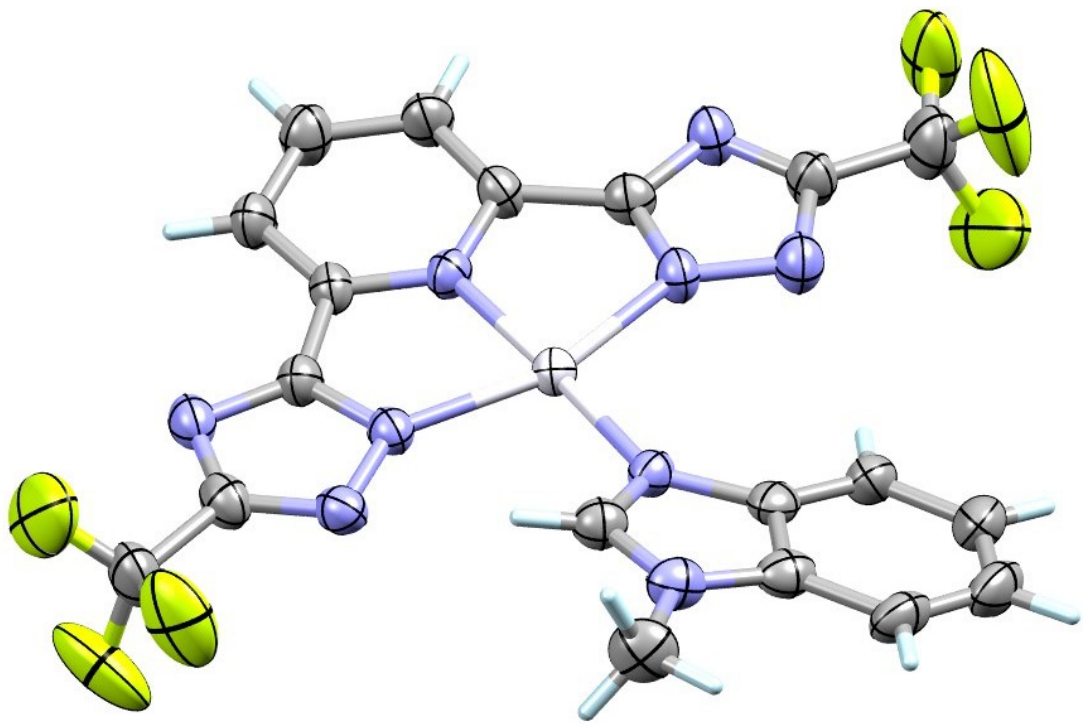

Figure S18. ORTEP view of compound **2.** Thermal ellipsoids are displayed at 30% probability level. Solvent molecules are omitted for clarity. Color code: light gray (Pt), light blue (N), yellowish (F), gray (C), cyan (H).

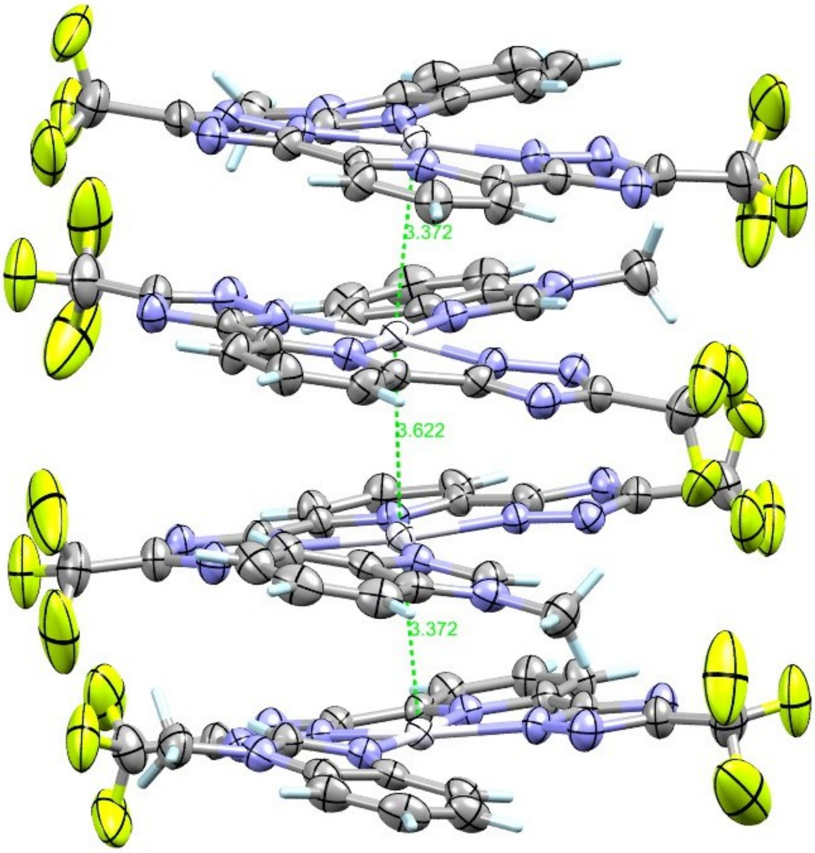

Figure S19. Stacking of molecules of compound **2** evidencing interactions between Pt atoms. Color code: light gray (Pt), light blue (N), yellowish (F), gray (C), cyan (H).

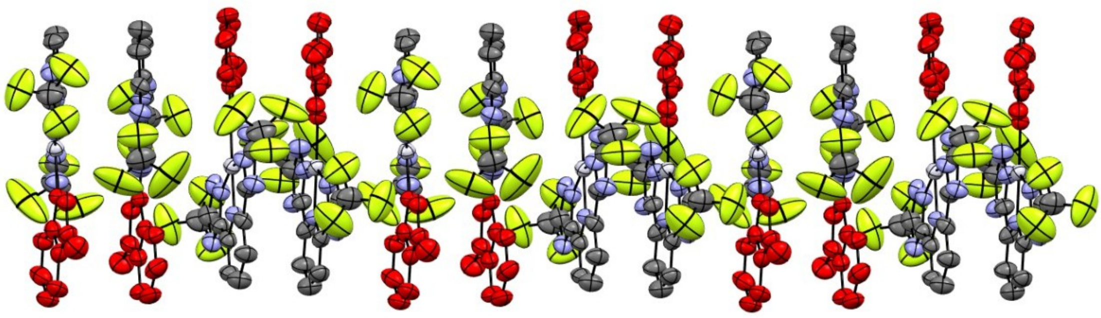

Figure S20. Crystal packing of complex **2**, highlighting the 1-methylbenzimidazole moieties (in red), which alternate within the structure every two units.

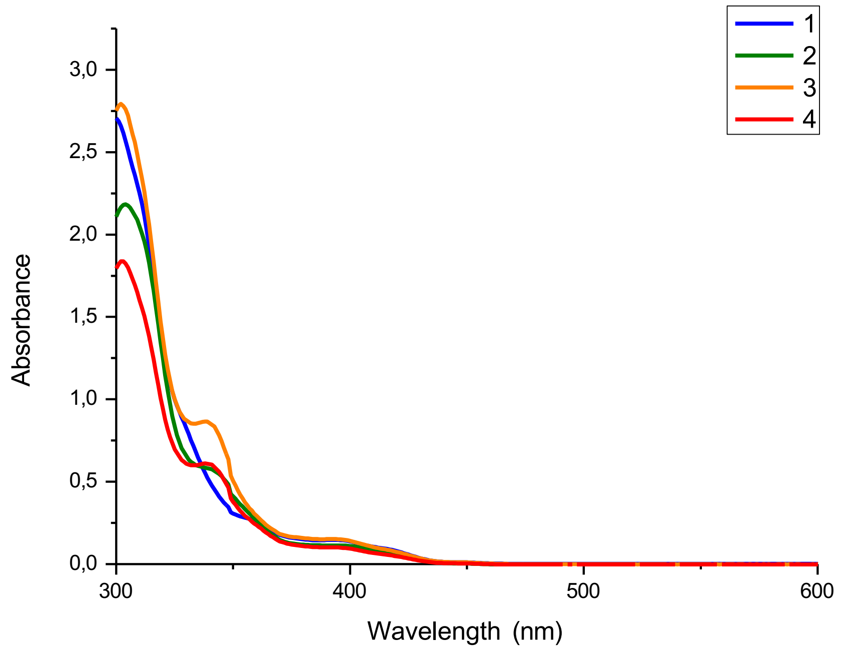

Figure S21. Absorption spectra of [complex] = 100µM in DMF. Complex **1** (blue), **2** (green), **3** (yellow) and **4** (red).

Properties of aggregates

Table S2. Photophysical properties of complexes **A1-A4** in $CHCl_3$.

**Complexes**	**λ_{em} (nm)**	**ϕ_{PL}(%)**
A1	462, 490, 523	11.6 ± 0.9
A2	520	3.87 ± 0.08
A3<br>A3a	580<br>534	7 ± 2<br>34 ± 2
A4	598	12 ± 1

[Complex]= 5mg/mL in $CHCl_3$. λ_{exc}= 350-500 nm. Absolute φ_{PL} (%).

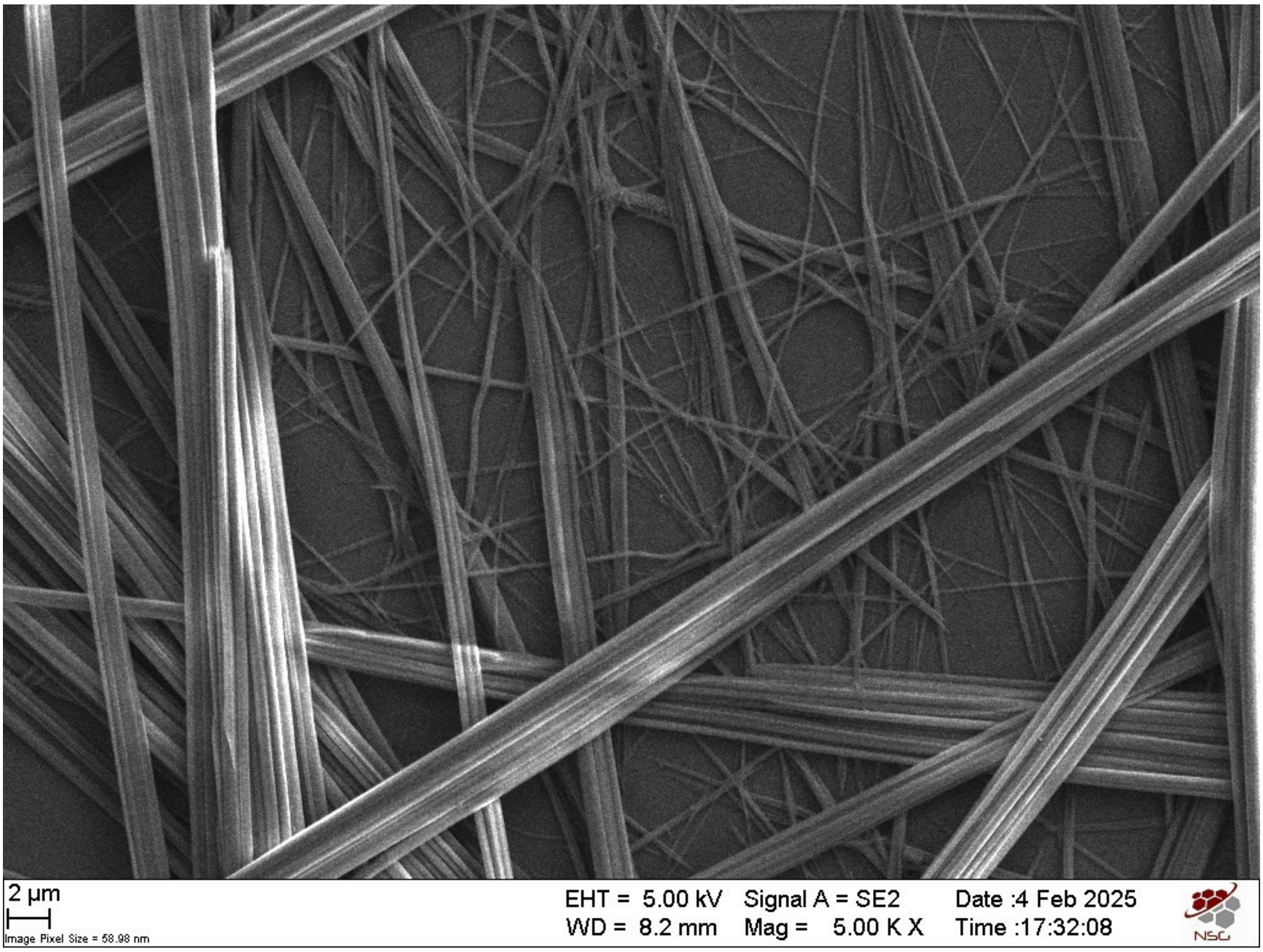

Figure S22. SEM analysis of a drop cast of a 5mg/mL solution of **1** in $CHCl_3$.

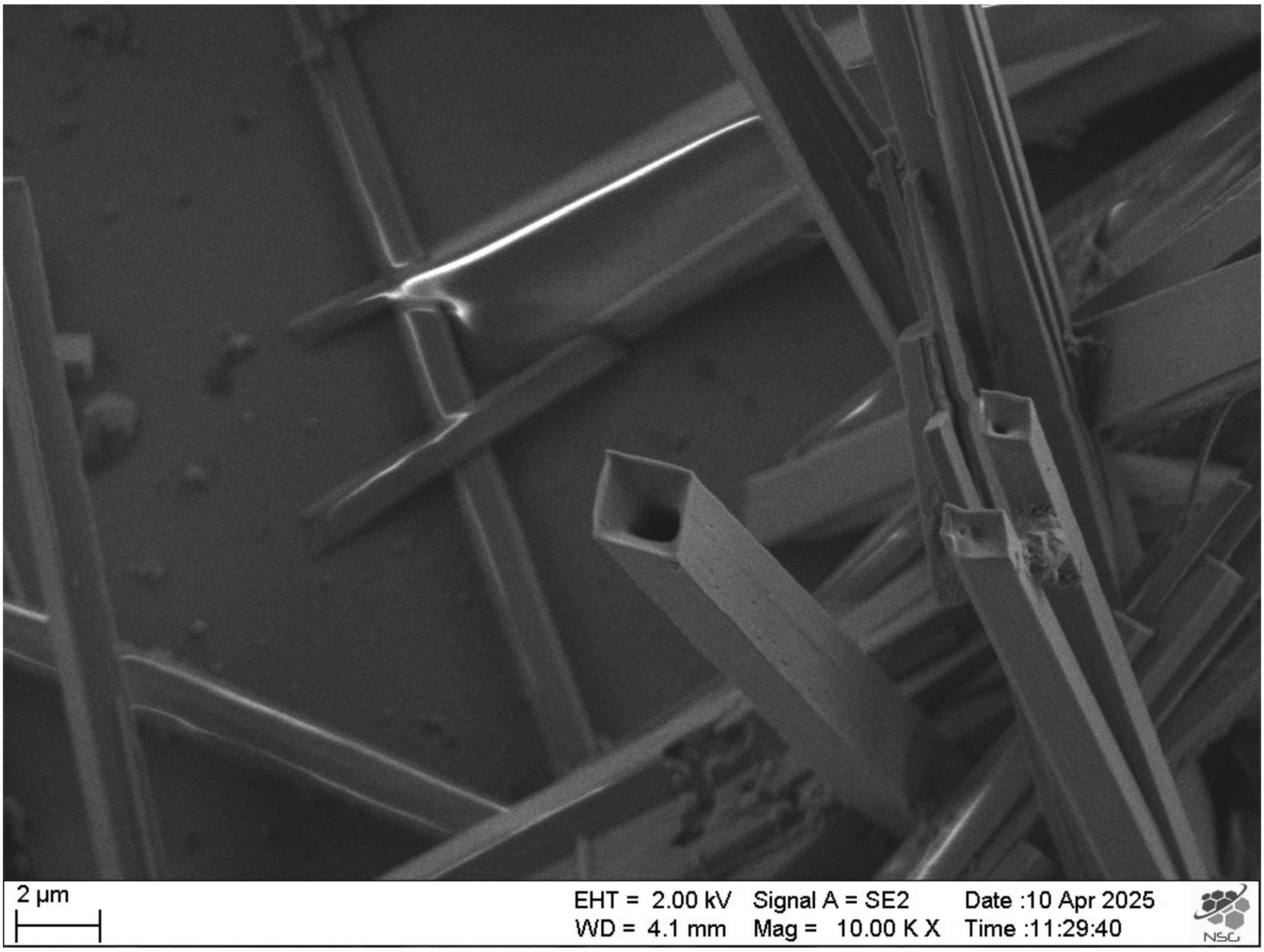

Figure S23. SEM analysis of a drop cast of a 5mg/mL solution of **2** in $CHCl_3$.

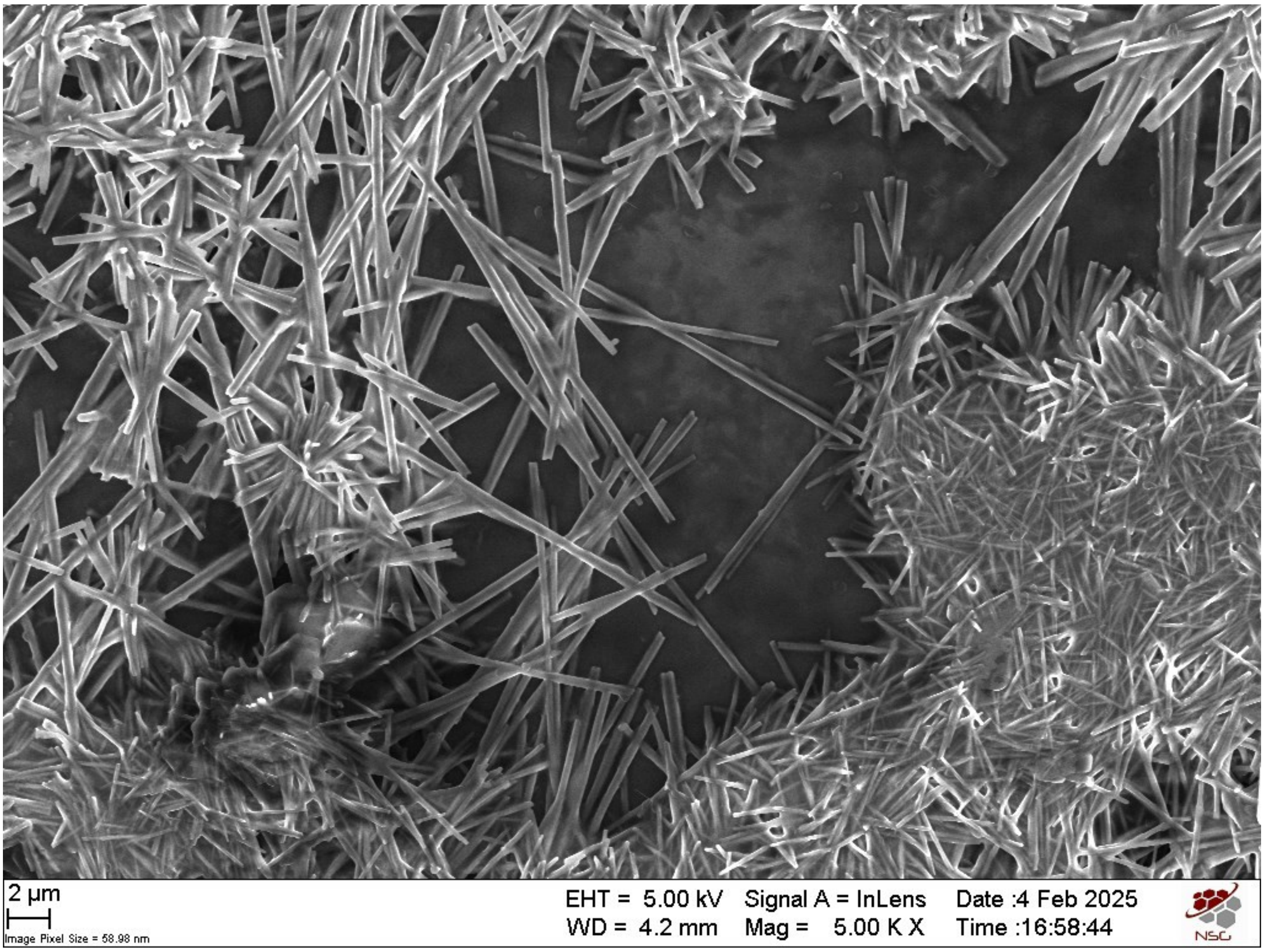

Figure S24. SEM analysis of a drop cast of a 5mg/mL solution of **3** in $CHCl_3$.

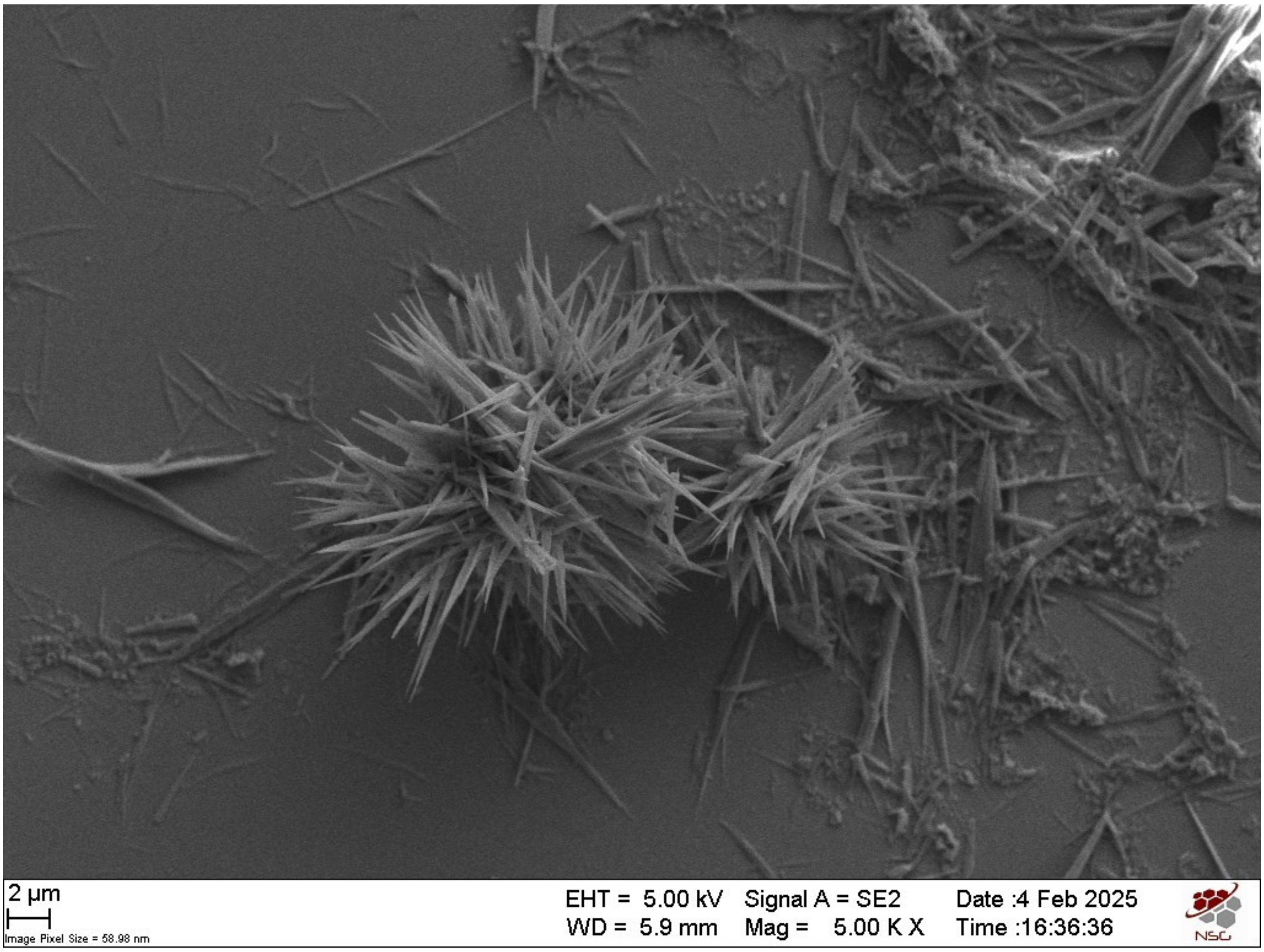

Figure S25. SEM analysis of a drop cast of a 5mg/mL solution of **4** in $CHCl_3$.

Ligand exchange processes

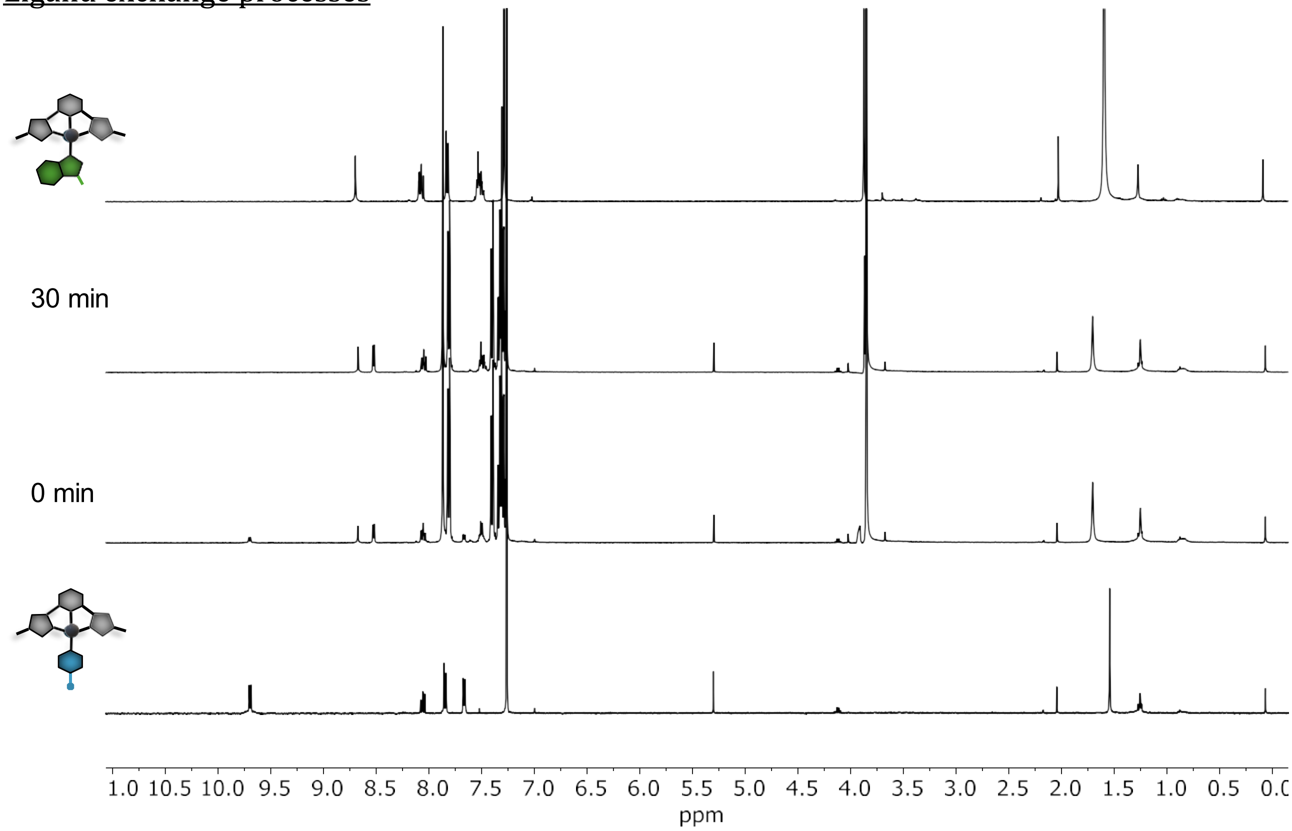

Figure S26. Time evolution ^{1}H NMR full spectra of ligand exchange reaction of complex **1** with **L2** in $CDCl_3$.

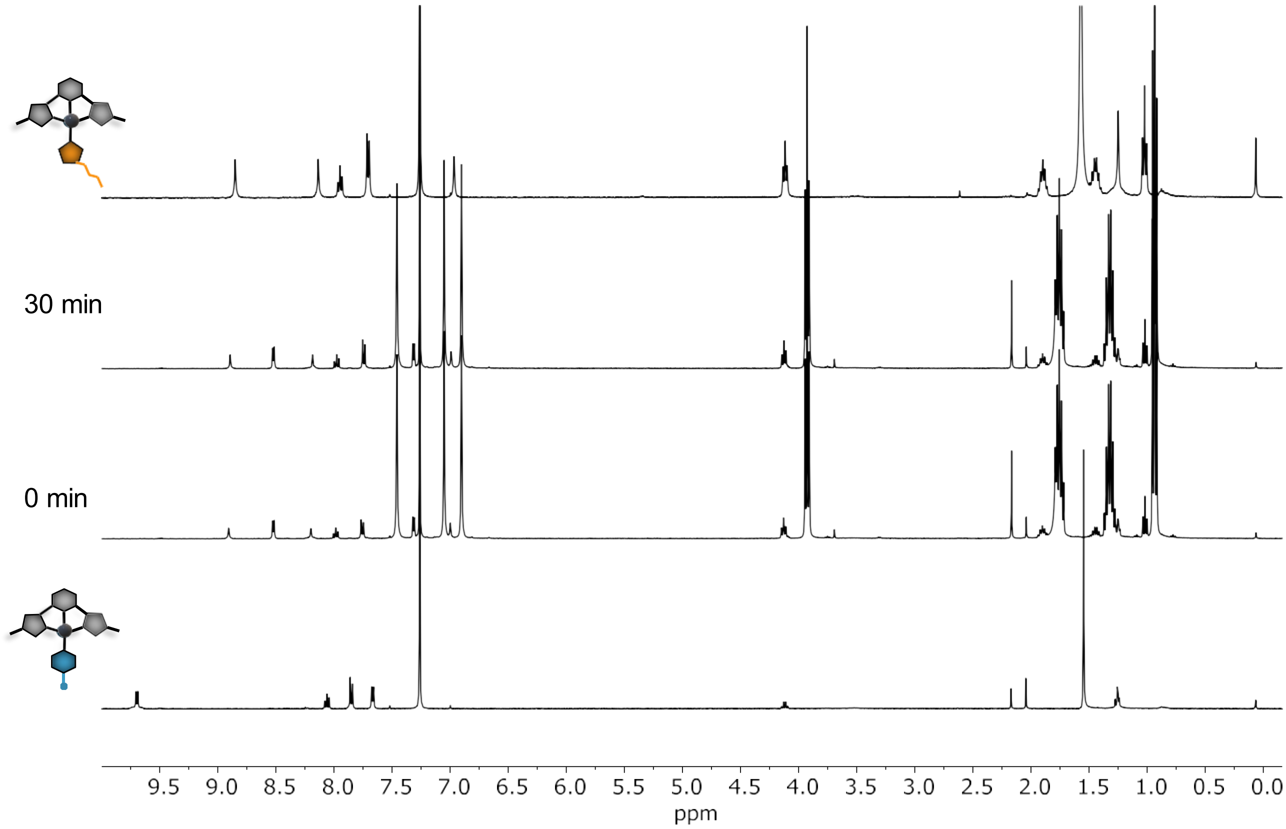

Figure S27. Time evolution ^{1}H NMR full spectra of ligand exchange reaction of complex **1** with **L3** in $CDCl_3$.

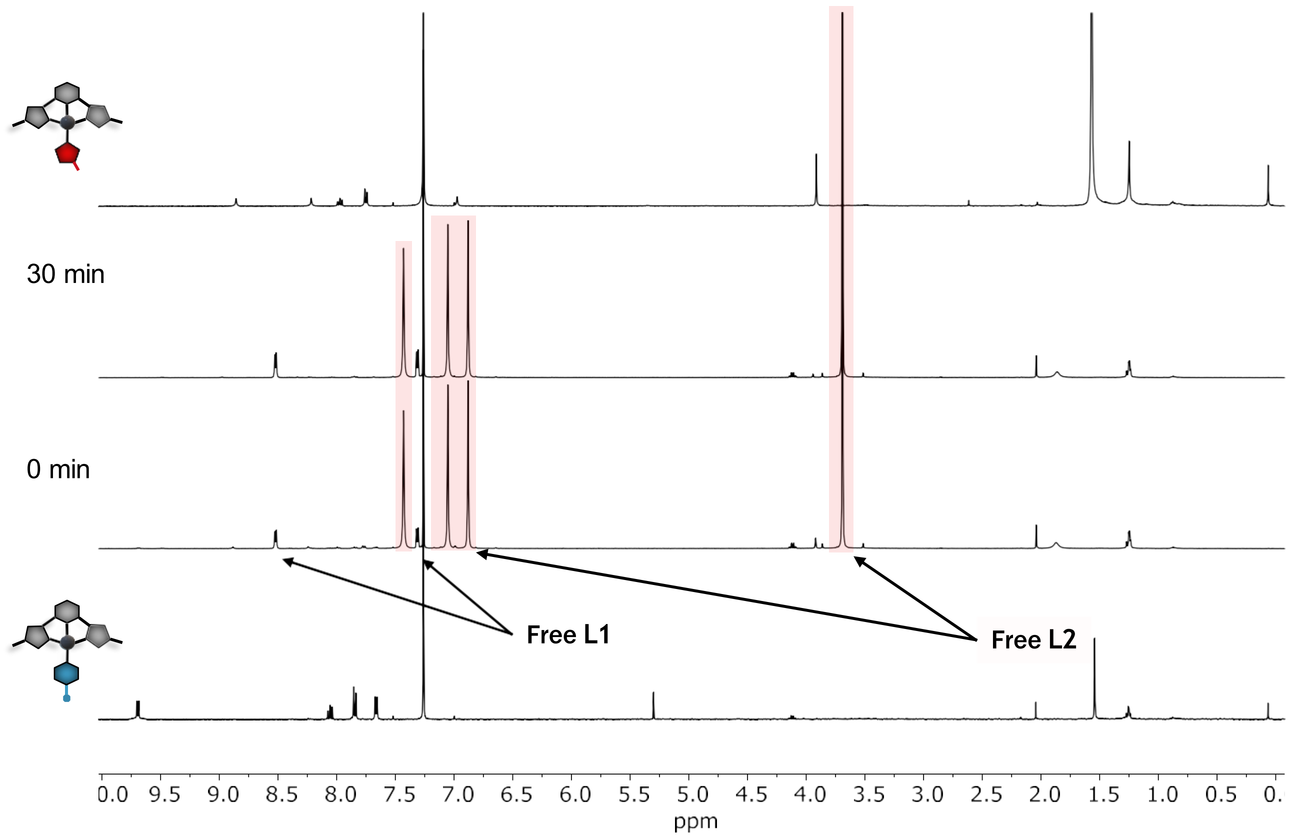

Figure S28. Time evolution ^1H NMR full spectra of ligand exchange reaction of complex **1** with **L4** in $CDCl_3$.

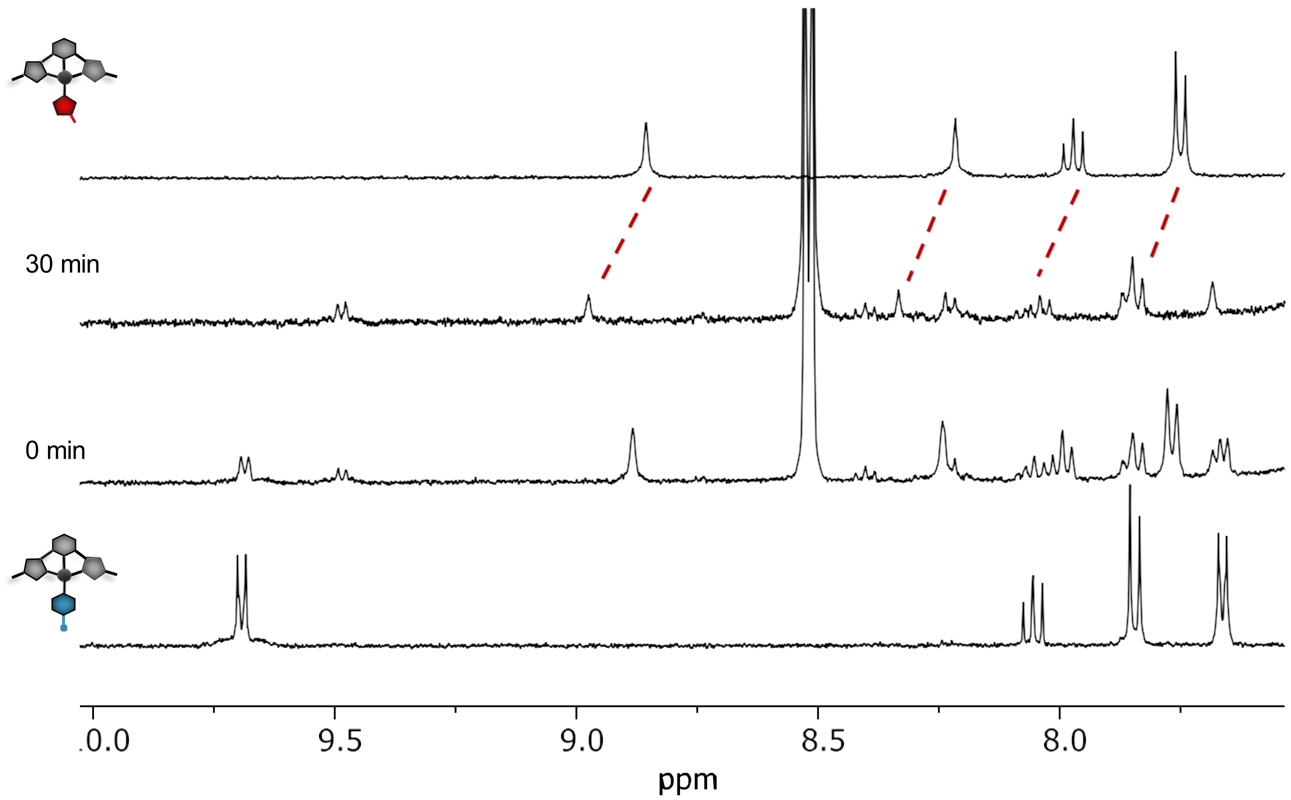

Figure S29. Zoom of time evolution ^1H NMR spectra of ligand exchange reaction of complex **1** with **L2** in $CDCl_3$.

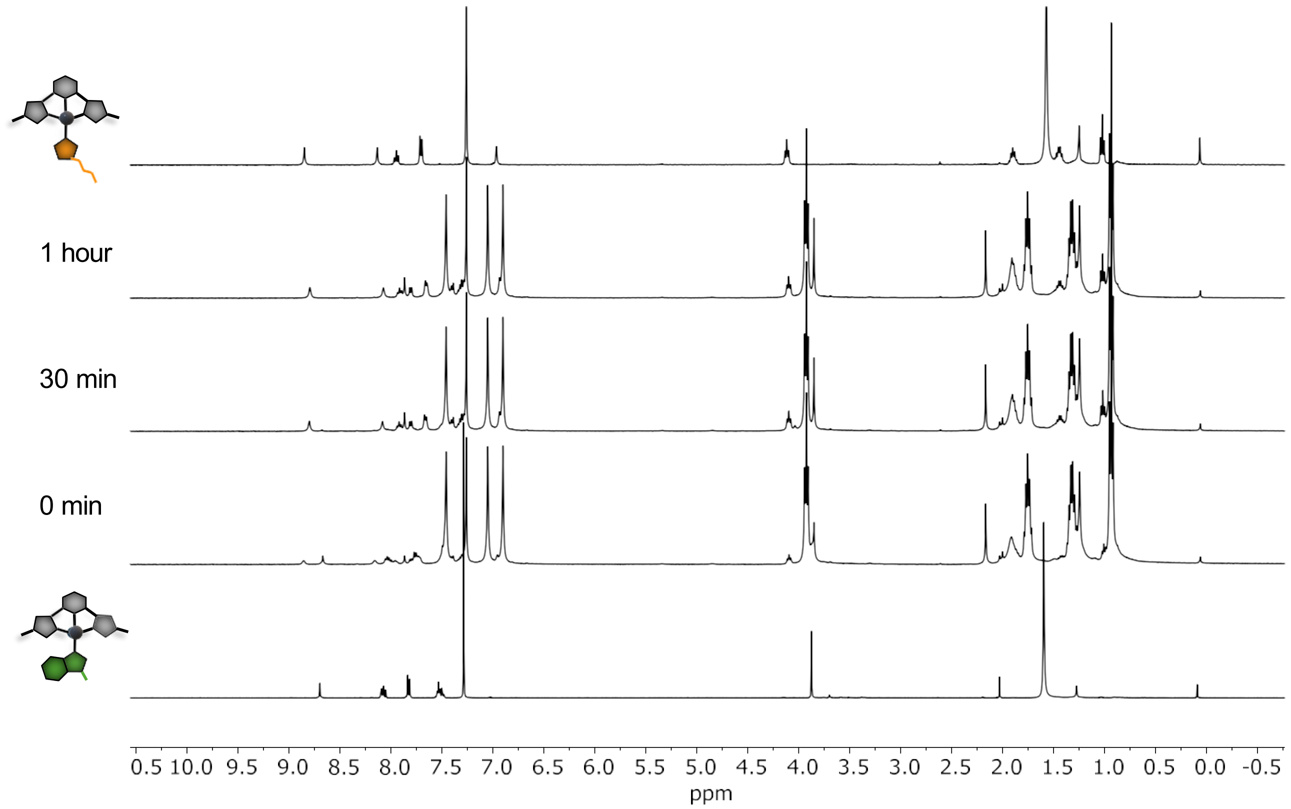

Figure S30. Time evolution ^{1}H NMR spectra of ligand exchange reaction of complex **2** with **L3** in $CDCl_3$.

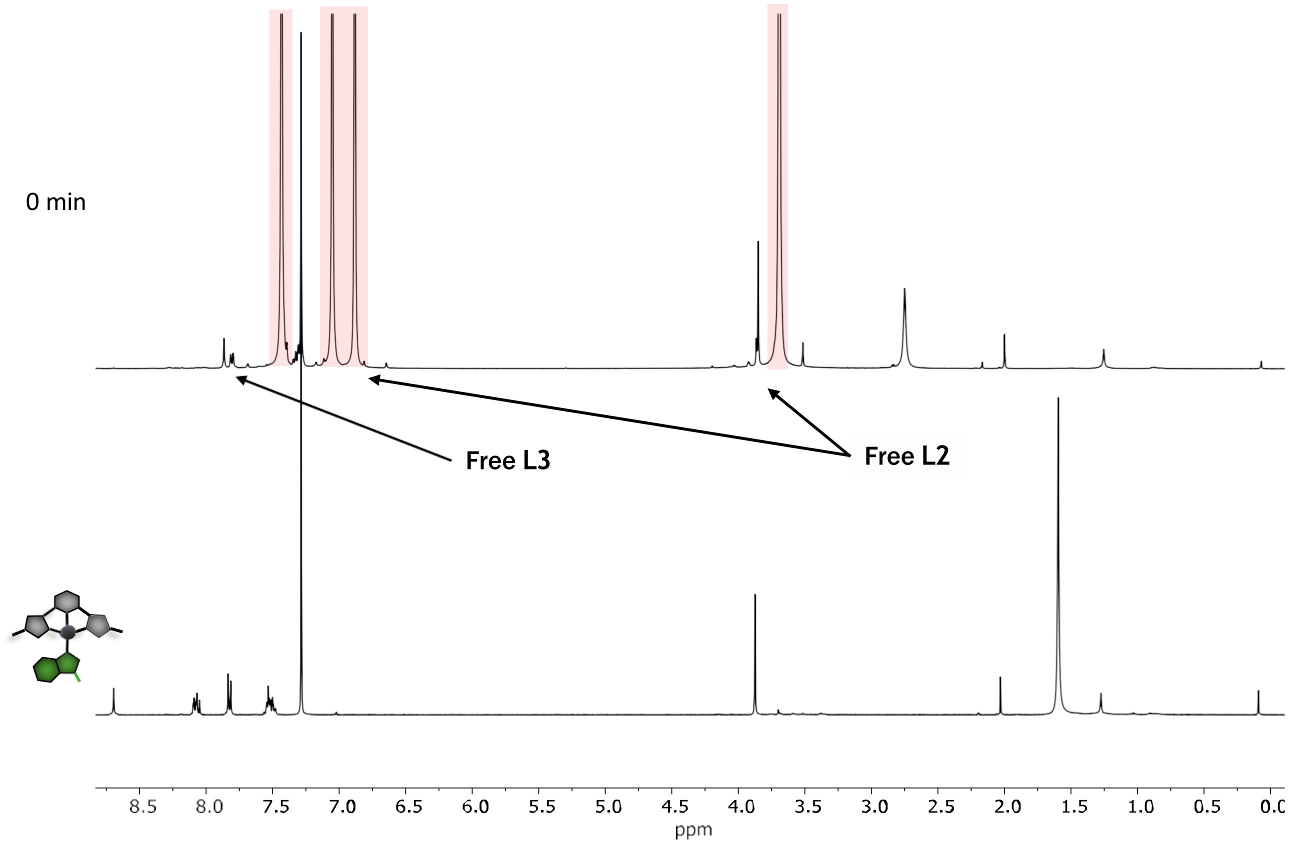

Figure S31. Time evolution ^{1}H NMR spectra of ligand exchange reaction of complex **2** with **L4** in $CDCl_3$.

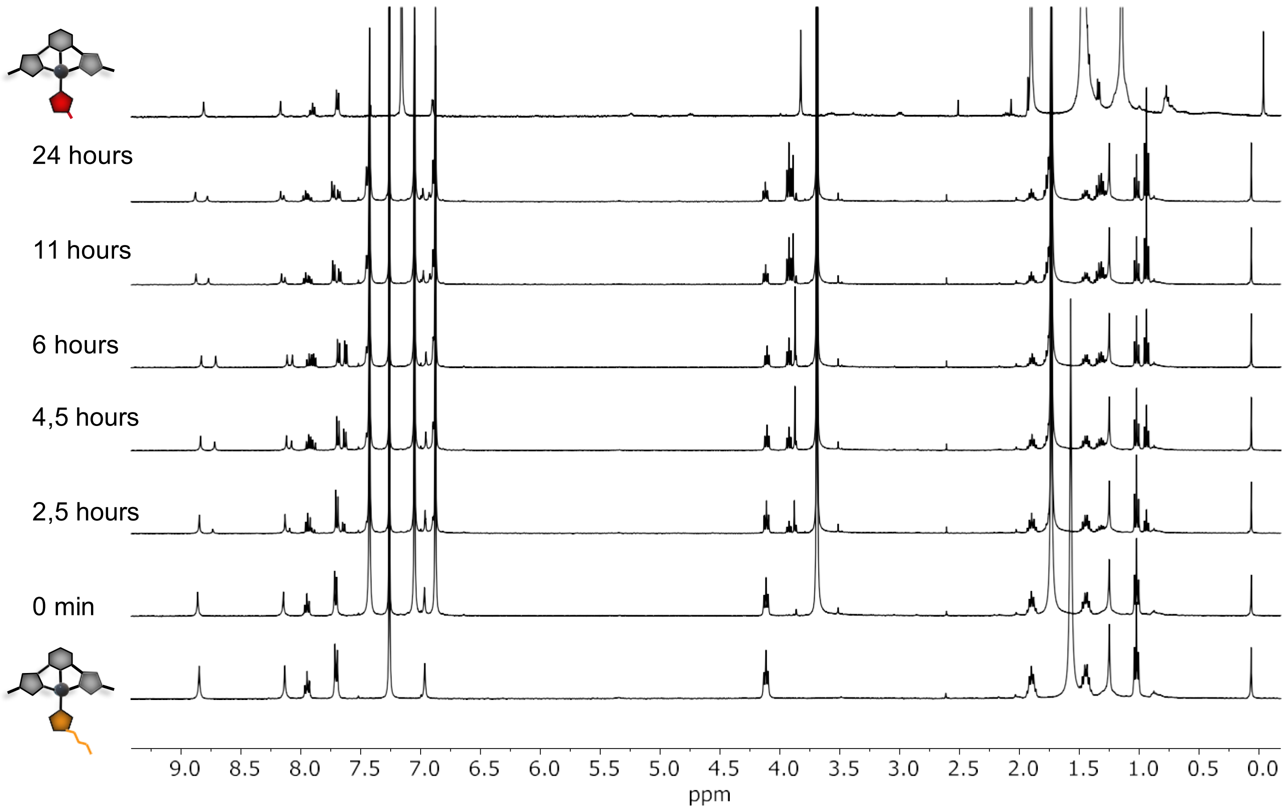

Figure S32. Time evolution ^{1}H NMR spectra of ligand exchange reaction of complex **3** with **L4** in $CDCl_3$.

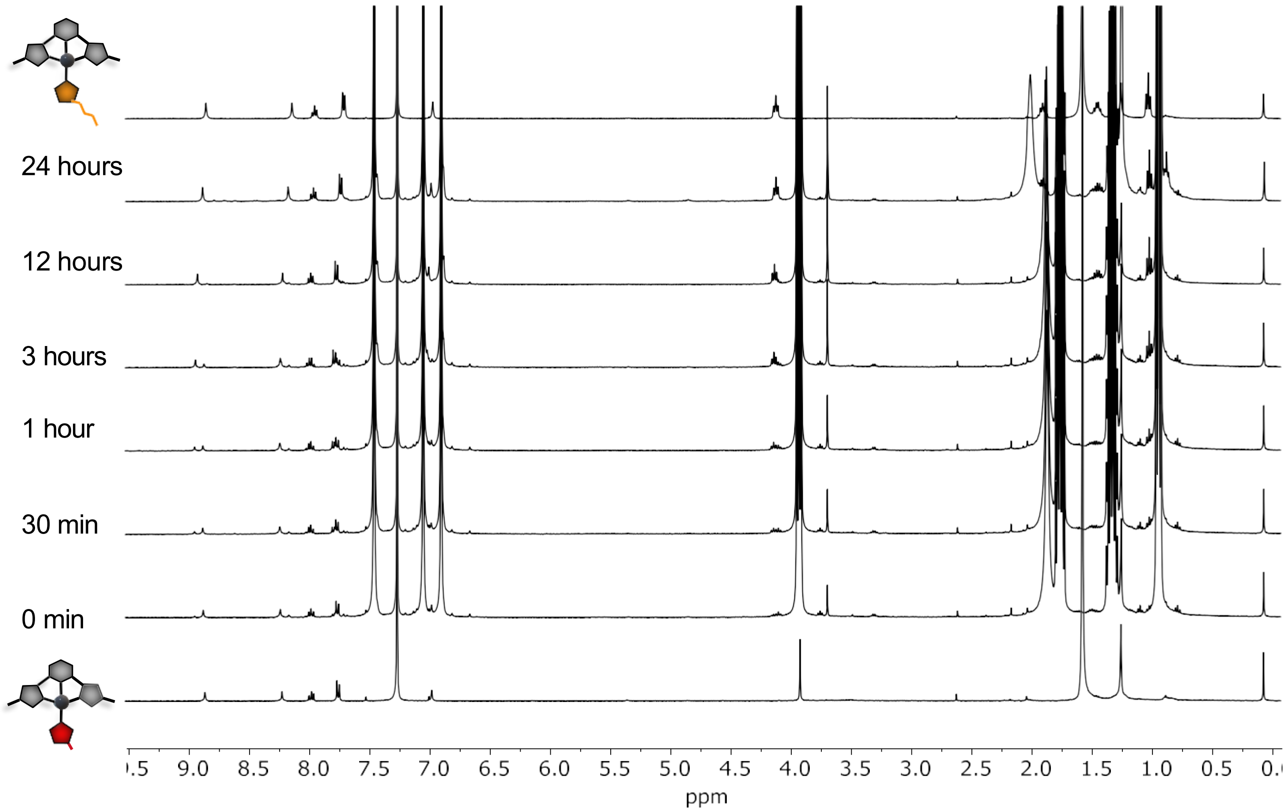

Figure S33. Time evolution ^{1}H NMR spectra of ligand exchange reaction of complex **4** with **L3** in $CDCl_3$.

Table S3. Binding constant for **L2-L4**

**Compounds**	K (M^{-1})
A2	4 ± 2
A3	141 ± 9
A4	536 ± 14

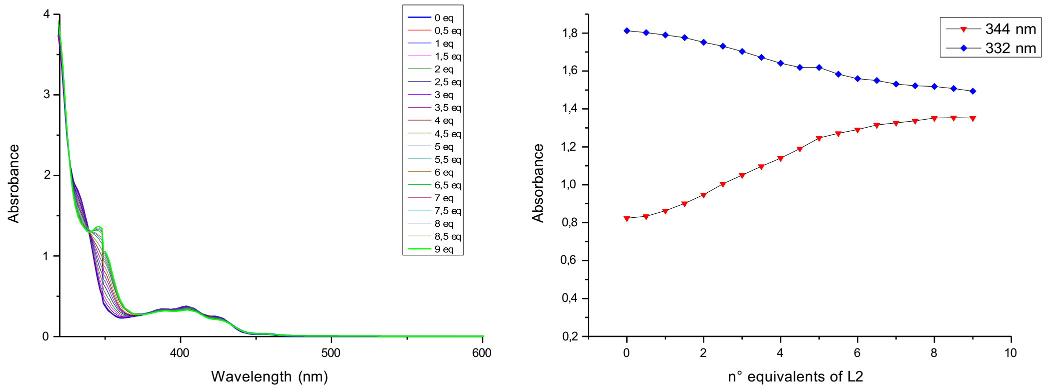

<u>Figure S34.</u> UV-vis spectra recorded during **A1** titration with **L2** (left) and corresponding binding isotherm at 344 and 332 nm (right).

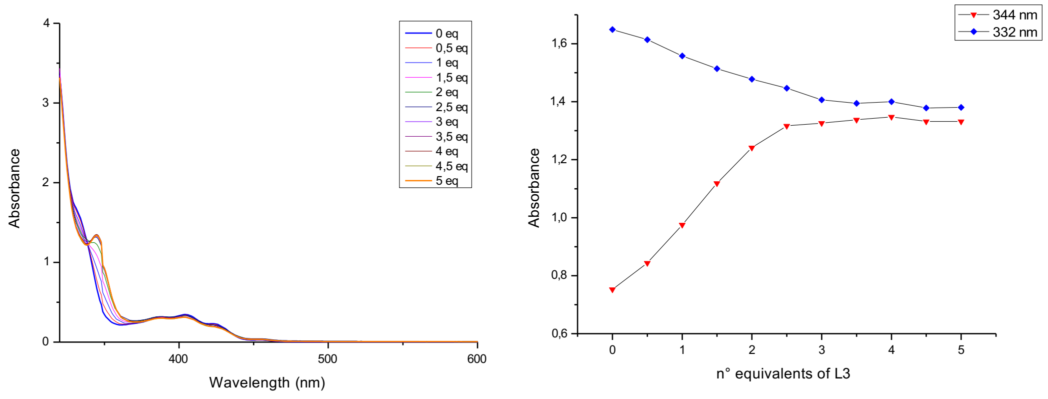

<u>Figure S35.</u> UV-vis spectra recorded during **A1** titration with **L3** (left) and corresponding binding isotherm at 344 and 332 nm (right).

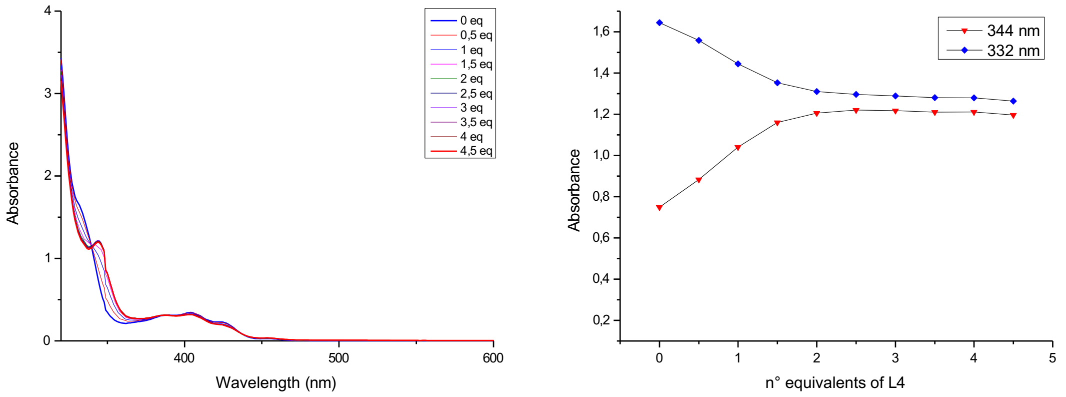

<u>Figure S36.</u> UV-vis spectra recorded during **A1** titration with **L4** (left) and corresponding binding isotherm at 344 and 332 nm (right).

<u>Exchange Product analysis</u>

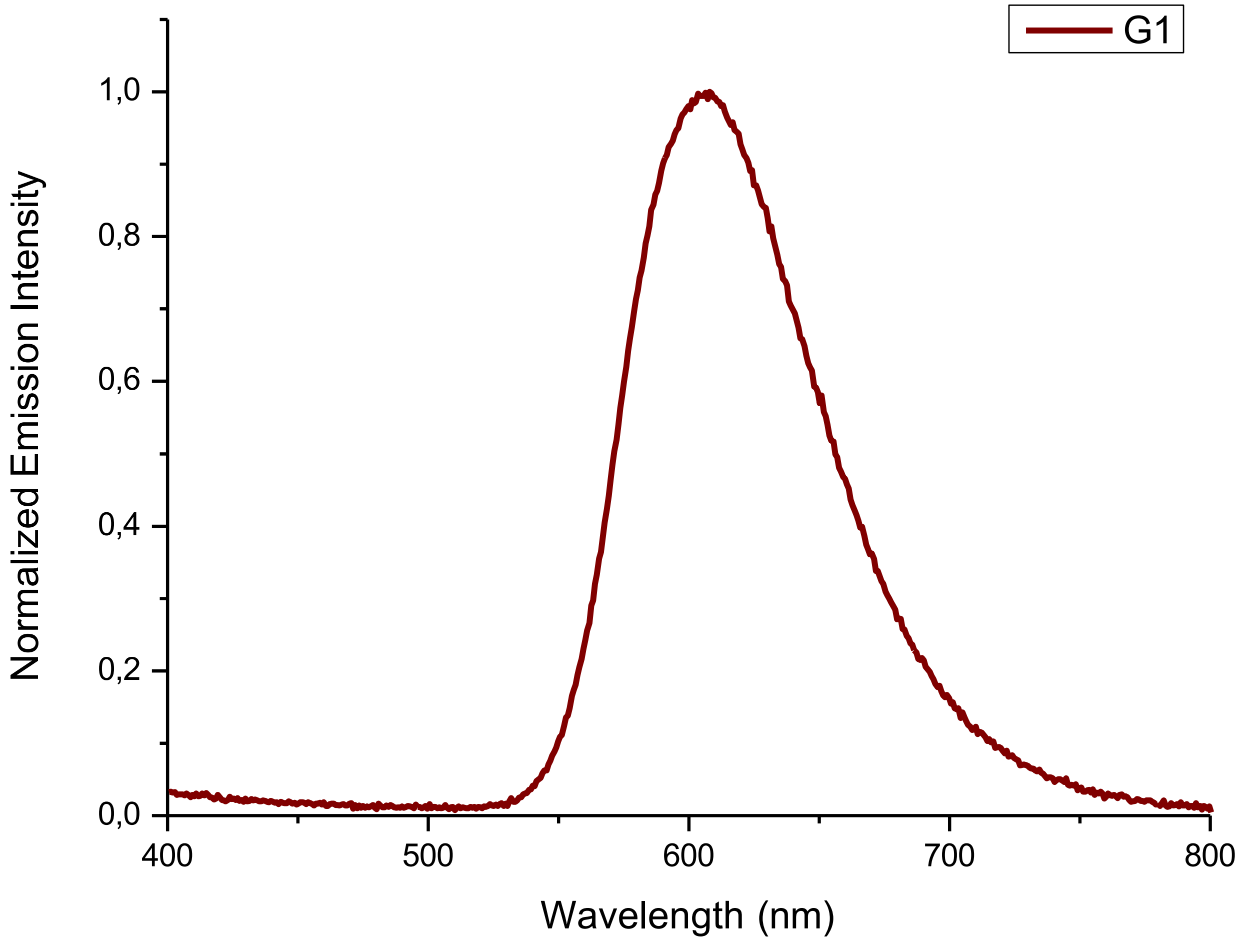

Figure S37. Normalized emission spectrum of **G1**, λ_{exc}= 360 nm.

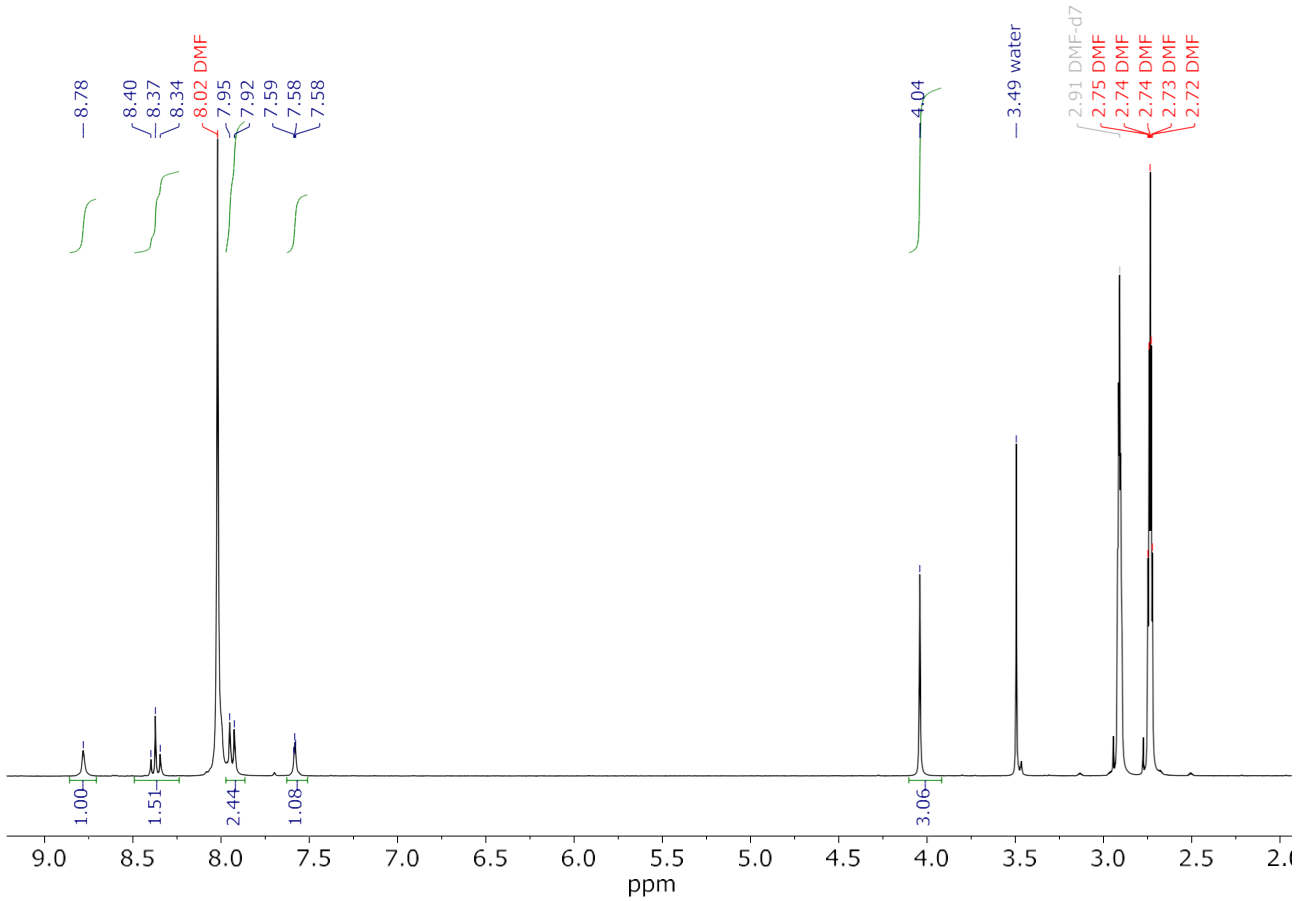

Figure S38. ^{1}H NMR spectrum (400MHz) of dried and washed **G1** in DMF-d_7 at 25°C.

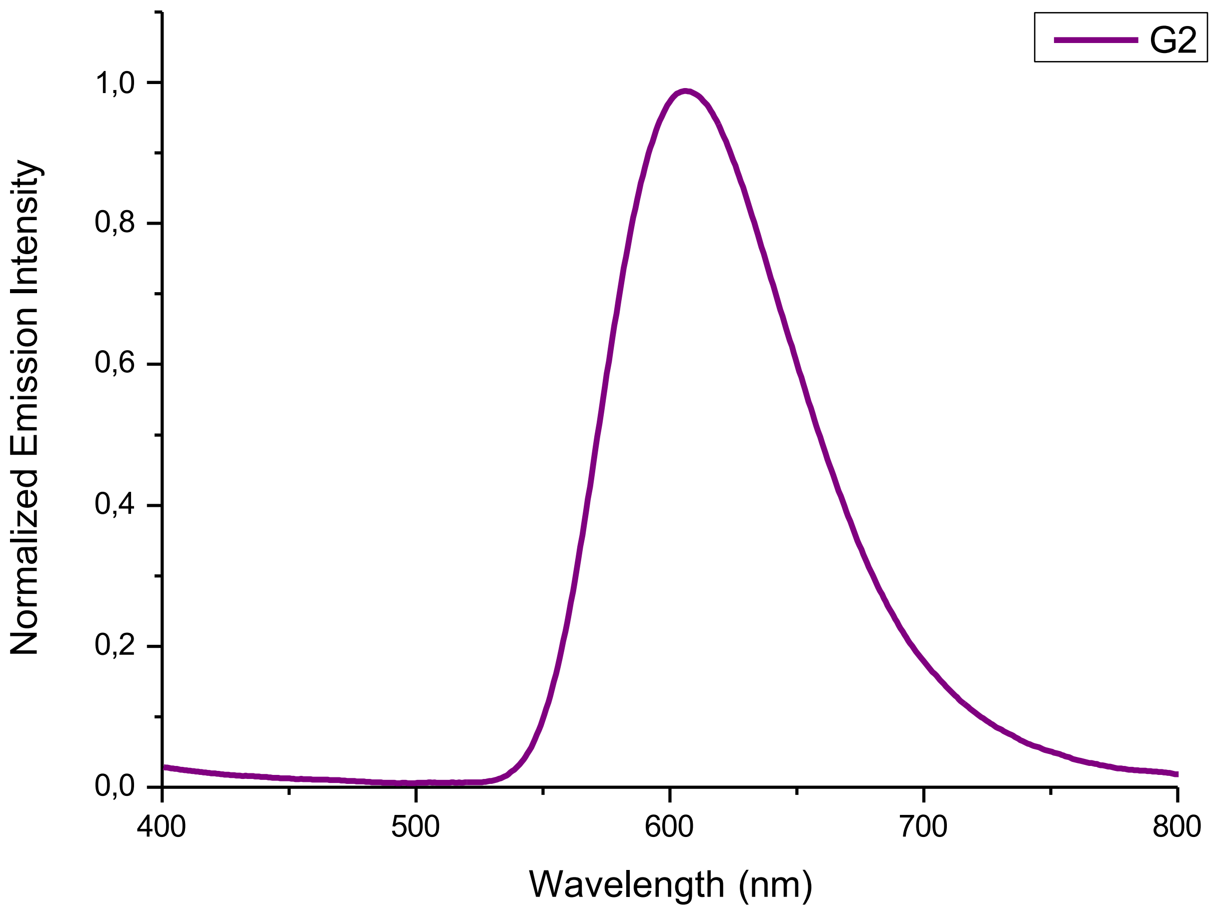

Figure S39. Normalized emission spectrum of **G2**, λ_{exc}= 360 nm.

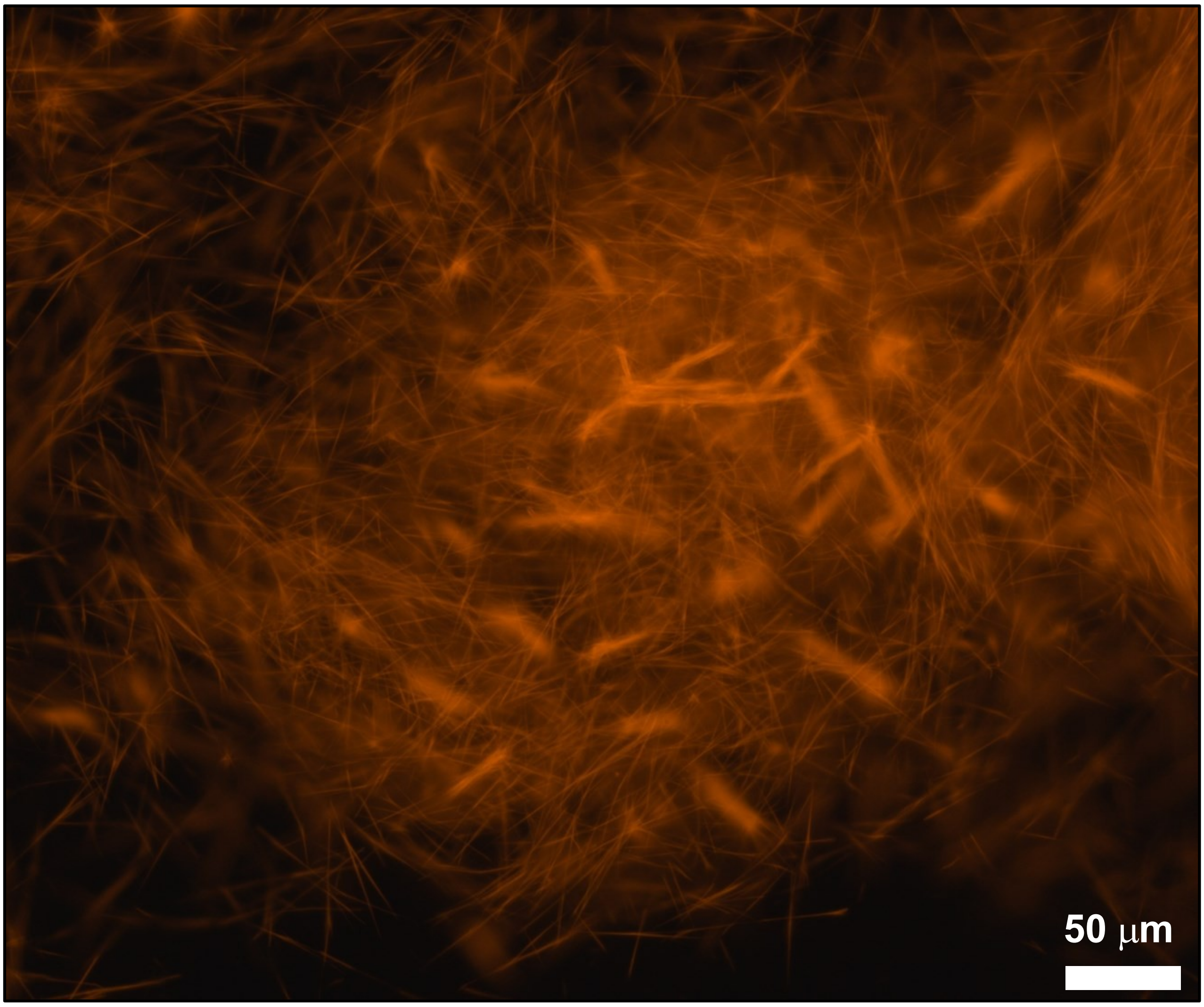

Figure S40. Fluorescence microscopy image of **G2**.

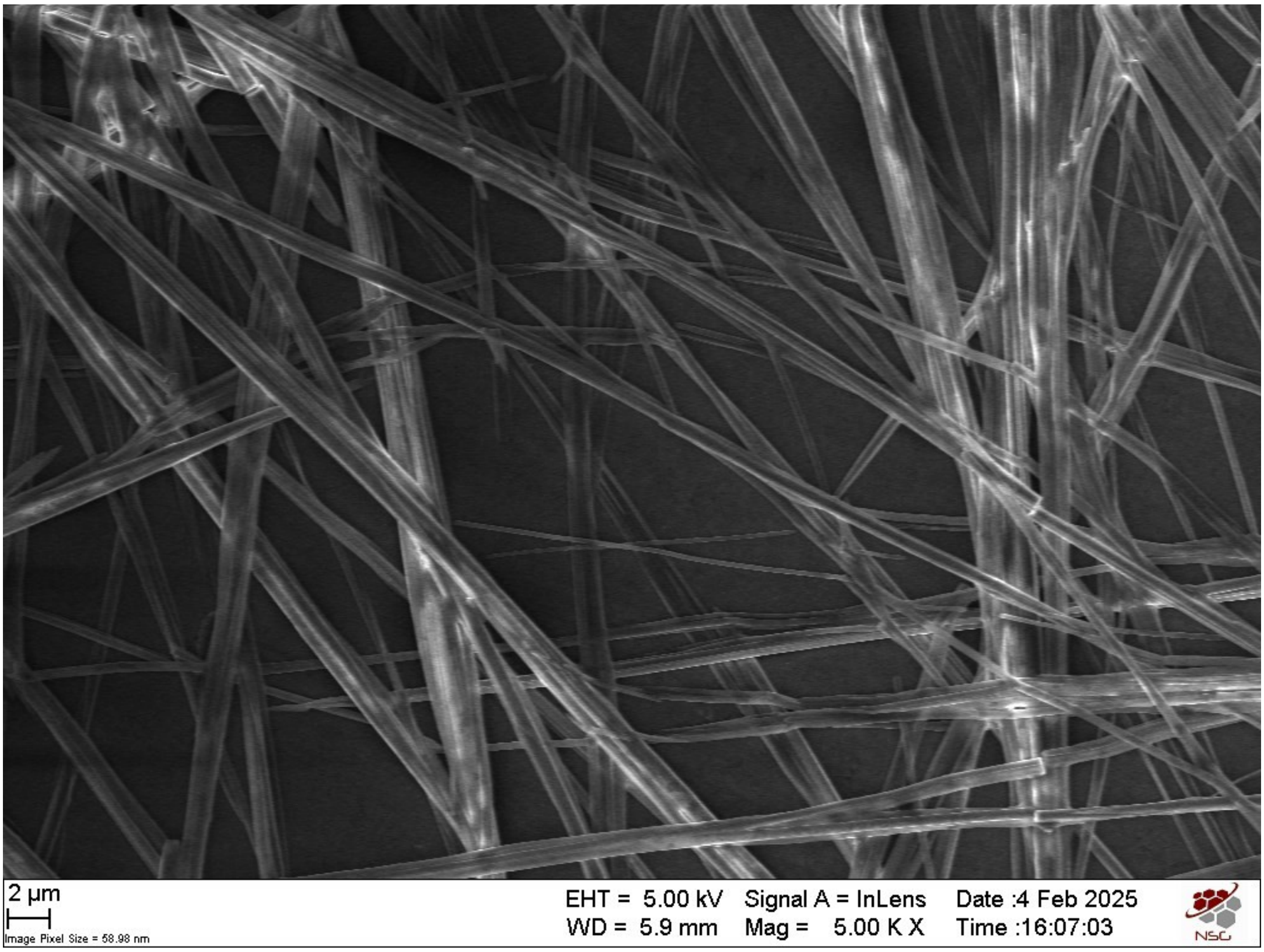

Figure S41. SEM analysis of **G1**.

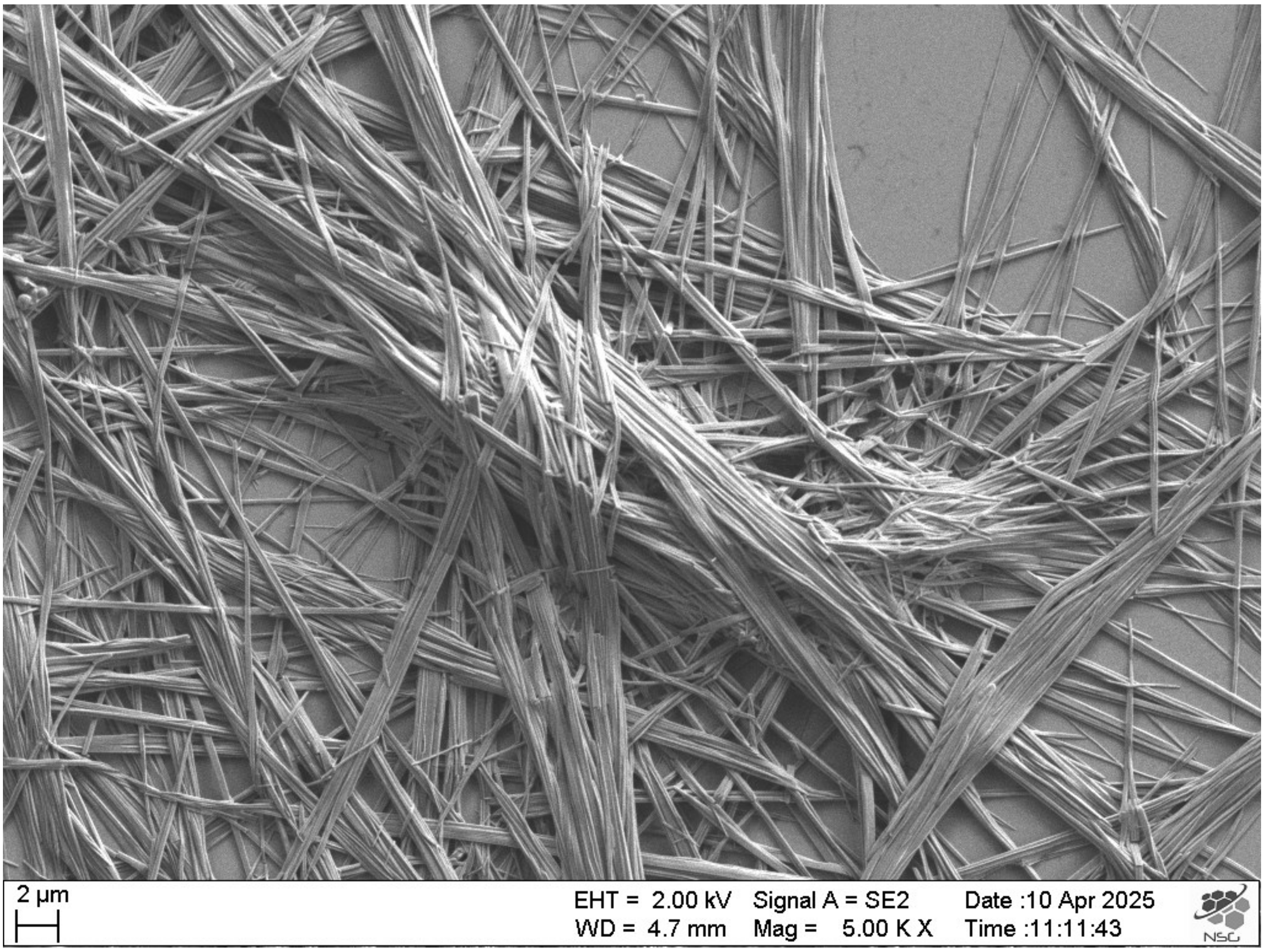

Figure S42. SEM analysis of **G2**.

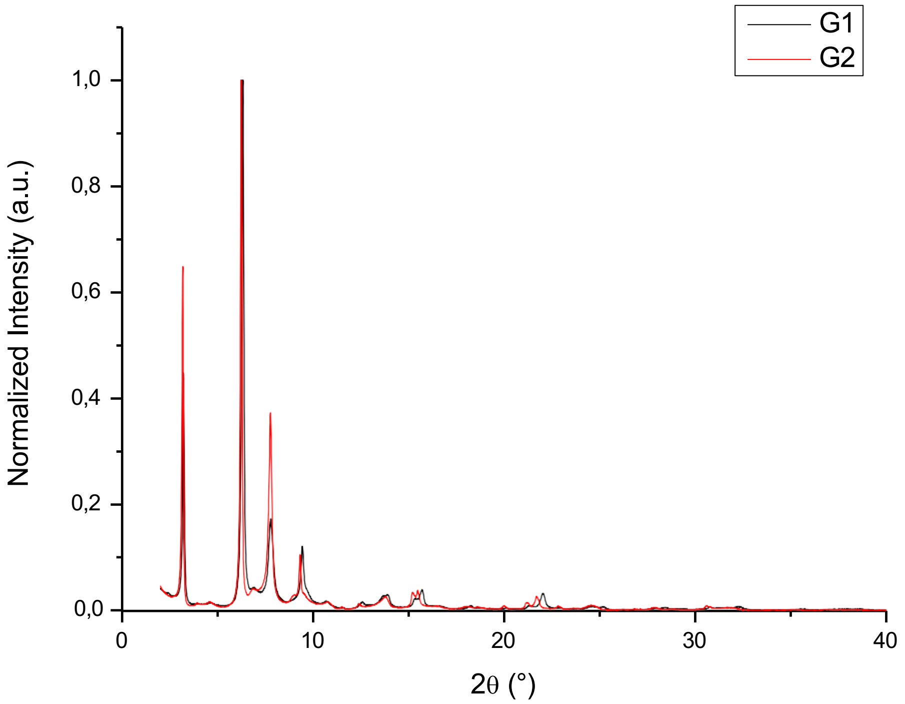

Figure S43. Powder XRD of a drop cast **G1** (black) and **G2** (red).

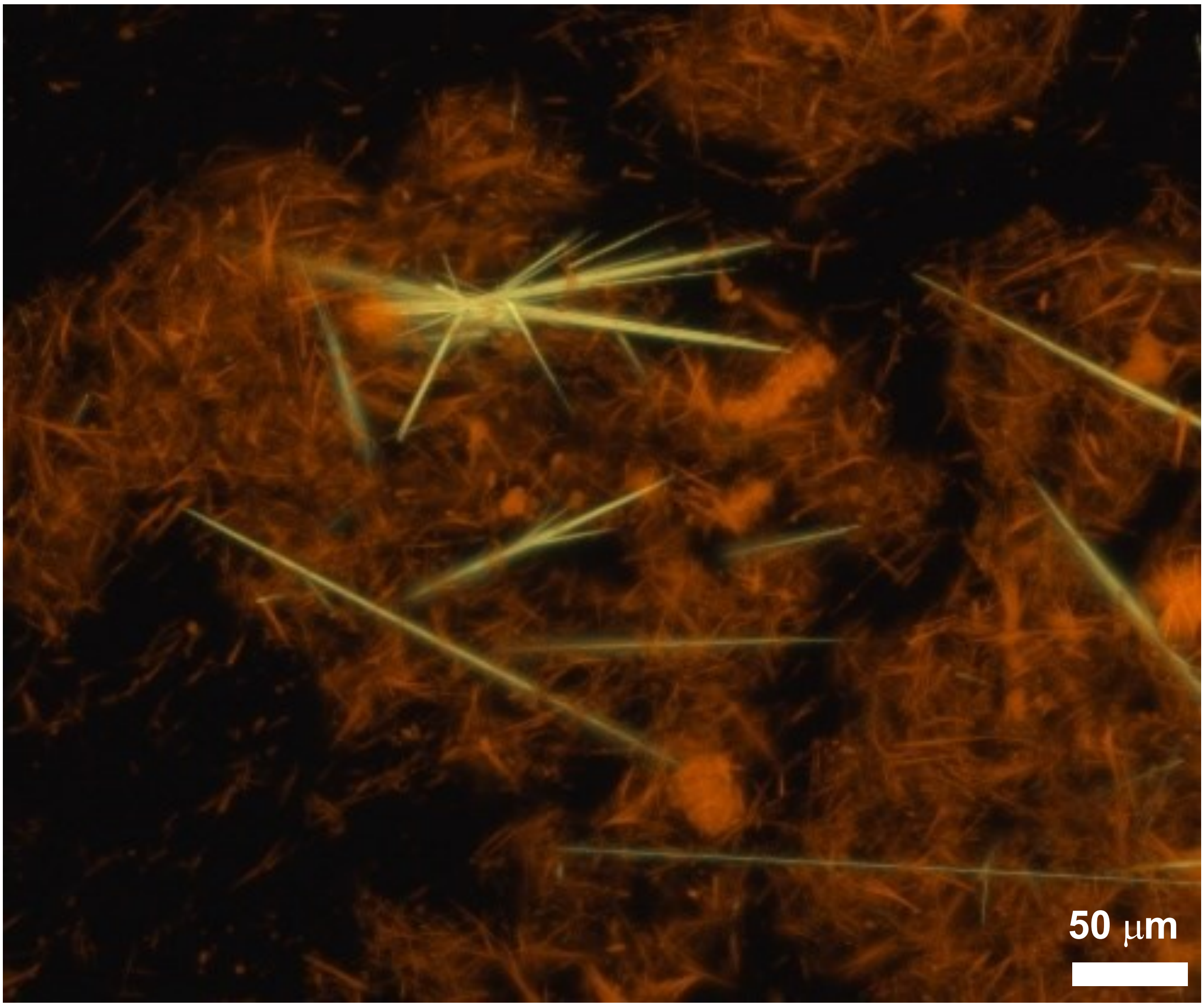

Figure S44. Fluorescence microscopy image of the product obtained from the ligand exchange reaction between complex **3** and ligand **L4**.

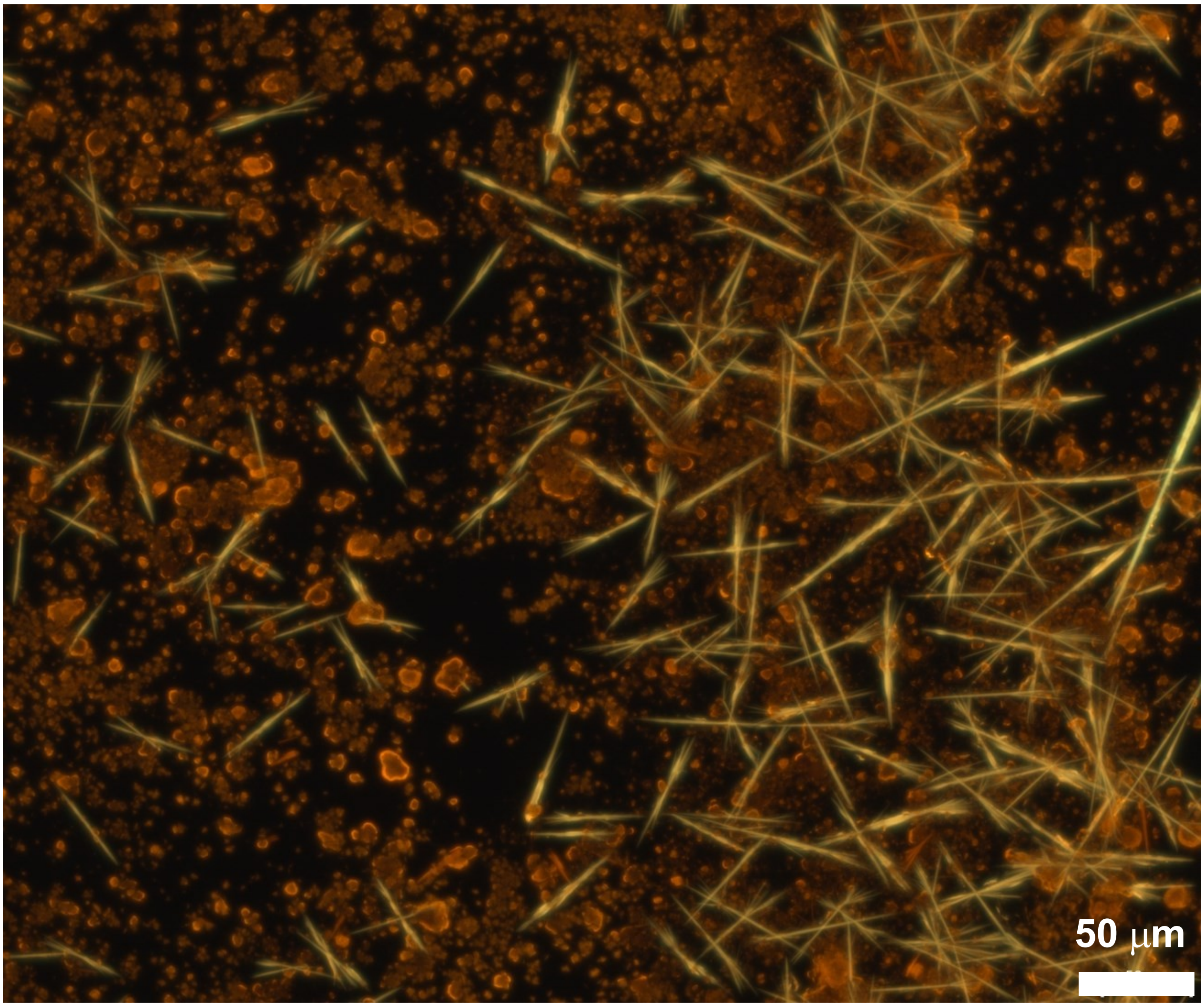

Figure S45. Fluorescence microscopy image of the product obtained from the ligand exchange reaction between complex **4** and ligand **L3**.

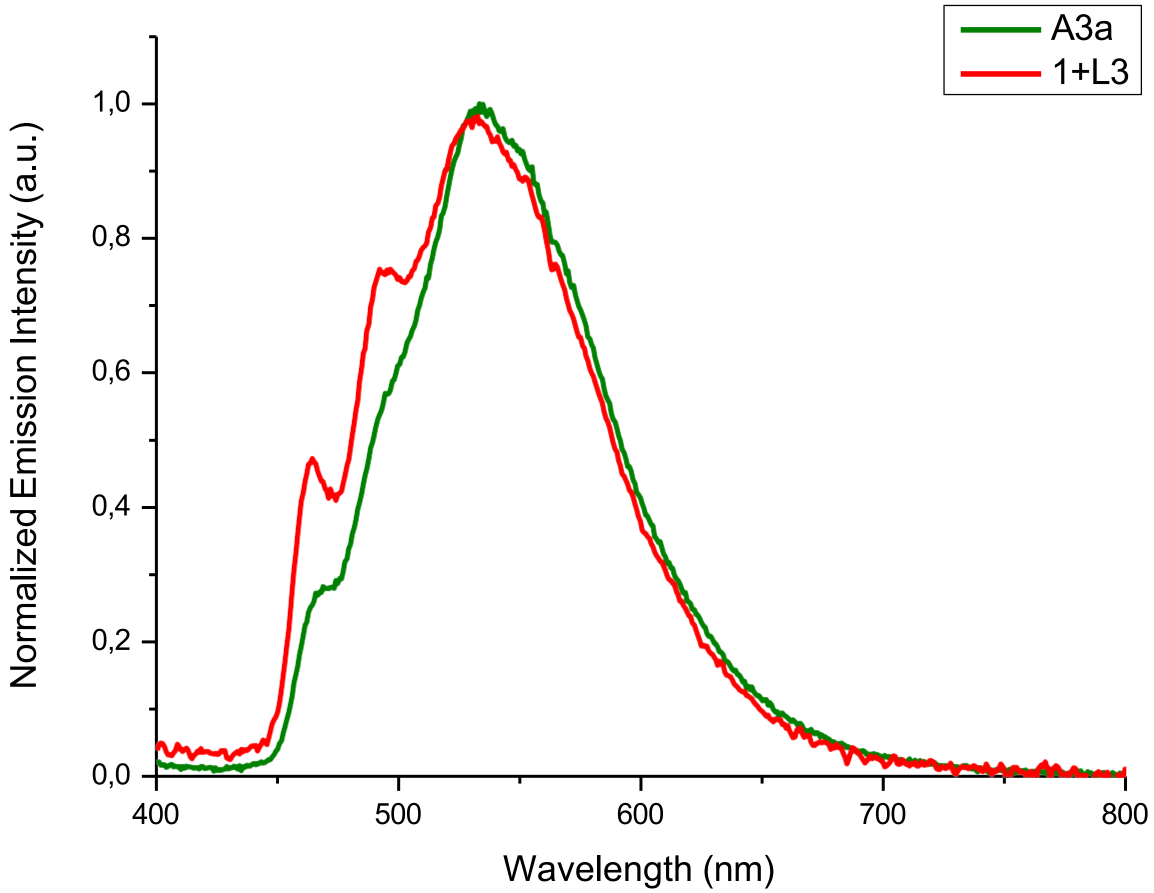

Figure S46. Normalized emission spectrum of the product obtained from the ligand exchange reaction between complex **1** and ligand **L3** (in red) and **A3a** (in green). λ_{exc} = 360 nm.

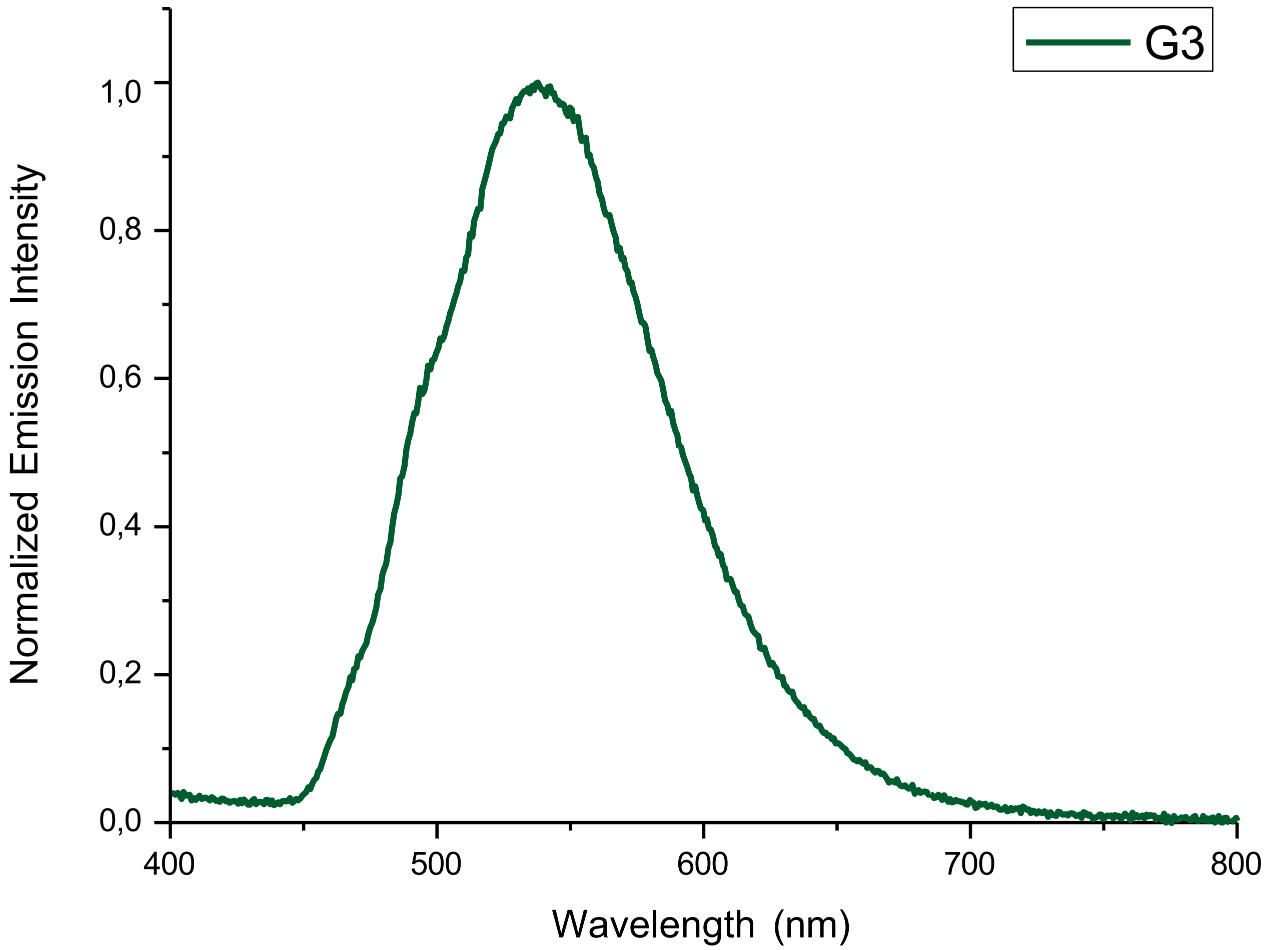

Figure S47. Normalized emission spectrum of **G3,** λ_{exc} = 360 nm.

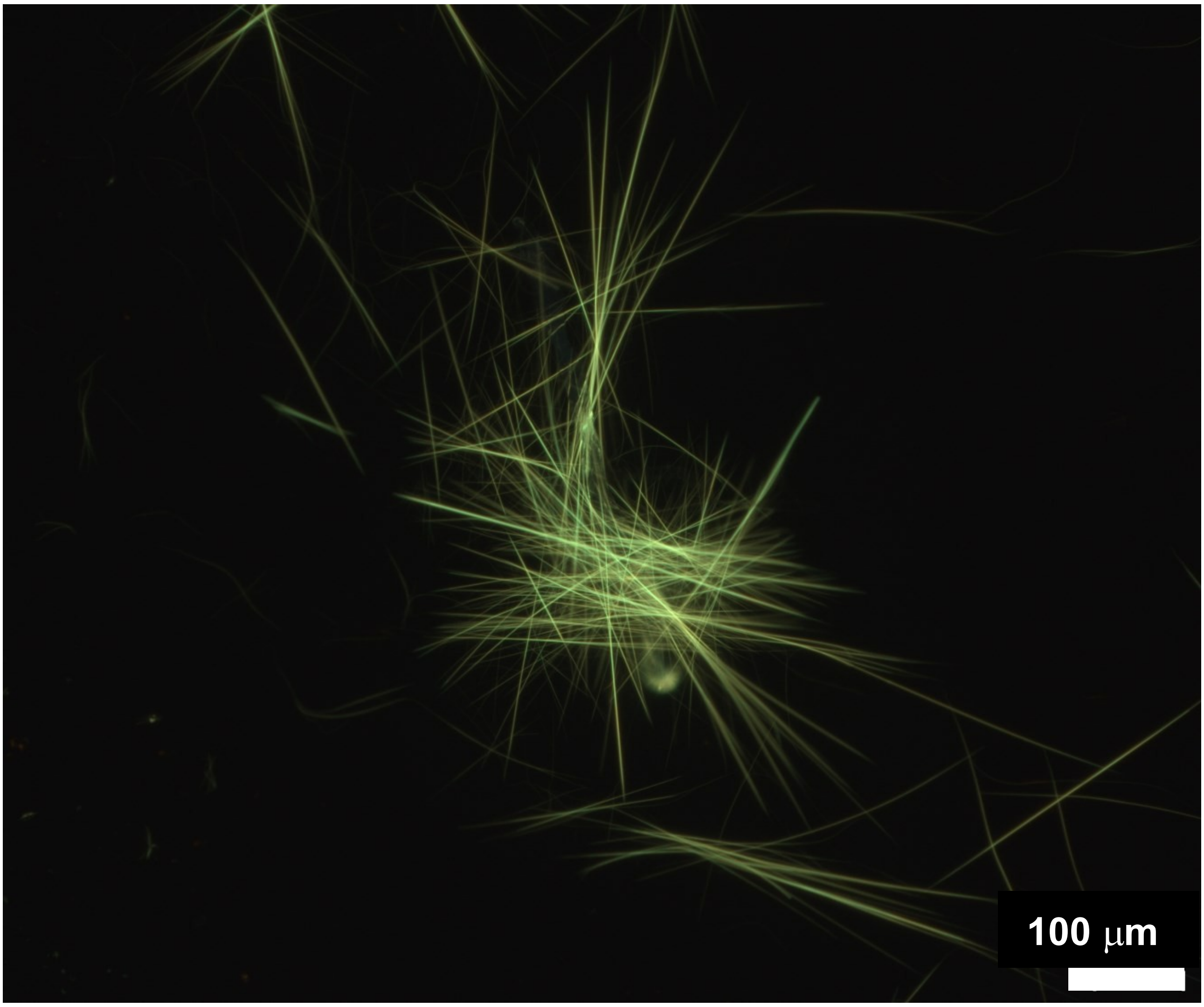

Figure S48. Fluorescence microscopy image of a disrupted **G3.**

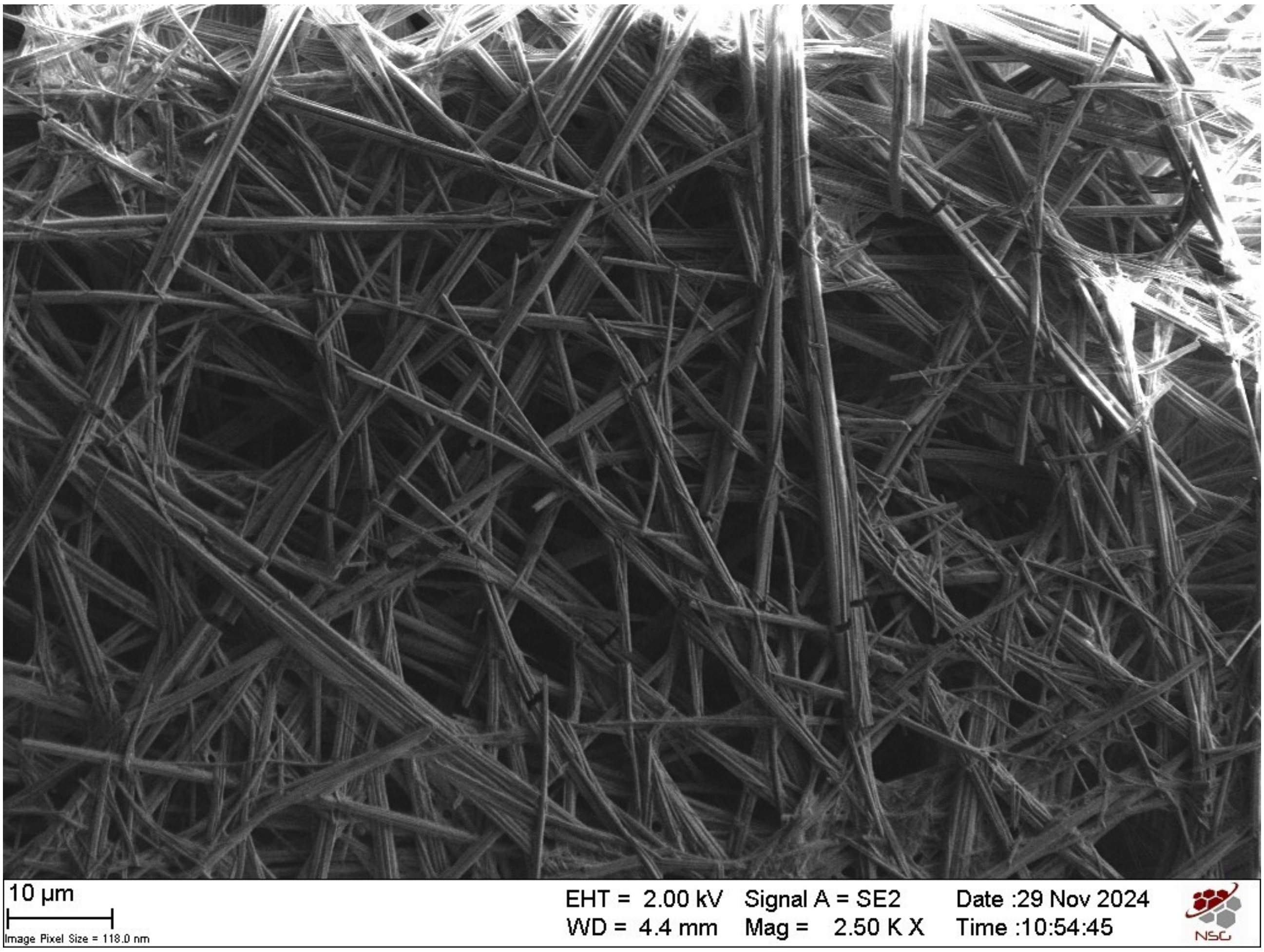

Figure S49. SEM image of **G3.**

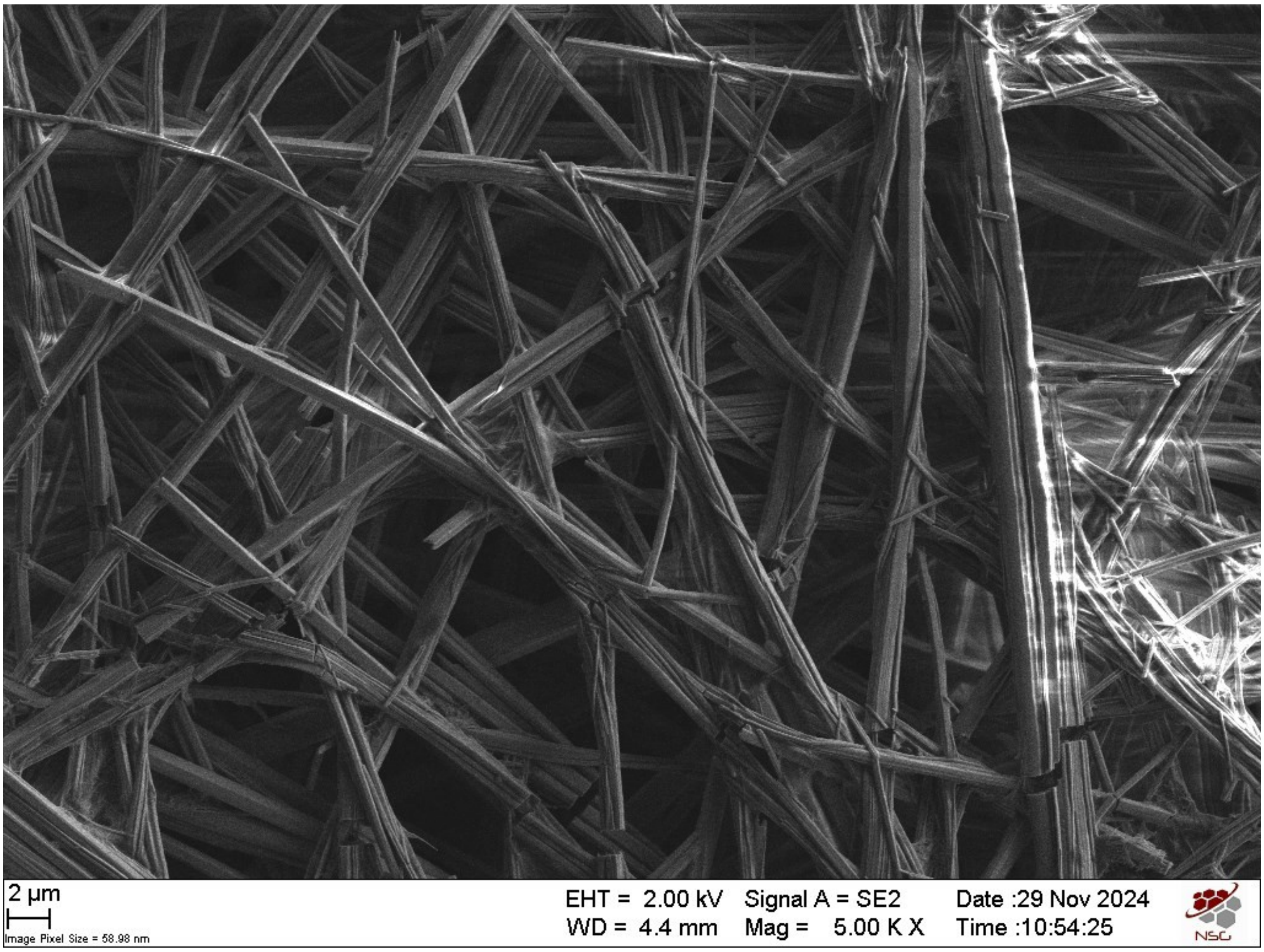

Figure S50. SEM image of **G3.**

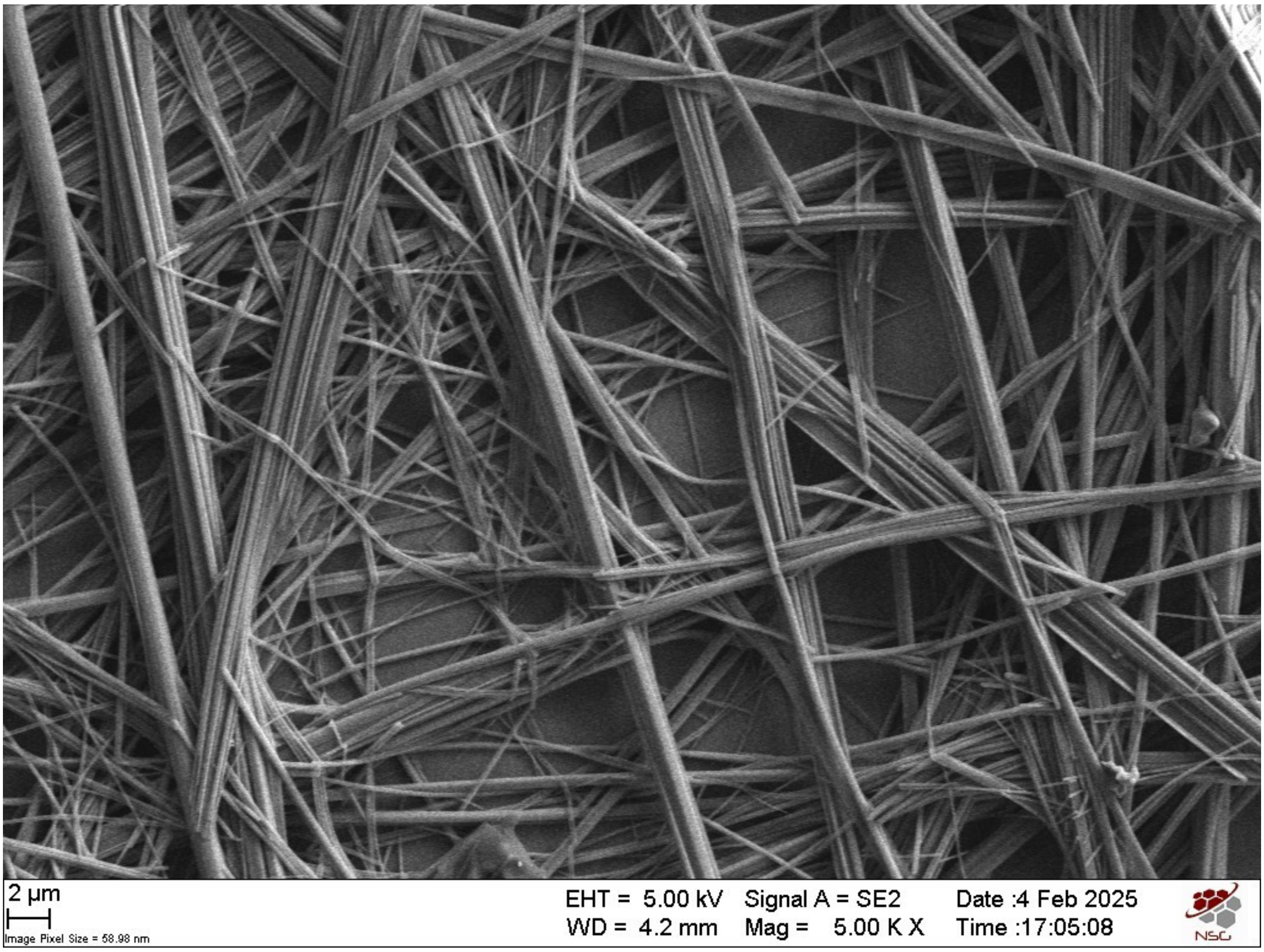

Figure S51. SEM image of ligand exchange product between **1** and **L3.**

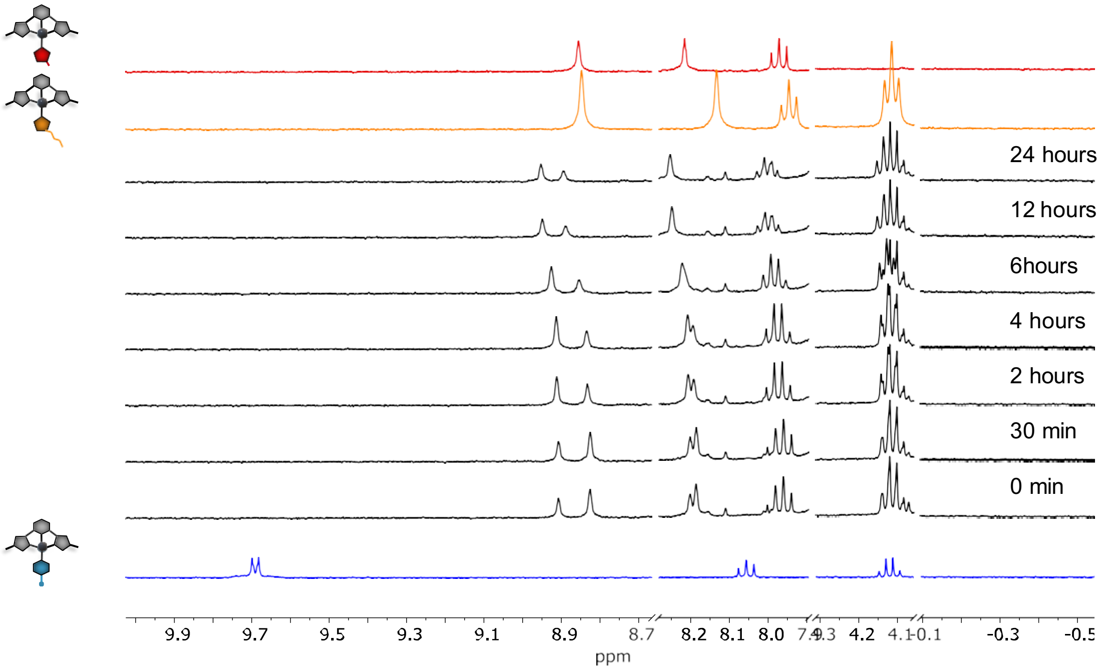

Figure S52. ^{1}H NMR spectra of multi dynamic ligand exchange starting from complex **1** in presence of an excess (5 equivalent each) of **L2-4**. Only characteristic peaks are reported for clarity.

References

(1) Aliprandi, A.; Mauro, M.; De Cola, L. Controlling and Imaging Biomimetic Self-Assembly. *Nat. Chem.* **2016**, *8* (1), 10–15. https://doi.org/10.1038/nchem.2383.
